# A Kernel for Open Source Drug Discovery in Tropical Diseases

**DOI:** 10.1371/journal.pntd.0000418

**Published:** 2009-04-21

**Authors:** Leticia Ortí, Rodrigo J. Carbajo, Ursula Pieper, Narayanan Eswar, Stephen M. Maurer, Arti K. Rai, Ginger Taylor, Matthew H. Todd, Antonio Pineda-Lucena, Andrej Sali, Marc A. Marti-Renom

**Affiliations:** 1 Structural Genomics Unit, Bioinformatics and Genomics Department, Centro de Investigación Príncipe Felipe, Valencia, Spain; 2 Structural Biology Laboratory, Medicinal Chemistry Department, Centro de Investigación Príncipe Felipe, Valencia, Spain; 3 Department of Bioengineering and Therapeutic Sciences, Department of Pharmaceutical Chemistry, and California Institute for Quantitative Biosciences, University of California San Francisco, San Francisco, California, United States of America; 4 Gould School of Law, University of Southern California, Los Angeles, California, United States of America; 5 School of Law, Duke University, Durham, North Carolina, United States of America; 6 The Synaptic Leap, San Ramon, California, United States of America; 7 School of Chemistry, University of Sydney, Sydney, New South Wales, Australia; McGill University, Canada

## Abstract

**Background:**

Conventional patent-based drug development incentives work badly for the developing world, where commercial markets are usually small to non-existent. For this reason, the past decade has seen extensive experimentation with alternative R&D institutions ranging from private–public partnerships to development prizes. Despite extensive discussion, however, one of the most promising avenues—open source drug discovery—has remained elusive. We argue that the stumbling block has been the absence of a critical mass of preexisting work that volunteers can improve through a series of granular contributions. Historically, open source software collaborations have almost never succeeded without such “kernels”.

**Methodology/Principal Findings:**

Here, we use a computational pipeline for: (i) comparative structure modeling of target proteins, (ii) predicting the localization of ligand binding sites on their surfaces, and (iii) assessing the similarity of the predicted ligands to known drugs. Our kernel currently contains 143 and 297 protein targets from ten pathogen genomes that are predicted to bind a known drug or a molecule similar to a known drug, respectively. The kernel provides a source of potential drug targets and drug candidates around which an online open source community can nucleate. Using NMR spectroscopy, we have experimentally tested our predictions for two of these targets, confirming one and invalidating the other.

**Conclusions/Significance:**

The TDI kernel, which is being offered under the Creative Commons attribution share-alike license for free and unrestricted use, can be accessed on the World Wide Web at http://www.tropicaldisease.org. We hope that the kernel will facilitate collaborative efforts towards the discovery of new drugs against parasites that cause tropical diseases.

## Author Summary

Open source drug discovery, a promising alternative avenue to conventional patent-based drug development, has so far remained elusive with few exceptions. A major stumbling block has been the absence of a critical mass of preexisting work that volunteers can improve through a series of granular contributions. This paper introduces the results from a newly assembled computational pipeline for identifying protein targets for drug discovery in ten organisms that cause tropical diseases. We have also experimentally tested two promising targets for their binding to commercially available drugs, validating one and invalidating the other. The resulting kernel provides a base of drug targets and lead candidates around which an open source community can nucleate. We invite readers to donate their judgment and *in silico* and *in vitro* experiments to develop these targets to the point where drug optimization can begin.

## Introduction

There is a lack of high-quality protein drug targets and drug leads for neglected diseases [1],[2]. Fortunately, many genomes of organisms that cause tropical diseases have already been sequenced and published. Therefore, we are now in a position to leverage this information by identifying potential protein targets for drug discovery. Atomic-resolution structures can facilitate this task. In the absence of an experimentally determined structure, comparative modeling can provide useful models for sequences that are detectably related to known protein structures [3],[4]. Approximately half of known protein sequences contain domains that can be currently predicted by comparative modeling [5],[6]. This coverage will increase as the number of experimentally determined structures grows and modeling software improves. A protein model can facilitate at least four important tasks in the early stages of drug discovery [7]: prioritizing protein targets for drug discovery [8], identifying binding sites for small molecules [9],[10], suggesting drug leads [11],[12], and optimizing these leads [13]–[15].

Here, we address the first three tasks by assembling our computer programs into a software pipeline that automatically and on large-scale predicts protein structures, their ligand binding sites, and known drugs that interact with them. As a proof of principle, we applied the pipeline to the genomes of ten organisms that cause tropical diseases (“target genomes”). We also experimentally tested two predicted drug-target interactions using Nuclear Magnetic Resonance (NMR) spectroscopy. By virtue of pairing specific proteins with already known drugs, our pipeline has the potential of increasing the efficiency of target identification, target validation, lead discovery, lead optimization, and clinical trials.

The current project is part of our efforts within the Tropical Disease Initiative (TDI, http://www.tropicaldisease.org) [16]. TDI was conceived as a decentralized and web-based open source drug discovery effort in which academic and corporate scientists volunteer to work together on discovering drugs for neglected diseases. TDI's open source approach complements many new initiatives that have been proposed over the last decade [1], [8], [16]–[25]. However, relatively few volunteers have so far truly engaged in these efforts and their impact is still difficult to assess [26]. Based on our experience with The Synaptic Leap (TSL) online discussion forum of TDI (http://www.thesynapticleap.org), we suggest that a major stumbling block for open source drug discovery has been the absence of a critical mass of preexisting work that volunteers can build on incrementally. Here, we address this bottleneck by introducing a “kernel” to facilitate drug discovery for tropical diseases. This kernel (v1.0) includes 297 potential drug targets from the target genomes and is freely available *via* web 2.0 dissemination tools on the TDI web site.

We begin by describing our computational pipeline as well as the experimental procedures for testing two selected targets (Methods). Next, we describe the modeling of proteins in ten pathogen genomes, prediction of binding of known drugs to the modeled proteins, and experimental testing of these predictions for two select protein targets (Results). Finally, we discuss how we expect a full-scale TDI open source project to use the kernel and its potential impact on open source drug discovery (Discussion).

## Materials and Methods

### Computational pipeline

We have assembled a computational pipeline that relies on several databases and programs, taking as input protein sequences and producing an output containing protein models as well as predicted locations of binding sites for small molecules on their surfaces and predicted types of molecules they bind. The pipeline, which relies on the MODPIPE package [27] and the AnnoLyze program [9], has been applied to genomes of ten pathogens that cause tropical diseases. The output of the pipeline has been stored in a relational database for easy searching and dissemination over the web.

### TDI target genomes

We selected the following ten target genomes based on both disease burden and the completeness of published sequences: *Cryptosporidium hominis* (CyrptoDB [28]), *Cryptosporidium parvum* (CryptoDB [28]), *Leishmania major* (GeneDB [29]), *Mycobacterium leprae* (OrthoMCL-DB [30]), *Mycobacterium tuberculosis* (TubercuList [31]), *Plasmodium falciparum* (PlasmoDB [32]), *Plasmodium vivax* (PlasmoDB [32]), *Trypanosoma brucei* (GeneDB [29]), *Trypanosoma cruzi* (GeneDB [29]), and *Toxoplasma gondii* (ToxoDB [33]). We then mapped the transcript sequences onto UniProt ids [34].

### Annotation databases

Functional annotation for predicted binding sites in our models relied on the following databases: (i) UniProt [34], which contains 385,721 sequences from the SwissProt database and 5,814,087 sequences from the TrEMBL database, was used to annotate the transcripts from the target genomes; (ii) MODBASE [6], which contains 6,805,385 comparative models calculated by MODPIPE for domains in 1,810,521 proteins, was used to store all comparative models; (iii) DBAli [35], which contains 1.7 billion pairwise alignments generated by an all-against-all comparison of known protein structures, was used to identify structure relationships between our modeling templates and other known protein structures; (iv) LigBase [36], which contains 232,852 structurally defined ligand-binding sites in PDB, was used as a resource for AnnoLyze to predict ligand binding sites on pathogen protein models; (v) MSDChem [37], which contains 8,287 small ligands, was used as an annotated repository of small molecules in the PDB database; and (vi) DrugBank [38], which contains 4,765 drug-like compounds (including 1,485 FDA-approved small molecule drugs, 128 FDA-approved biotech drugs, 71 nutraceuticals, and 3,243 experimental drugs), was used to identify small molecules in the MSDChem database that have similar chemical composition to known drugs.

### Comparative protein structure prediction

Models for all sequences from the ten target genomes were calculated using MODPIPE, our automated software pipeline for comparative modeling [27],[39]. It relies primarily on the various modules of MODELLER [40] for its functionality and is adapted for large-scale operation on a cluster of PCs using scripts written in PERL and Python. Sequence-structure matches are established using a variety of fold-assignment methods, including sequence-sequence [41], profile-sequence [42],[43], and profile-profile alignment [43],[44]. Odds of finding a template structure are increased by using an E-value threshold of 1.0. By default, ten models are calculated for each of the alignments [40]. A representative model for each alignment is then chosen by ranking based on the atomic distance-dependent statistical potential DOPE [45]. Finally, the fold of each model is evaluated using a composite model quality criterion that includes the coverage of the modeled sequence, sequence identity implied by the sequence-structure alignment, the fraction of gaps in the alignment, the compactness of the model, and various statistical potential Z-scores [45]–[47]. We only used the models that were predicted to have a “correct” fold (*i.e.*, a MODPIPE quality score higher than 1.0); based on our benchmarking studies, we expect the true positives rate of 93% and the false positives rate of 5%.

### Binding site prediction

**Figure 1:**
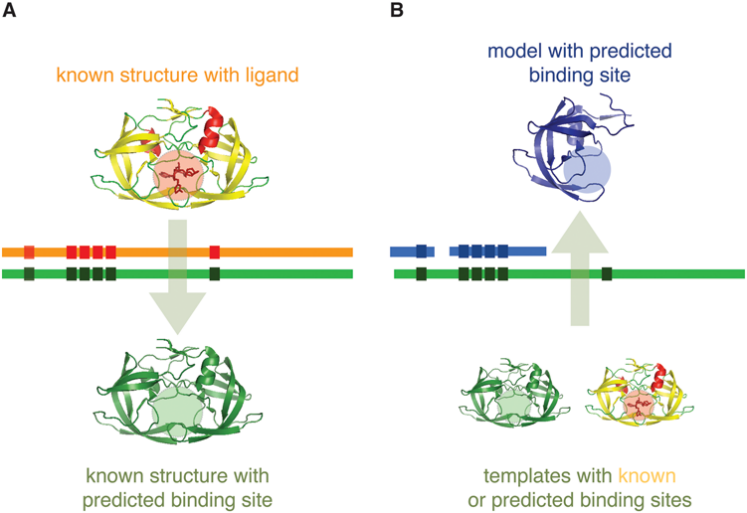
AnnoLyze protocol. (A) Prediction of a binding site in a known structure based on its structural alignment to a known binding site in another structure. (B) Prediction of a binding site in a model based on its structural alignment to a known or predicted binding site in the template structure used to construct the model.

The AnnoLyze program [9] was used to predict binding sites for small molecules on all well-assessed models. Briefly, AnnoLyze predicts ligand-binding sites on the surface of a model by transferring known ligands in the LigBase database [36] *via* the target-template alignment. Such predictions are made in a two step process (Figure 1): (i) transfer of a binding site between known structures (*i.e.* a ligand co-crystallized with a protein structure is transferred to another known structure if at least 75% of the LigBase-defined binding site residues are within 4 Å of the template residues in a global superposition of the two structures and if at least 75% of the binding site residue types are invariant); and (ii) transfer of a binding site to a comparative model using as a reference the alignment to its template (*i.e.* a ligand predicted in the previous step to bind the template or a ligand co-crystallized with the template is transferred to the comparative model if the binding sites are conserved at the same level as in the previous step). Using these cutoffs, approximately 30% of the selected models had at least one predicted binding site for small molecules (Table 1), which were then mapped to MSDChem entries.

**Table 1:** TDI target genomes.

Organism	Disease^a^	DALY^b^	Transcripts^c^	Modeled targets^d^	Coverage^e^	Binding site^f^	Similar^g^	Exact^h^
*Cryptosporidium hominis*	Cryptosporidiosis	n/a	3,886	666	17.14	197	20	13
*Cryptosporidium parvum*			3,806	742	19.50	232	24	13
*Leishmania major*	**Leishmaniasis**	2,090	8,274	1,409	17.03	478	43	20
*Mycobacterium leprae*	**Leprosy**	199	1,605	893	55.64	310	25	6
*Mycobacterium tuberculosis*	**Tuberculosis**	34,736	3,991	1,608	40.29	365	30	10
*Plasmodium falciparum*	**Malaria**	46,486	5,363	818	15.25	284	28	13
*Plasmodium vivax*			5,342	822	15.39	268	24	13
*Toxoplasma gondii*	Toxoplasmosis	n/a	7,793	300	3.85	138	13	6
*Trypanosoma cruzi*	**Trypanosomiasis**	1,525	19,607	3,070	15.66	769	51	28
*Trypanosoma brucei*			9,210	1,386	15.05	458	39	21
**Total**		**85,036**	**68,877**	**11,714**	**17.01**	**3,499**	**297**	**143**

a Diseases in bold are included in the WHO Tropical Disease portfolio.

b DALY, Disability Adjusted Life Year in 1000's, from WHO 2004 health report (http://www.who.int/whr/2004/en/).

c Number of transcripts (*i.e.*, genes that translate into proteins) in each genome.

d Number of targets with at least one domain modeled above the accuracy threshold (*i.e.*, MODPIPE quality score higher or equal to 1.0).

e Percentage of targets in the genome with at least one model above the accuracy threshold (*i.e*., MODPIPE quality score higher or equal to 1.0).

f Number of modeled targets with at least one predicted binding site.

g Number of modeled targets with at least one predicted binding site for a molecule within a 0.9 Tanimoto score to a drug in DrugBank.

h Number of modeled targets with at least one predicted binding site for a molecule in DrugBank.

### From ligands to drugs

The *jcsearch* program from the JChem package [48] was used with default parameters to match related compounds in MSDChem and DrugBank. Four types of matches were collected: (i) exact matches (*i.e.* their SMILES strings [49] matched with a Tanimoto score [50] equal to 1.0); (ii) supra-structure matches in which a matched DrugBank query molecule is a part of an MSDChem molecule; (iii) sub-structure matches in which an identified MSDChem molecule is a part of a DrugBank query molecule; and (iv) similar matches with a Tanimoto score between MSDChem and DrugBank molecules of at least 0.9.

### Protein production and purification

The tested proteins (i.e., a putative thymidylate kinase from P. falciparum and a nucleoside diphosphate kinase from M. leprae) were produced by cloning the full length annotated ORFs into pET47b plasmids (Novagen). The resulting plasmids were purchased from GeneArt (http://www.geneart.com, Regensburg, Germany) and sequenced using conventional methods to confirm the intended constructs were obtained. The proteins were then over-expressed as fusion proteins using BL21 (DE3) Codon plus cells (Strategene). Purification of the proteins was facilitated by a hexa-His tag at the N-terminus and an engineered cleavage site for the TEV protease. Purification to homogeneity was carried out using metal-affinity chromatography (Talon, Clontech), followed by TEV cleavage.

### NMR-based experimental testing of predictions

All spectra were recorded at 300 K with a Bruker Ultrashield Plus 600 MHz NMR spectrometer equipped with a 5 mm TCI cryogenically cooled probe. A typical NMR sample contained a concentration of 5 µM of protein, 100 µM of ligand, 100 µM of glucose as a negative control, 100 mM NaCl, and 25 mM phosphate buffer at pH 7.0.

The concentration of ligand for the Saturation Transfer Difference (STD) experiments was 500 µM. For each sample, a 1D 1H reference, a Water-LOGSY [51] and a STD [52] experiment were recorded. 8 K points were used for a sweep width of 9,600 Hz and a total of 1 K and 512 scans were accumulated for the Water-LOGSY and STD experiments, respectively.

### Data storage, sharing, and licensing

The entire kernel, including all predicted models and binding sites, is freely available over the web (http://www.tropicaldisease.org/kernel). The server uses the WordPress package (http://www.wordpress.org), a widely used platform that facilitates easy creation, storage, and dissemination of each target entry in our database. WordPress supports numerous “plugins”, including a rating system that allows TDI web site users to rate targets for “druggability.” The package also supports bookmarking by most web-based social networks. In particular, each of the TDI kernel's target pages includes a “blog it” button that allows registered users of The Synaptic Leap (TSL, http://www.thesynapticleap.org) to post TDI entries directly into the TSL discussion panels. TSL is our web-based “collaboratory” portal that is designed to host open source drug discovery projects in much the same way SourceForge hosts software collaborations.

The TDI kernel is fully searchable and downloadable through our Web site (http://www.tropicaldisease.org/kernel/). Options include direct downloads of individually requested targets, pre-defined sets for each of our ten target genomes, and user-defined batch downloads. Additionally, all our predictions are available as supporting information files to this article (Datasets S1, S2, S3, S4). Users receive the data with no restriction in accordance with the Science Commons protocol for implementing open access data [53] that was designed to embody normal academic attribution norms and facilitate tracking of work based on the kernel. While our predictions are in the public domain, some of the drugs used in our predictions might be subject to patents.

## Results

### Comparative modeling of protein structures from the ten target genomes

**Figure 2:**
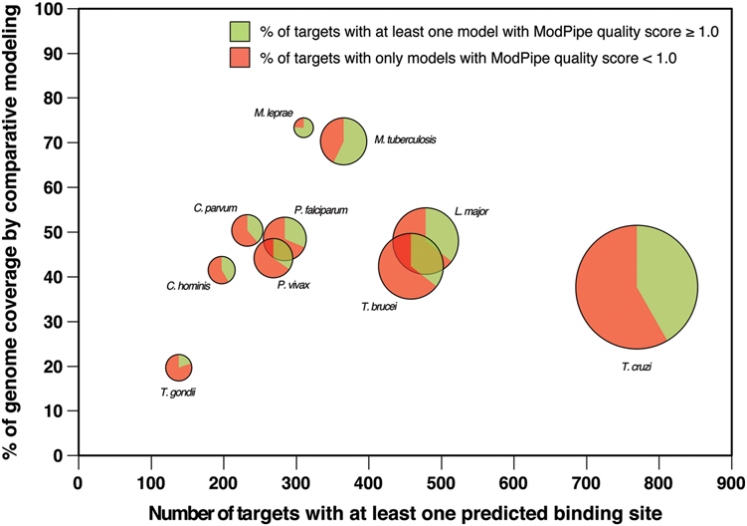
Genome coverage by comparative protein structure models *versus* the number of targets with at least one predicted binding site for a small molecule. Pie charts for each of the ten target genomes indicate the percentage of targets with at least one model above and below the accuracy threshold (*i.e.*, MODPIPE quality score 1.0) in the green and red colors, respectively. The total area of each pie chart is proportional to the corresponding genome size.

The accuracy of our comparative protein structure models built using MODPIPE was predicted by a variety of criteria, including target-template sequence identity, coverage of the target sequence, fraction of gaps in the alignment, and statistical potential scores. One third of the total models (21,031) were assessed to have sufficient accuracy for predicting the location and type of their binding sites for small compounds (*i.e.*, at least 50% of their Cα atoms are predicted to be within 3.5 Å of their correct positions, corresponding to the correct fold and at least an approximately correct alignment with the template structure). These models covered 11,714 protein targets, corresponding to 17% of all proteins in the ten target genomes (Table 1 and Figure 2). There are an average of ∼2.5 models per protein target, each model potentially based on a different template structure and/or covering a different domain of the modeled sequence. Different genomes presented different levels of difficulty to our modeling procedure: 75% of the models for *M. leprae* proteins met our accuracy standards, while only approximately 10% of *T. gondii* models did. These coverage correspond to accurate predictions for 3,070 targets in *Trypanosoma cruzi* (15.7% of the genome) and 300 targets (3.9% of the genome) for *T. gondii* (Table 1).

### Predicted binding sites in comparative models

We applied our AnnoLyze program to predict the binding sites for small molecules in the MSDChem database on 11,714 well-modeled targets. A total of 3,499 (∼30%) of these targets had a predicted binding site from their comparative modeling template or a known binding site transferred from a structurally similar protein. Once again, the *T. cruzi* genome had the largest number of predicted binding sites located in 769 targets, while *T. gondii* contained only 138 targets with a predicted small-molecule binding site (Table 1 and Figure 2). In general, there was an almost linear relationship between the genome size and the number of targets with predicted binding sites. The *M. leprae* genome provided a notable exception, with accurate models covering domains in 55.6% of the proteins and predicted binding sites for a small molecule in only 310 of these targets.

### Comparison of results for the ten target genomes

The coverages of comparative modeling and ligand binding site prediction vary from one genome to another (Table 1). For example, *T. gondii* has poor structure coverage of its 7,793 genes predicted in ToxoDB (3.85%). This poor structural coverage may be partly a result of a relatively inaccurate current assignment of genes, as suggested by differences between four methods for predicting genes from a genome [54]; these annotations agreed in only 12% of the genes. Moreover, 3,837 genes in ToxoDB are poorly annotated with keywords such as “hypothetical”, “putative”, and “predicted”. In contrast, *M. leprae*, which is a minimal mycobacterial genome [55], resulted in the highest coverage of all target genomes (55.64%). This high coverage is a consequence of a larger proportion of its sequences having homologs whose complexes with small molecules have been defined structurally. Finally, there is an artificially large number of predictions for *T. cruzi*. The *T. cruzi* genome was sequenced from a hybrid strain from two divergent parental lines [56], which resulted in a large number of its genes with duplicated entries in the GeneDB database.

Given that our computational pipeline relies on homology for predicting the structure and binding sites of a query sequence, we analyzed the predictions across ortholog sequences from the ten target genomes. A total of 236 of the 297 selected targets group into 46 ortholog groups as defined by the OrthoMCL-DB database [30]. Our predictions agreed for 38 of the 46 ortholog groups (*i.e.*, the same ligands were predicted to bind all the orthologs within the cluster). Only 4 of the 46 ortholog groups resulted in a complete disagreement (*i.e.*, all orthologs resulted in different predicted binding ligands). Finally, the remaining 4 ortholog groups had intermediate results (*i.e.*, some but not all of the orthologs in the cluster were predicted to bind the same ligand).

### Protein targets predicted to bind known drugs

To link small molecules from MSDChem to chemical compounds in DrugBank, we used JChem to perform an all-against-all comparison of the SMILES strings from both databases (Table 1). This linking allowed us to predict 297 proteins that are likely to bind a known drug from DrugBank or a compound similar to it (*i.e.*, with a Tanimoto score of at least 0.9); 143 of these targets were predicted to have a binding site for a known drug (*i.e.*, a Tanimoto score of 1.0). Next, we outline two predictions that make sense in the light of the known antiprotozoal activity of the corresponding drugs.

**Figure 3:**
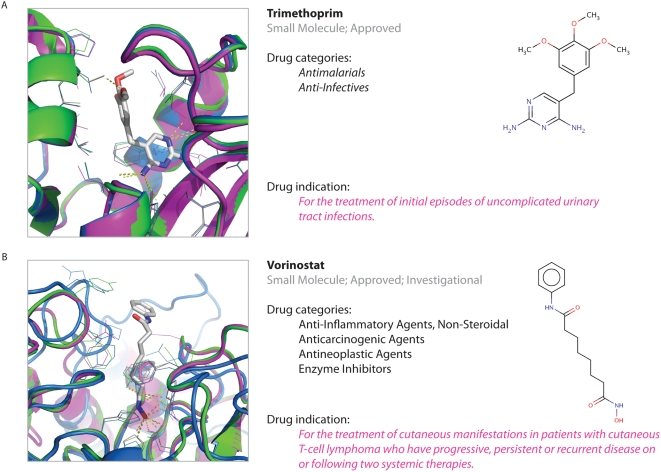
Examples of known antiprotozoal drugs detected by our method. (A) Trimethoprim drug predicted to bind *M. leprae* dihydrofolate reductase (UniProt identifier Q9CBW1). (B) Vorinostat drug predicted to bind *L. major* histone deacetylase (UniProt identifier Q4QCE7). The original PDB structure with the ligand bound is shown in blue; the transferred binding site in the template structure is shown in green; and a comparative protein structure model of the target sequence is shown in magenta.

Our pipeline correctly predicted that the known antiprotozoal drug Trimethoprim (DrugBank identifier DB00440) interacts with a dihydrofolate reductase (UniProt identifier A1QV37) in *Mycobacterium tuberculosis*. Trimethoprim is a pyrimidine-like inhibitor of dihydrofolate reductases that acts as an antibacterial agent and has weak antimalaria activity [57]. Moreover, our predictions suggest that Trimethoprim might also inhibit a dihydrofolate reductase from *M. leprae* (UniProt identifier Q9CBW1), given that its binding site is 93.3% identical in sequence to that of dihydrofolate reductase from *M. tuberculosis* (Figure 3A).

In a second example, our predictions shed light on the molecular mechanism of aroyl-pyrrolyl-hydroxyamides, a class of histone deacetylase inhibitors, which have previously been reported to have antileishmanial activity [58],[59]. Although the structure of *Leishmania major*'s histone deacetylase is unknown (UniProt identifier Q4QCE7), it can be modeled using the structure of the human histone deacetylase as a template (sequence identity is 36.0%). Using the ligand binding site prediction protocol of AnnoLyze, we predict a binding site for SSH (octanedioic acid hydroxyamide phanylamide) in the human histone deacetylase (PDB identifier 1t64A), as found in the *Aquifex aeolicus* histone deacetylase (PDB identifier 1c3sA). The coverage and sequence identity of the binding site for SHH, which is an exact match to the drug Vorinostat (DrugBank identifier DB02546), was 100.0% and 90.9%, respectively. Thus, our predictions suggest molecular details of Vorinostat's mechanism of action as an inhibitor of *L. major* histone deacetylase (Figure 3B).

### Experimental testing of targets Q8I4S1 and Q9CBZ0

Two additional predicted drug targets were used to test our computational methods using NMR spectroscopy: (i) a putative thymidylate kinase from *Plasmodium falciparum* (UniProt identifier Q8I4S1) predicted to bind Zidovudine (a nucleoside reverse transcriptase inhibitor) and (ii) a nucleoside diphosphate kinase from *M. leprae* (UniProt identifier Q9CBZ0) predicted to bind Fludarabine (a DNA polymerase alpha, ribonucleotide reductase and DNA primase inhibitor). Both targets were selected based on the feasibility of NMR experiments (*i.e.*, protein shorter than 250 amino acid residues in length), non-trivial modeling (*i.e.*, the target and the template were globally aligned with less than 75% sequence identity), and non-trivial prediction of the ligand (*i.e.*, using only similarity matches).

Thymidylate kinases (TMPK) catalyze the reversible phosphorylation of deoxythymidine monophosphate (dTMP) to deoxythymidine diphosphate (dTDP) and are essential for the survival of the organism. In particular, the TMPK from *P. falciparum* was recently expressed and biochemically characterized in terms of its molecular affinity to several substrates and appears to be a good target for drug discovery, especially for binding to purine-based inhibitors [60]. We predicted that TMPK from *P. falciparum* binds ATM (3′-azido-3′-deoxythymidine-5′-monophosphate). ATM is highly similar to Zidovudine, which lacks only the 5′ monophosphate of ATM. Zidovudine is a dideoxynucleoside that prevents the formation of phosphodiester linkages needed for the completion of nucleic acid chains. It has been used as a potent inhibitor of HIV replication, acting as a chain-terminator of viral DNA during reverse transcription. An experimental structure of *P. falciparum* TMPK is not available, but can be predicted by comparative modeling based on 41% sequence identity to a known structure of the yeast TMPK (PDB identifier 3tmkA). 3tmkA also has a predicted binding site for ATM, which was transferred from another crystallized structure of yeast TMPK (PDB identifier 2tmkA).

**Figure 4:**
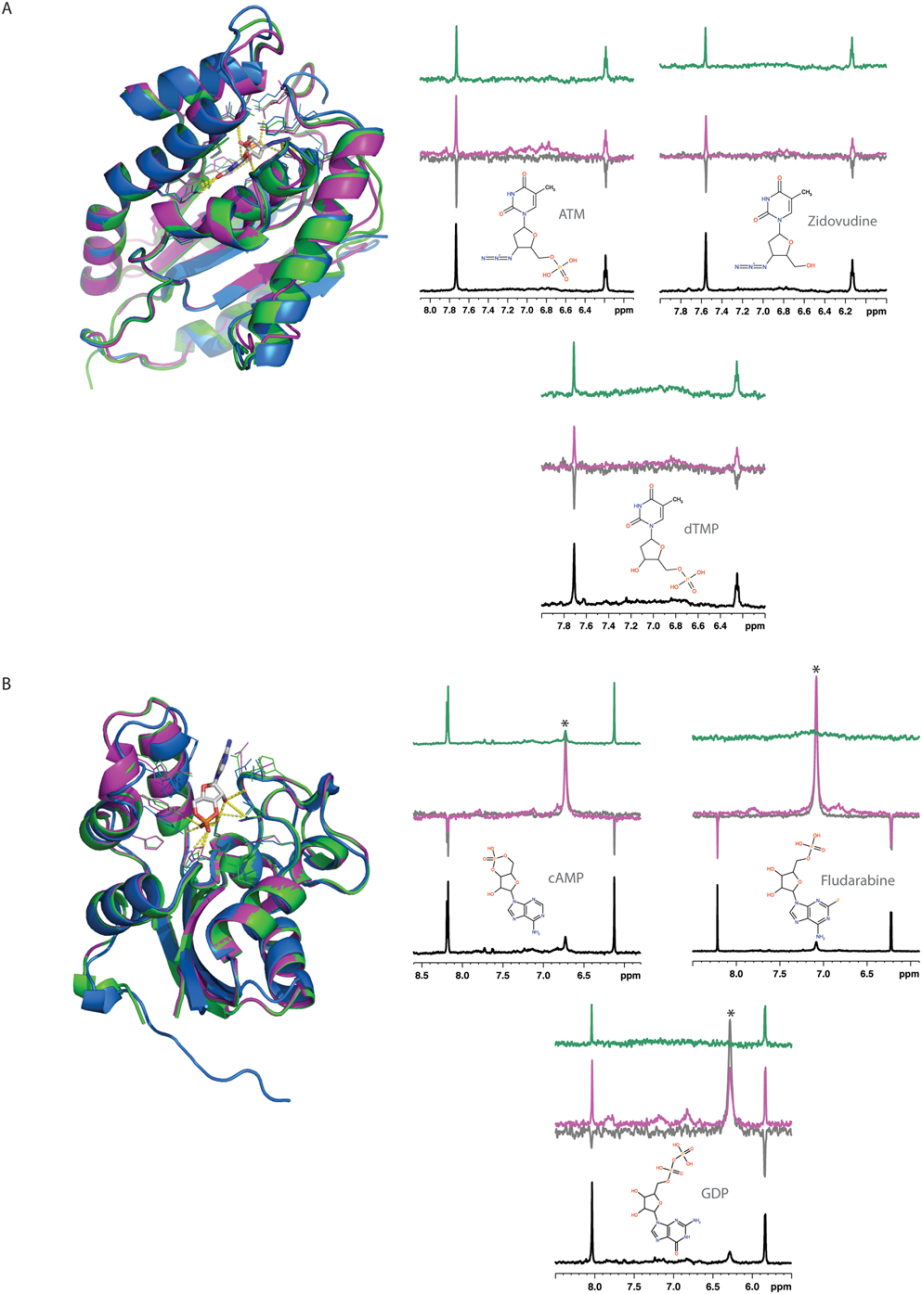
Experimental validation of two predicted target-ligand pairs. (A) *P. falciparum* thymidylate kinase (UniProt identifier Q8I4S1) interactions with dTMP, ATM and Zidovudine. (B) *M. leprae* nucleoside diphosphate kinase (UniProt identifier Q9CBZ0) interactions with GDP, cAMP and Fludarabine. Structures colored as in Figure 2. Each NMR spectrum shows a detail of the aromatic region for the interacting molecules, the bottom spectra corresponding to the reference 1D ^1^H experiment (black line). In this experimental setting, a non-interacting compound results in negative resonances in the Water-LOGSY experiment and no signals in the STD spectrum. In contrast, protein-ligand interactions in the Water-LOGSY (magenta line) are characterized by positive signals or by a reduction in the negative signals obtained in the absence of the protein (reference spectrum, grey line). In the STD experiment, a positive interaction is recognized by the presence of positive signals (green line). Signals marked with an asterisk arise from exchangeable protons, and although positive, do not indicate an interaction between the protein and the ligand, as they also show the same behavior in the absence of protein.

Using NMR Water-LOGSY and STD experiments, we have tested the binding capacity of both ATM and Zidovudine to the surface of *P. falciparum* TMPK. In the Water-LOGSY experiments, the large bulk water magnetization is partially transferred *via* the protein-ligand complex to the free ligand in a selective manner. As a consequence, the resonances of the ligand have a sign opposite to that of non-interacting compounds; their signal also appears stronger. To test the applicability of the Water-LOGSY experiment to *P. falciparum* TMPK, we tested glucose as a negative control (*i.e.*, non-interacting ligand) and dTMP as a positive control (*i.e.*, a known ligand for TMPK), resulting in the expected negative and positive interacting signals, respectively (Figure 4A). With this validation in hand, similar experiments were performed with ATM and Zidovudine. Both ATM and Zidovudine result in positive Water-LOGSY signals, confirming their predicted interaction with P. falciparum TMPK. The results were further validated by the positive signals in the STD spectra that are better suited for detecting interactions between strong binders and proteins.

Nucleoside diphosphate kinases (NDK) have major roles in the synthesis of nucleoside triphosphates other than ATP. In particular, the NDK from *M. leprae* was predicted to bind cAMP (adenosine-3′,5′-cyclic-monophosphate). cAMP has a similar structure to the known drug Fludarabine, which inhibits DNA synthesis and has been used in chemotherapy for the treatment of hematological malignancies. We built a comparative model of *M. leprae* NDK based on 58% sequence identity to the NDK form *Thermus thermophilus* (PDB identifier 1wkjA). 1wkjA has a predicted binding site for cAMP, based on its similarity to *Myxococcus xanthus* NDK (PDB identifier 1nhkR), which is known to bind cAMP.

As for TMPK, we used Water-LOGSY and STD experiments to determine whether or not cAMP and Fludarabine bind to the surface of *M. leprae* NDK. For this target, glucose and GDP, a known NDK ligand, were used as negative and positive controls, respectively (Figure 4B). The Water-LOGSY experiments showed an almost undetectable interaction, between cAMP and NDK. This finding was confirmed by the STD experiment. However, neither of the experiments resulted in positive signs in the NMR spectra of the interaction between Fludarabine and NDK, invalidating our prediction.

## Discussion

Identifying targets and lead compounds that have good odds for surviving clinical trials is one of the most challenging tasks facing the pharmaceutical industry. This challenge is particularly urgent in the neglected disease context where the upstream end of the development pipeline is in danger of drying up [1]. Here, we have introduced a new computational pipeline that generates comparative models of input protein sequences, the location of small molecule binding sites on these models, and the types of compounds that bind to them. We have applied this pipeline to ten complete genomes of pathogens causing neglected diseases and the set of compounds in the DrugBank database, which contains both known drugs and related molecules. Using NMR spectroscopy, we have also experimentally tested two predictions, validating one of them. The high efficiency and coverage of our computational methods is particularly important for tropical disease research, where commercial markets are too small to support conventional patent-based research models. Identifying new protein targets and previously developed drugs that interact with them have the potential of greatly simplifying experimental validation of these new targets, lead optimization, and clinical trials. Moreover, our approach can lead to characterizations of the mechanism of action of already known drugs. Because tropical diseases affect millions of people, the stakes could not be higher.

A total of 68,877 protein sequences encoded by ten genomes were input into MODPIPE, resulting in models for 11,714 (17%) target sequences that were estimated to be sufficiently accurate for predicting the location and type of binding sites on their surfaces. With these models in hand, AnnoLyze, our binding site prediction program, was able to predict a binding site for a small molecule on 3,499 potential targets, of which 297 were predicted to bind a molecule similar to a known drug, including 143 predicted to bind a known drug. These protein targets, available through the TDI's kernel web site (http://www.tropicaldisease.org/kernel/), can be regarded as “low hanging fruits” for drug discovery in tropical diseases.

Using NMR spectroscopy, we have experimentally tested whether or not two of these targets actually bind their predicted drug ligands. While our experiments have not tested for either binding site localization or binding affinity, they do confirm that the drug Zidovudine indeed interacts with a *P. falciparum* thymidylate kinase. In contrast, the prediction of the binding of Fludarabine to *M. leprae* nucleoside diphosphate kinase was invalidated. This prediction was based on the relatively low conservation of the predicted binding site (75% sequence identity between the binding site residues in the template and target), indicating that such predictions should be treated with caution.

The key contribution of this work results from the structural analysis of putative binding sites in the surface of protein structure models of genes from ten organisms that cause tropical diseases. However, it is not clear how to assess the false positives and false negatives rates for our computational method based on the existing experimental information. Our understanding of errors in comparative modeling [9] and in similarity-based transfer of functional sites between homologs [4], combined with the limited experimental validation reported here, suggests that a useful fraction of predictions are correct. We urge other investigators to donate their expertise and facilities to validate our many predictions, within the open source context.

The main goal of our exercise was to narrow down the number of targets and identify their putative ligands for experimental follow-up, so that the overall process is faster, more thorough, and less expensive. We see the TDI kernel only as a beginning. For example, our methods not only predict plausible ligands for a target, but also localize the binding site on the surface of the protein, a necessary step for further leveraging our results for optimizing the lead compounds by a combination of computational and experimental methods, such as computational docking, site-directed mutagenesis, and synthetic chemistry. We also recognize that the kernel's list of “hits” does not even remotely exhaust the ten target genomes. Researchers who want TDI to investigate additional candidates (whether or not previously published) should contact us or engage in online discussions (http://www.thesynapticleap.org). Moreover, our TDI and TSL web sites provide a full suite of Web 2.0 tools for disseminating the kernel for further annotation.

It would be counterproductive for TDI to patent or otherwise seek intellectual property rights in these discoveries. Of course, there is no guarantee that others do not claim such rights. For example, some of the drugs in DrugBank may be the subjects of patents. Nevertheless, the existence of unpatented targets and at least some unpatented compounds will give sponsors bargaining power in negotiations with patent owners, if they demanded excessive royalties. The net result will be to reduce the royalties that patent owners can charge and sponsors must pay.

Many open source licenses contain “viral” terms, which limit users' ability to seek intellectual property of their own. In the case of drug discovery, however, such strategies are likely to be expensive and, in some cases, legally dubious [61],[62]. Nevertheless, these obstacles are not fatal and one can imagine schemes in which discoveries are embargoed for months or years, so that access is limited to those who promised not to seek patents of their own [63]. We have decided against trying to impose a viral condition on subsequent researchers. First and foremost, open source requires as many workers, volunteer and commercial, as possible, implying minimal restrictions on the data, including viral terms. Second, at least some of the organisms included in the kernel (*e.g.*, *M. tuberculosis*) have potential commercial markets large enough to offset a fraction of sponsors' R&D costs. Nevertheless, it is still possible that an unscrupulous corporation, for example, could try to patent trivial improvements to the kernel. This, however, seems unlikely in the impoverished world of neglected disease research, at least for the immediate future. In the meantime, we prefer to leave the question open until open source collaboration has been firmly established. That will put the final responsibility where it belongs – with the volunteers whose labor and insights we are depending on to turn TDI's kernel into safe, effective, and affordable cures.

## Supporting Information

Dataset S1 PDF version of the data for selected targets
(0.27 MB PDF)

Dataset S2 Tab separated version of the data for selected targets
(0.13 MB TXT)

Dataset S3 Excel version of the data for selected targets
(0.36 MB XLS)

Dataset S4 MySQL version of the data for selected targets
(0.14 MB TXT)
