# Supplementary material for: A Kernel for Open Source Drug Discovery in Tropical Diseases
Source: PLoS Negl Trop Dis. 2009 Apr 21;3(4):e418. doi: 10.1371/journal.pntd.0000418 (PMC2667270; doi:10.1371/journal.pntd.0000418)
Supplement: Dataset S1 — PDF version of the data for selected targets (0.27 MB PDF) [file pntd.0000418.s001.pdf]

Organism	UniProt Id	Target description	3D model id	PDB Template	Model sequence identity	ModPipe Quality Score	PDB Expansion id	Binding site expansion sequence identity	Binding site expansion structure identity	Binding site sequence identity	Binding site structure identity	PDB Ligand id	Drugbank (Exact)	Drugbank (supra-structure)	Drugbank (sub-structure)	Drugbank (similar)
Mycobacterium tuberculosis	A1QNP2	Mycolic acid synthase pcaA (Cyclopropane synthase).	57116736.2.pdb	1tpyA (TRANSFERASE)	73	2.05	1kpgA (TRANSFERASE)	78.26	100	77.27	100	16A				DB01245 (Decamethonium)
Mycobacterium tuberculosis	A1QQL7	Putative uncharacterized protein (Hypothetical short-chain typedehydrogenase/ reductase).	15608284.2.pdb	1uayA (OXIDOREDUCTASE)	61	1.81	1e6wC (DEHYDROGENASE)	80	100	88.89	100	EST	DB00783 (Estradiol)	DB00783 (Estradiol)	DB04573 (Estriol) DB00783 (Estradiol)	DB04573 (Estriol) DB00977 (Ethinyl Estradiol) DB00783 (Estradiol)
Mycobacterium tuberculosis	A1QR09	Bifunctional enzyme cysN/cysC: sulfate adenyltransferase (Subunit 1) +adenylylsulfate kinase (Bifunctional enzyme cysN/cysC: sulfateadenyltransferase subunit 1 + adenylylsulfate kinase).	15608426.3.pdb	2ax4A (TRANSFERASE)	51	1.05	1m7hB (TRANSFERASE)	94.74	100	76.19	100	ADX				DB01073 (Fludarabine)
Mycobacterium tuberculosis	A1QU84	Nucleoside diphosphate kinase ndkA.	15609582.1.pdb	1npk_ (PHOSPHOTRANSFERASE(PO4 AS ACCEPTOR))	52	1.73	1nhkR (PHOSPHOTRANSFERASE)	84.62	100	75	100	CMP				DB01073 (Fludarabine)
Mycobacterium tuberculosis	A1QUK4	Aspartyl-tRNA synthetase aspS.	15609709.1.pdb	1l0wA (LIGASE)	52	1.59	1g51B (LIGASE)	100	100	90.62	100	AMO				DB01073 (Fludarabine)
Mycobacterium tuberculosis	A1QV37	Dihydrofolate reductase dfrA (Tetrahydrofolate dehydrogenase).	15609900.1.pdb	1df7A (OXIDOREDUCTASE)	100	2.26	1df7A (OXIDOREDUCTASE)	100	100	100	100	MTX	DB00563 (Methotrexate)	DB00563 (Methotrexate)	DB00563 (Methotrexate)	DB00563 (Methotrexate)
Mycobacterium tuberculosis	A1QV37	Dihydrofolate reductase dfrA (Tetrahydrofolate dehydrogenase).	15609900.1.pdb	1df7A (OXIDOREDUCTASE)	100	2.26	1dg5A (OXIDOREDUCTASE)	100	100	100	100	TOP	DB00440 (Trimethoprim)	DB00440 (Trimethoprim)	DB00440 (Trimethoprim)	DB00440 (Trimethoprim)
Mycobacterium tuberculosis	A1QXF3	Biphenyl-2,3-diol 1,2-dioxygenase bphC.	15610704.1.pdb	1lgtA (OXIDOREDUCTASE)	39	1.3	1gdgA (OXIDOREDUCTASE)	100	100	100	100	NO	DB00435 (Nitric Oxide)	DB00435 (Nitric Oxide)	DB00435 (Nitric Oxide)	DB00435 (Nitric Oxide)
Mycobacterium tuberculosis	A1QXF3	Biphenyl-2,3-diol 1,2-dioxygenase bphC.	15610704.3.pdb	1kw3B (OXIDOREDUCTASE)	35	1.49	1kw8B (OXIDOREDUCTASE)	100	100	100	100	NO	DB00435 (Nitric Oxide)	DB00435 (Nitric Oxide)	DB00435 (Nitric Oxide)	DB00435 (Nitric Oxide)
Toxoplasma gondii.	A3FKJ9	PMR1-like calcium ATPase.	583.m05378.1.pdb	1ygpA (GLYCOSYLTRANSFERASE)	50	1.63	2ecpB (ACARBOSE)	100	100	100	100	ACR	DB00284 (Acarbose)	DB00284 (Acarbose)	DB00284 (Acarbose)	DB00284 (Acarbose)
Cryptosporidium parvum Iowa II.	A3FPQ3	Small nuclear ribonucleoprotein, putative (Fragment).	cgd7_3970.1.pdb	1th7A (RNA BINDING PROTEIN)	44	1.56	1i5lI (RNA BINDING PROTEIN/RNA)	100	100	77.78	100	URI				DB00322 (Floxuridine)
Cryptosporidium parvum Iowa II.	A3FPS4	OSK4, putative.	cgd6_650.3.pdb	2h6dA (SIGNALING PROTEIN,TRANSFERASE)	57	1.06	1fmoE (COMPLEX (PHOSPHOTRANSFERASE/INHIBITOR))	82.35	89.47	87.5	100	ADN			DB00194 (Vidarabine)	DB00242 (Cladribine) DB00194 (Vidarabine)
Cryptosporidium parvum Iowa II.	A3FQ39	Protein kinase, cAMP-dependent, catalytic chain.	cgd3_3040.2.pdb	1rdqE (TRANSFERASE/TRANSFERASE INHIBITOR)	54	1.6	1fmoE (COMPLEX (PHOSPHOTRANSFERASE/INHIBITOR))	100	100	94.44	100	ADN			DB00194 (Vidarabine)	DB00242 (Cladribine) DB00194 (Vidarabine)
Cryptosporidium parvum Iowa II.	A3FQ56	Calmodulin.	cgd2_810.3.pdb	2amiA (CELL CYCLE)	58	1.38	1lin_ (CALCIUM-BINDING PROTEIN)	90	100	77.78	100	TFP	DB00831 (Trifluoperazine)	DB00831 (Trifluoperazine) DB00508 (Triflupromazine)	DB00831 (Trifluoperazine) DB00623 (Fluphenazine)	DB00831 (Trifluoperazine) DB00508 (Triflupromazine) DB00623 (Fluphenazine)
Cryptosporidium parvum Iowa II.	A3FQ56	Calmodulin.	cgd2_810.2.pdb	1rfjA (METAL BINDING PROTEIN)	87	2.07	1cll_ (CALCIUM-BINDING PROTEIN)	83.33	100	100	100	EOH	DB00898 (Ethanol)	DB00898 (Ethanol)	DB00898 (Ethanol)	DB00898 (Ethanol)
Cryptosporidium parvum Iowa II.	A3FQ99	ARF1/2 like small GTpase, putative.	cgd2_3410.2.pdb	1mr3F (SIGNALING PROTEIN)	73	2.02	1mr3F (SIGNALING PROTEIN)	100	100	76.92	100	EOH	DB00898 (Ethanol)	DB00898 (Ethanol)	DB00898 (Ethanol)	DB00898 (Ethanol)
Cryptosporidium parvum Iowa II.	A3FQG5	Serine/threonine kinase-1.	cgd5_250.2.pdb	2eu9A (TRANSFERASE)	41	1.09	1q97B (TRANSFERASE)	76.92	92.86	83.33	100	ADN			DB00194 (Vidarabine)	DB00242 (Cladribine) DB00194 (Vidarabine)
Mycobacterium tuberculosis	A4KHB1	Adenosylmethionine-8-amino-7-oxononanoate aminotransferase bioA.	15608706.2.pdb	1s0aA (TRANSFERASE)	50	1.7	1qj3B (AMINOTRANSFERASE)	86.67	100	85.71	100	KAP				DB00302 (Tranexamic Acid)
Plasmodium vivax.	A5K0P0	Glucose-6-phosphate isomerase (EC 5.3.1.9).	Pv084735.2.pdb	1t10A (ISOMERASE)	39	1.41	1iatA (ISOMERASE)	100	100	87.5	100	SUL			DB00653 (Magnesium Sulfate)	DB00653 (Magnesium Sulfate)
Plasmodium vivax.	A5K0Q8	Calmodulin, putative.	Pv084825.3.pdb	2f3yA (METAL BINDING PROTEIN)	90	2.12	1lin_ (CALCIUM-BINDING PROTEIN)	100	100	86.21	100	TFP	DB00831 (Trifluoperazine)	DB00831 (Trifluoperazine) DB00508 (Triflupromazine)	DB00831 (Trifluoperazine) DB00623 (Fluphenazine)	DB00831 (Trifluoperazine) DB00508 (Triflupromazine) DB00623 (Fluphenazine)
Plasmodium vivax.	A5K0Q8	Calmodulin, putative.	Pv084825.3.pdb	2f3yA (METAL BINDING PROTEIN)	90	2.12	1l7zA (METAL BINDING PROTEIN/PROTEIN BINDING)	100	84.62	100	100	MYR				DB00313 (Valproic Acid) DB00548 (Azelaic Acid)
Plasmodium vivax.	A5K0Q8	Calmodulin, putative.	Pv084825.2.pdb	1r2uA (METAL BINDING PROTEIN)	63	1.44	1lxfC (METAL BINDING PROTEIN, PROTEIN BINDING)	100	100	75	100	BEP	DB01244 (Bepridil)	DB01244 (Bepridil)	DB01244 (Bepridil)	DB01244 (Bepridil)
Plasmodium vivax.	A5K0Q8	Calmodulin, putative.	Pv084825.3.pdb	2f3yA (METAL BINDING PROTEIN)	90	2.12	1dtlA (STRUCTURAL PROTEIN)	78.95	100	83.33	100	BEP	DB01244 (Bepridil)	DB01244 (Bepridil)	DB01244 (Bepridil)	DB01244 (Bepridil)
Plasmodium vivax.	A5K274	Hexokinase, putative.	Pv114315.3.pdb	1v4sA (TRANSFERASE)	28	1.19	1ig8A (TRANSFERASE)	83.33	100	80	100	SUL			DB00653 (Magnesium Sulfate)	DB00653 (Magnesium Sulfate)
Plasmodium vivax.	A5K2B0	Acetyl-CoA synthetase, putative.	Pv114495.1.pdb	1pg4A (LIGASE)	47	1.47	1pg4B (LIGASE)	100	100	90.48	100	PRX		DB00194 (Vidarabine)		DB01073 (Fludarabine) DB00194 (Vidarabine)
Plasmodium vivax.	A5K2S0	Nucleoside diphosphate kinase (EC 2.7.4.6).	Pv115285.3.pdb	1npk_ (PHOSPHOTRANSFERASE(PO4 AS ACCEPTOR))	57	1.78	1pg4B (LIGASE)	100	100	83.33	100	CMP				DB01073 (Fludarabine)
Plasmodium vivax.	A5K2Y7	Phosphoribosylpyrophosphate synthetase, putative.	Pv116620.1.pdb	1dkuA (TRANSFERASE)	51	1.41	1dkuA (TRANSFERASE)	100	100	75	100	ABM				DB01073 (Fludarabine)
Plasmodium vivax.	A5K3L2	6-phosphogluconate dehydrogenase, decarboxylating (EC 1.1.1.44).	Pv117745.1.pdb	2pgd_ (OXIDOREDUCTASE (CHOH(D)-NADP+(A)))	46	1.66	1dkuA (TRANSFERASE)	100	100	80	100	2AM				DB01073 (Fludarabine)
Plasmodium vivax.	A5K3P1	Calcineurin B subunit isoform 1, putative.	Pv117895.2.pdb	1auiB (HYDROLASE)	51	1.63	1tcoB (COMPLEX (HYDROLASE/ISOMERASE))	100	100	100	100	FK5	DB00864 (Tacrolimus)	DB00864 (Tacrolimus)	DB00864 (Tacrolimus)	DB00864 (Tacrolimus) DB00337 (Pimecrolimus)
Plasmodium vivax.	A5K3V7	Serine/threonine kinase-1, putative.	Pv118220.2.pdb	2eu9A (TRANSFERASE)	40	1.11	1q97B (TRANSFERASE)	76.92	92.86	83.33	100	ADN			DB00194 (Vidarabine)	DB00242 (Cladribine) DB00194 (Vidarabine)
Plasmodium vivax.	A5K408	Delta-aminolevulinic acid dehydratase (EC 4.2.1.24).	Pv118480.2.pdb	2c1hA (LYASE)	41	1.32	1l6sB (LYASE)	94.12	100	88.89	100	DSB				DB00548 (Azelaic Acid)
Plasmodium vivax.	A5K408	Delta-aminolevulinic acid dehydratase (EC 4.2.1.24).	Pv118480.3.pdb	1h7nA (DEHYDRATASE)	28	1.21	1eb3A (DEHYDRATASE)	100	100	88.89	100	DSB				DB00548 (Azelaic Acid)
Plasmodium vivax.	A5K408	Delta-aminolevulinic acid dehydratase (EC 4.2.1.24).	Pv118480.3.pdb	1h7nA (DEHYDRATASE)	28	1.21	1gjpA (DEHYDRATASE)	94.74	100	76.19	100	4OX				DB00548 (Azelaic Acid)
Plasmodium vivax.	A5K408	Delta-aminolevulinic acid dehydratase (EC 4.2.1.24).	Pv118480.3.pdb	1h7nA (DEHYDRATASE)	28	1.21	1h7rA (DEHYDRATASE)	100	100	100	100	SHU				DB00548 (Azelaic Acid)
Plasmodium vivax.	A5K408	Delta-aminolevulinic acid dehydratase (EC 4.2.1.24).	Pv118480.2.pdb	2c1hA (LYASE)	41	1.32	1l6yA (LYASE)	80	100	85.71	100	4OX				DB00548 (Azelaic Acid)
Plasmodium vivax.	A5K408	Delta-aminolevulinic acid dehydratase (EC 4.2.1.24).	Pv118480.3.pdb	1h7nA (DEHYDRATASE)	28	1.21	1b4kB (LYASE)	92.86	100	100	100	LEA			DB00510 (Divalproex sodium) DB00313 (Valproic Acid)	DB00510 (Divalproex sodium) DB00313 (Valproic Acid)
Plasmodium vivax.	A5K408	Delta-aminolevulinic acid dehydratase (EC 4.2.1.24).	Pv118480.3.pdb	1h7nA (DEHYDRATASE)	28	1.21	1h7oA (DEHYDRATASE)	100	100	92.31	100	DAV				DB00855 (Aminolevulinic acid)
Plasmodium vivax.	A5K408	Delta-aminolevulinic acid dehydratase (EC 4.2.1.24).	Pv118480.2.pdb	2c1hA (LYASE)	41	1.32	1b4eA (LYASE)	100	100	92.31	100	LEA			DB00510 (Divalproex sodium) DB00313 (Valproic Acid)	DB00510 (Divalproex sodium) DB00313 (Valproic Acid)
Plasmodium vivax.	A5K411	Triosephosphate isomerase (EC 5.3.1.1).	Pv118495.2.pdb	1o5xA (ISOMERASE)	83	2.17	1tpfB (ISOMERASE(INTRAMOLECULAR)	85.71	100	100	100	DMS	DB01093 (Dimethyl sulfoxide)	DB01093 (Dimethyl sulfoxide)	DB01093 (Dimethyl sulfoxide)	DB01093 (Dimethyl sulfoxide)
Plasmodium vivax.	A5K620	Bifunctional dihydrofolate reductase-thymidylate synthase 1, putative.	Pv089950.3.pdb	1qzfA (OXIDOREDUCTASE, TRANSFERASE)	43	1.21	1dduB (METHYLTRANSFERASE)	100	100	92.31	100	DDU			DB00322 (Floxuridine) DB00432 (Trifluridine)	DB00322 (Floxuridine) DB00432 (Trifluridine)
Plasmodium vivax.	A5K620	Bifunctional dihydrofolate reductase-thymidylate synthase 1, putative.	Pv089950.4.pdb	1j3kC (OXIDOREDUCTASE, TRANSFERASE)	88	1.67	2tdd_ (TRANSFERASE (METHYLTRANFERASE))	93.33	100	100	100	UFP		DB00322 (Floxuridine)		DB00322 (Floxuridine)
Plasmodium vivax.	A5K620	Bifunctional dihydrofolate reductase-thymidylate synthase 1, putative.	Pv089950.4.pdb	1j3kC (OXIDOREDUCTASE, TRANSFERASE)	88	1.67	1dduA (METHYLTRANSFERASE)	100	100	100	100	DDU			DB00322 (Floxuridine) DB00432 (Trifluridine)	DB00322 (Floxuridine) DB00432 (Trifluridine)
Plasmodium vivax.	A5K620	Bifunctional dihydrofolate reductase-thymidylate synthase 1, putative.	Pv089950.4.pdb	1j3kC (OXIDOREDUCTASE, TRANSFERASE)	88	1.67	1tduA (METHYLTRANSFERASE)	94.12	100	100	100	DUR			DB00322 (Floxuridine) DB00432 (Trifluridine)	DB00322 (Floxuridine) DB00432 (Trifluridine)
Plasmodium vivax.	A5K620	Bifunctional dihydrofolate reductase-thymidylate synthase 1, putative.	Pv089950.4.pdb	1j3kC (OXIDOREDUCTASE, TRANSFERASE)	88	1.67	1ju6D (TRANSFERASE)	100	88.24	100	100	LYA	DB00642 (Pemetrexed)	DB00642 (Pemetrexed)	DB00642 (Pemetrexed)	DB00642 (Pemetrexed)
Plasmodium vivax.	A5K620	Bifunctional dihydrofolate reductase-thymidylate synthase 1, putative.	Pv089950.4.pdb	1j3kC (OXIDOREDUCTASE, TRANSFERASE)	88	1.67	1rtsA (METHYLTRANSFERASE)	100	100	96.3	100	D16	DB00293 (Raltitrexed)	DB00293 (Raltitrexed)	DB00293 (Raltitrexed)	DB00293 (Raltitrexed)
Plasmodium vivax.	A5K620	Bifunctional dihydrofolate reductase-thymidylate synthase 1, putative.	Pv089950.4.pdb	1j3kC (OXIDOREDUCTASE, TRANSFERASE)	88	1.67	1jtuB (TRANSFERASE)	75	84.21	100	100	LYB				DB00642 (Pemetrexed)
Plasmodium vivax.	A5K620	Bifunctional dihydrofolate reductase-thymidylate synthase 1, putative.	Pv089950.4.pdb	1j3kC (OXIDOREDUCTASE, TRANSFERASE)	88	1.67	1jutB (TRANSFERASE)	92.31	86.67	100	100	LYD				DB00642 (Pemetrexed)
Plasmodium vivax.	A5K620	Bifunctional dihydrofolate reductase-thymidylate synthase 1, putative.	Pv089950.3.pdb	1qzfA (OXIDOREDUCTASE, TRANSFERASE)	43	1.21	1f4fB (TRANSFERASE)	100	100	100	100	SUL			DB00653 (Magnesium Sulfate)	DB00653 (Magnesium Sulfate)
Plasmodium vivax.	A5K620	Bifunctional dihydrofolate reductase-thymidylate synthase 1, putative.	Pv089950.4.pdb	1j3kC (OXIDOREDUCTASE, TRANSFERASE)	88	1.67	1f4fA (TRANSFERASE)	100	100	100	100	SUL			DB00653 (Magnesium Sulfate)	DB00653 (Magnesium Sulfate)
Plasmodium vivax.	A5K6S2	Inosine-5'-monophosphate dehydrogenase, putative.	Pv099455.1.pdb	1jr1A (OXIDOREDUCTASE)	42	1.11	1jr1B (OXIDOREDUCTASE)	100	100	100	100	MOA	DB01024 (Mycophenolic acid)	DB01024 (Mycophenolic acid)	DB00688 (Mycophenolate mofetil) DB01024 (Mycophenolic acid)	DB00688 (Mycophenolate mofetil) DB01024 (Mycophenolic acid)
Plasmodium vivax.	A5K6X2	Histone deacetylase, putative.	Pv099700.2.pdb	1t64A (HYDROLASE)	40	1.41	1c3sA (LYASE)	83.33	80	90.91	100	SHH	DB02546 (Vorinostat)	DB02546 (Vorinostat)	DB02546 (Vorinostat)	DB02546 (Vorinostat)
Plasmodium vivax.	A5K7I5	Enolase (EC 4.2.1.11).	Pv095015.2.pdb	2akzA (LYASE)	70	1.96	2oneA (LYASE)	100	100	100	100	LI	DB01356 (Lithium)	DB01356 (Lithium)	DB01356 (Lithium)	DB01356 (Lithium)
Plasmodium vivax.	A5K9E9	Adenosylhomocysteinase (EC 3.3.1.1).	Pv080200.1.pdb	1v8bA (HYDROLASE)	89	2.13	1ky5D (HYDROLASE)	87.5	100	95.65	100	ADY				DB00194 (Vidarabine)
Plasmodium vivax.	A5K9E9	Adenosylhomocysteinase (EC 3.3.1.1).	Pv080200.1.pdb	1v8bA (HYDROLASE)	89	2.13	1d4fB (HYDROLASE)	91.3	100	95.65	100	ADN			DB00194 (Vidarabine)	DB00242 (Cladribine) DB00194 (Vidarabine)
Plasmodium vivax.	A5KAZ4	Histone deacetylase, putative.	Pv097890.1.pdb	1c3pA (LYASE)	19	1.04	1c3sA (LYASE)	100	100	85.71	92.86	SHH	DB02546 (Vorinostat)	DB02546 (Vorinostat)	DB02546 (Vorinostat)	DB02546 (Vorinostat)
Plasmodium vivax.	A5KBJ3	Aspartate aminotransferase, mitochondrial, putative.	Pv003655.3.pdb	7aatA (TRANSFERASE(AMINOTRANSFERASE))	31	1.48	2ay2B (AMINOTRANSFERASE)	100	100	76.92	100	CXP				DB00548 (Azelaic Acid)
Plasmodium vivax.	A5KBJ3	Aspartate aminotransferase, mitochondrial, putative.	Pv003655.2.pdb	1ars_ (TRANSFERASE(AMINOTRANSFERASE))	29	1.49	1c3sA (LYASE)	100	100	84.62	100	CXP				DB00548 (Azelaic Acid)
Plasmodium vivax.	A5KCF1	ADP-ribosylation factor, putative.	Pv001905.2.pdb	1z6xA (TRANSPORT PROTEIN)	70	2	1mr3F (SIGNALING PROTEIN)	75	100	80	100	EOH	DB00898 (Ethanol)	DB00898 (Ethanol)	DB00898 (Ethanol)	DB00898 (Ethanol)
Plasmodium vivax.	A5KCI8	Serine/threonine protein phosphatase (EC 3.1.3.16).	Pv093605.1.pdb	1auiA (HYDROLASE)	53	1.35	1tcoA (COMPLEX (HYDROLASE/ISOMERASE))	100	100	100	100	FK5	DB00864 (Tacrolimus)	DB00864 (Tacrolimus)	DB00864 (Tacrolimus)	DB00864 (Tacrolimus) DB00337 (Pimecrolimus)
Plasmodium vivax.	A5KE97	cAMP-dependent protein kinase catalytic subunit, putative.	Pv086975.2.pdb	1rdqE (TRANSFERASE/TRANSFERASE INHIBITOR)	47	1.66	1fmoE (COMPLEX (PHOSPHOTRANSFERASE/INHIBITOR))	100	100	83.33	100	ADN			DB00194 (Vidarabine)	DB00242 (Cladribine) DB00194 (Vidarabine)
Mycobacterium tuberculosis	A5U166	Enolase (EC 4.2.1.11) (2-phosphoglycerate dehydratase) (2-phospho-D-glycerate hydro-lyase).	15608163.2.pdb	2fymA (LYASE)	56	1.8	2oneA (LYASE)	100	85.71	100	100	LI	DB01356 (Lithium)	DB01356 (Lithium)	DB01356 (Lithium)	DB01356 (Lithium)
Mycobacterium tuberculosis	A5U2B7	Riboflavin biosynthesis protein ribBA [Includes: 3,4-dihydroxy-2-butanone 4-phosphate synthase (EC 4.1.99.12) (DHBP synthase); GTPcyclohydrolase-2 (EC 3.5.4.25) (GTP cyclohydrolase II)].	15608553.1.pdb	1g57A (ISOMERASE)	46	1.18	1k4oA (ISOMERASE)	80	100	88.89	100	SUL			DB00653 (Magnesium Sulfate)	DB00653 (Magnesium Sulfate)
Mycobacterium tuberculosis	A5U2E0	Triosephosphate isomerase (EC 5.3.1.1) (TIM) (Triose-phosphateisomerase).	15608576.1.pdb	1mo0A (ISOMERASE)	40	1.56	1tpfA (ISOMERASE(INTRAMOLECULAR)	100	100	83.33	100	DMS	DB01093 (Dimethyl sulfoxide)	DB01093 (Dimethyl sulfoxide)	DB01093 (Dimethyl sulfoxide)	DB01093 (Dimethyl sulfoxide)
Mycobacterium tuberculosis	A5U2W7	Indole-3-glycerol phosphate synthase (EC 4.1.1.48) (IGPS).	15608749.1.pdb	1i4nA (LYASE)	28	1.25	1i4nA (LYASE)	100	100	77.78	100	SUL			DB00653 (Magnesium Sulfate)	DB00653 (Magnesium Sulfate)
Mycobacterium tuberculosis	A5U3L7	Urease subunit alpha (EC 3.5.1.5) (Urea amidohydrolase subunit alpha).	15608987.3.pdb	4ubpC (HYDROLASE)	57	1.68	4ubpC (HYDROLASE)	100	100	100	100	HAE	DB00551 (Acetohydroxamic Acid)	DB00551 (Acetohydroxamic Acid)	DB00551 (Acetohydroxamic Acid)	DB00551 (Acetohydroxamic Acid)
Mycobacterium tuberculosis	A5U6B7	Thymidylate synthase (EC 2.1.1.45) (TS) (TSase).	15609901.2.pdb	2g8mA (TRANSFERASE)	66	1.89	2kceA (METHYLTRANSFERASE)	95.45	100	91.67	100	D16	DB00293 (Raltitrexed)	DB00293 (Raltitrexed)	DB00293 (Raltitrexed)	DB00293 (Raltitrexed)
Mycobacterium tuberculosis	A5U6B7	Thymidylate synthase (EC 2.1.1.45) (TS) (TSase).	15609901.1.pdb	1tis_ (TRANSFERASE(METHYLTRANSFERASE))	48	1.48	4ubpC (HYDROLASE)	100	100	90	100	UFP		DB00322 (Floxuridine)		DB00322 (Floxuridine)
Mycobacterium tuberculosis	A5U6B7	Thymidylate synthase (EC 2.1.1.45) (TS) (TSase).	15609901.2.pdb	2g8mA (TRANSFERASE)	66	1.89	1dduB (METHYLTRANSFERASE)	100	100	76.92	100	DDU			DB00322 (Floxuridine) DB00432 (Trifluridine)	DB00322 (Floxuridine) DB00432 (Trifluridine)
Mycobacterium tuberculosis	A5U6B7	Thymidylate synthase (EC 2.1.1.45) (TS) (TSase).	15609901.2.pdb	2g8mA (TRANSFERASE)	66	1.89	1tlsB (METHYLTRANSFERASE)	94.74	100	80.95	100	UFP		DB00322 (Floxuridine)		DB00322 (Floxuridine)
Mycobacterium tuberculosis	A5U6B7	Thymidylate synthase (EC 2.1.1.45) (TS) (TSase).	15609901.2.pdb	2g8mA (TRANSFERASE)	66	1.89	1jutB (TRANSFERASE)	93.33	100	92.86	100	LYD				DB00642 (Pemetrexed)
Mycobacterium tuberculosis	A5U6B7	Thymidylate synthase (EC 2.1.1.45) (TS) (TSase).	15609901.2.pdb	2g8mA (TRANSFERASE)	66	1.89	1axwB (METHYLTRANSFERASE)	95.45	100	86.96	100	MTX	DB00563 (Methotrexate)	DB00563 (Methotrexate)	DB00563 (Methotrexate)	DB00563 (Methotrexate)
Mycobacterium tuberculosis	A5U6B7	Thymidylate synthase (EC 2.1.1.45) (TS) (TSase).	15609901.2.pdb	2g8mA (TRANSFERASE)	66	1.89	1jtuA (TRANSFERASE)	94.74	100	89.47	100	LYB				DB00642 (Pemetrexed)
Mycobacterium tuberculosis	A5U6B7	Thymidylate synthase (EC 2.1.1.45) (TS) (TSase).	15609901.1.pdb	1tis_ (TRANSFERASE(METHYLTRANSFERASE))	48	1.48	4ubpC (HYDROLASE)	100	100	81.25	93.75	DUR			DB00322 (Floxuridine) DB00432 (Trifluridine)	DB00322 (Floxuridine) DB00432 (Trifluridine)
Mycobacterium tuberculosis	A5U6B7	Thymidylate synthase (EC 2.1.1.45) (TS) (TSase).	15609901.2.pdb	2g8mA (TRANSFERASE)	66	1.89	1tduA (METHYLTRANSFERASE)	94.12	100	82.35	100	DUR			DB00322 (Floxuridine) DB00432 (Trifluridine)	DB00322 (Floxuridine) DB00432 (Trifluridine)
Mycobacterium tuberculosis	A5U6B7	Thymidylate synthase (EC 2.1.1.45) (TS) (TSase).	15609901.1.pdb	1tis_ (TRANSFERASE(METHYLTRANSFERASE))	48	1.48	4ubpC (HYDROLASE)	100	100	83.33	91.67	LYD				DB00642 (Pemetrexed)
Mycobacterium tuberculosis	A5U6B7	Thymidylate synthase (EC 2.1.1.45) (TS) (TSase).	15609901.1.pdb	1tis_ (TRANSFERASE(METHYLTRANSFERASE))	48	1.48	4ubpC (HYDROLASE)	100	100	87.5	100	SUL			DB00653 (Magnesium Sulfate)	DB00653 (Magnesium Sulfate)
Mycobacterium tuberculosis	A5U6B7	Thymidylate synthase (EC 2.1.1.45) (TS) (TSase).	15609901.1.pdb	1tis_ (TRANSFERASE(METHYLTRANSFERASE))	48	1.48	4ubpC (HYDROLASE)	100	100	84.62	100	DDU			DB00322 (Floxuridine) DB00432 (Trifluridine)	DB00322 (Floxuridine) DB00432 (Trifluridine)
Mycobacterium tuberculosis	A5U7S2	Adenosylhomocysteinase (EC 3.3.1.1) (S-adenosyl-L-homocysteinehydrolase) (AdoHcyase).	15610384.1.pdb	1li4A (HYDROLASE)	60	1.43	1ky5D (HYDROLASE)	100	100	91.3	100	ADY				DB00194 (Vidarabine)
Mycobacterium tuberculosis	A5U7S2	Adenosylhomocysteinase (EC 3.3.1.1) (S-adenosyl-L-homocysteinehydrolase) (AdoHcyase).	15610384.1.pdb	1li4A (HYDROLASE)	60	1.43	1d4fD (HYDROLASE)	100	100	91.3	100	ADN			DB00194 (Vidarabine)	DB00242 (Cladribine) DB00194 (Vidarabine)
Mycobacterium tuberculosis	A5U7S2	Adenosylhomocysteinase (EC 3.3.1.1) (S-adenosyl-L-homocysteinehydrolase) (AdoHcyase).	15610384.2.pdb	1v8bA (HYDROLASE)	57	1.73	1d4fB (HYDROLASE)	91.3	100	86.96	100	ADN			DB00194 (Vidarabine)	DB00242 (Cladribine) DB00194 (Vidarabine)
Mycobacterium tuberculosis	A5U7Y3	Uracil phosphoribosyltransferase (EC 2.4.2.9) (UMP pyrophosphorylase)(UPRTase).	15610445.2.pdb	1o5oA (TRANSFERASE)	49	1.74	1upfD (TRANSFERASE)	80	100	88.89	100	URF	DB00544 (Fluorouracil)	DB00544 (Fluorouracil)	DB00544 (Fluorouracil)	DB00544 (Fluorouracil)

Organism	UniProt Id	Target description	3D model id	PDB Template	Model sequence identity	ModPipe Quality Score	PDB Expansion id	Binding site expansion sequence identity	Binding site expansion structure identity	Binding site sequence identity	Binding site structure identity	PDB Ligand id	Drugbank (Exact)	Drugbank (supra-structure)	Drugbank (sub-structure)	Drugbank (similar)
Mycobacterium tuberculosis	A5U866	Cyclopropane-fatty-acyl-phospholipid synthase 1 (EC 2.1.1.79)(Cyclopropane fatty acid synthase) (CFA synthase) (Cyclopropanemycolic acid synthase 1).	15610528.1.pdb	1kpgA (TRANSFERASE)	100	2.27	1kpgD (TRANSFERASE)	100	100	100	100	16A				DB01245 (Decamethonium)
Mycobacterium tuberculosis	A5U8S7	Pantothenate synthetase (EC 6.3.2.1) (PS) (Pantoate--beta-alanineligase) (Pantoate-activating enzyme).	15610738.1.pdb	2a84A (LIGASE)	100	2.19	1n2hB (LIGASE)	96.3	93.1	100	100	PAJ				DB01073 (Fludarabine)
Mycobacterium tuberculosis	A5U8S7	Pantothenate synthetase (EC 6.3.2.1) (PS) (Pantoate--beta-alanineligase) (Pantoate-activating enzyme).	15610738.1.pdb	2a84A (LIGASE)	100	2.19	1n2gB (LIGASE)	100	100	100	100	EOH	DB00898 (Ethanol)	DB00898 (Ethanol)	DB00898 (Ethanol)	DB00898 (Ethanol)
Plasmodium falciparum.	A7UD81	Dihydrofolate reductase-thymidylate synthase.	PFD0830w.1.pdb	1qzfA (OXIDOREDUCTASE, TRANSFERASE)	42	1.29	1dduB (METHYLTRANSFERASE)	100	100	92.31	100	DDU			DB00322 (Floxuridine) DB00432 (Trifluridine)	DB00322 (Floxuridine) DB00432 (Trifluridine)
Plasmodium falciparum.	A7UD81	Dihydrofolate reductase-thymidylate synthase.	PFD0830w.1.pdb	1qzfA (OXIDOREDUCTASE, TRANSFERASE)	42	1.29	2tdd_ (TRANSFERASE (METHYLTRANFERASE))	93.33	100	95.45	100	UFP		DB00322 (Floxuridine)		DB00322 (Floxuridine)
Plasmodium falciparum.	A7UD81	Dihydrofolate reductase-thymidylate synthase.	PFD0830w.1.pdb	1qzfA (OXIDOREDUCTASE, TRANSFERASE)	42	1.29	1jutB (TRANSFERASE)	86.67	100	92.86	100	LYD				DB00642 (Pemetrexed)
Plasmodium falciparum.	A7UD81	Dihydrofolate reductase-thymidylate synthase.	PFD0830w.1.pdb	1qzfA (OXIDOREDUCTASE, TRANSFERASE)	42	1.29	1tduA (METHYLTRANSFERASE)	94.12	100	88.24	100	DUR			DB00322 (Floxuridine) DB00432 (Trifluridine)	DB00322 (Floxuridine) DB00432 (Trifluridine)
Plasmodium falciparum.	A7UD81	Dihydrofolate reductase-thymidylate synthase.	PFD0830w.1.pdb	1qzfA (OXIDOREDUCTASE, TRANSFERASE)	42	1.29	1ju6D (TRANSFERASE)	94.12	100	84.21	100	LYA	DB00642 (Pemetrexed)	DB00642 (Pemetrexed)	DB00642 (Pemetrexed)	DB00642 (Pemetrexed)
Plasmodium falciparum.	A7UD81	Dihydrofolate reductase-thymidylate synthase.	PFD0830w.1.pdb	1qzfA (OXIDOREDUCTASE, TRANSFERASE)	42	1.29	1f4fB (TRANSFERASE)	100	100	100	100	SUL			DB00653 (Magnesium Sulfate)	DB00653 (Magnesium Sulfate)
Plasmodium vivax.	O02604	Bifunctional dihydrofolate reductase-thymidylate synthase (DHFR-TS)[Includes: Dihydrofolate reductase (EC 1.5.1.3); Thymidylate synthase(EC 2.1.1.45)].	Pv089950.5.pdb	1f28A (TRANSFERASE)	57	1.31	1dduB (METHYLTRANSFERASE)	100	100	100	100	DDU			DB00322 (Floxuridine) DB00432 (Trifluridine)	DB00322 (Floxuridine) DB00432 (Trifluridine)
Plasmodium vivax.	O02604	Bifunctional dihydrofolate reductase-thymidylate synthase (DHFR-TS)[Includes: Dihydrofolate reductase (EC 1.5.1.3); Thymidylate synthase(EC 2.1.1.45)].	Pv089950.5.pdb	1f28A (TRANSFERASE)	57	1.31	1ju6D (TRANSFERASE)	94.12	100	84.21	100	LYA	DB00642 (Pemetrexed)	DB00642 (Pemetrexed)	DB00642 (Pemetrexed)	DB00642 (Pemetrexed)
Plasmodium vivax.	O02604	Bifunctional dihydrofolate reductase-thymidylate synthase (DHFR-TS)[Includes: Dihydrofolate reductase (EC 1.5.1.3); Thymidylate synthase(EC 2.1.1.45)].	Pv089950.5.pdb	1f28A (TRANSFERASE)	57	1.31	2tdd_ (TRANSFERASE (METHYLTRANFERASE))	93.33	100	95.45	100	UFP		DB00322 (Floxuridine)		DB00322 (Floxuridine)
Plasmodium vivax.	O02604	Bifunctional dihydrofolate reductase-thymidylate synthase (DHFR-TS)[Includes: Dihydrofolate reductase (EC 1.5.1.3); Thymidylate synthase(EC 2.1.1.45)].	Pv089950.5.pdb	1f28A (TRANSFERASE)	57	1.31	1f4fA (TRANSFERASE)	100	100	100	100	SUL			DB00653 (Magnesium Sulfate)	DB00653 (Magnesium Sulfate)
Plasmodium vivax.	O02604	Bifunctional dihydrofolate reductase-thymidylate synthase (DHFR-TS)[Includes: Dihydrofolate reductase (EC 1.5.1.3); Thymidylate synthase(EC 2.1.1.45)].	Pv089950.5.pdb	1f28A (TRANSFERASE)	57	1.31	1tduA (METHYLTRANSFERASE)	94.12	100	94.12	100	DUR			DB00322 (Floxuridine) DB00432 (Trifluridine)	DB00322 (Floxuridine) DB00432 (Trifluridine)
Plasmodium vivax.	O02604	Bifunctional dihydrofolate reductase-thymidylate synthase (DHFR-TS)[Includes: Dihydrofolate reductase (EC 1.5.1.3); Thymidylate synthase(EC 2.1.1.45)].	Pv089950.5.pdb	1f28A (TRANSFERASE)	57	1.31	1jutB (TRANSFERASE)	80	100	85.71	100	LYD				DB00642 (Pemetrexed)
Plasmodium vivax.	O02604	Bifunctional dihydrofolate reductase-thymidylate synthase (DHFR-TS)[Includes: Dihydrofolate reductase (EC 1.5.1.3); Thymidylate synthase(EC 2.1.1.45)].	Pv089950.2.pdb	2bl9A (OXIDOREDUCTASE)	98	1.46	1j3jB (OXIDOREDUCTASE, TRANSFERASE)	94.12	100	100	100	CP6	DB00205 (Pyrimethamine)	DB00205 (Pyrimethamine)	DB00205 (Pyrimethamine)	DB00205 (Pyrimethamine)
Mycobacterium tuberculosis.	O05891	Thymidylate kinase (EC 2.7.4.9) (Thymidine monophosphate kinase) (dTMPkinase) (TMPK).	15610383.1.pdb	1gtvA (TRANSFERASE)	100	2.22	1g3uA (TRANSFERASE)	90	100	100	100	SUL			DB00653 (Magnesium Sulfate)	DB00653 (Magnesium Sulfate)
Toxoplasma gondii.	O15694	SAG1-related sequence 3.	42.m00008.1.pdb	1v8bA (HYDROLASE)	60	1.64	1ky5D (HYDROLASE)	87.5	100	86.96	100	ADY				DB00194 (Vidarabine)
Toxoplasma gondii.	O15694	SAG1-related sequence 3.	42.m00008.1.pdb	1v8bA (HYDROLASE)	60	1.64	1d4fB (HYDROLASE)	91.3	100	86.96	100	ADN			DB00194 (Vidarabine)	DB00242 (Cladribine) DB00194 (Vidarabine)
Plasmodium falciparum.	O15906	CAMP-dependent protein kinase catalytic subunit (CAMP-dependentprotein kinase).	PFI1685w.2.pdb	1rdqE (TRANSFERASE/TRANSFERASE INHIBITOR)	46	1.57	1fmoE (COMPLEX (PHOSPHOTRANSFERASE/INHIBITOR))	100	100	83.33	100	ADN			DB00194 (Vidarabine)	DB00242 (Cladribine) DB00194 (Vidarabine)
Mycobacterium leprae.	O32954	Probable branched-chain-amino-acid aminotransferase (EC 2.6.1.42)(BCAT).	13092941.2.pdb	2coiA (TRANSFERASE)	38	1.5	1i1mB (TRANSFERASE)	81.82	84.62	81.82	100	4MV				DB00510 (Divalproex sodium) DB00313 (Valproic Acid)
Mycobacterium leprae.	O33066	Citrate synthase (EC 2.3.3.1).	13093696.2.pdb	1o7xA (SYNTHASE)	34	1.48	1nxgB (LYASE)	87.5	88.89	80	100	SUL			DB00653 (Magnesium Sulfate)	DB00653 (Magnesium Sulfate)
Mycobacterium leprae.	O33066	Citrate synthase (EC 2.3.3.1).	13093696.1.pdb	1k3pA (LYASE)	52	1.74	1nxgB (LYASE)	100	100	83.33	100	SUL			DB00653 (Magnesium Sulfate)	DB00653 (Magnesium Sulfate)
Plasmodium vivax.	O60989	Plasmepsin (Aspartic protease PM4, putative) (Aspartic protease PM4).	Pv086040.1.pdb	1miqA (HYDROLASE)	100	2.09	3pep_ (HYDROLASE (ACID PROTEINASE))	83.33	100	100	100	EOH	DB00898 (Ethanol)	DB00898 (Ethanol)	DB00898 (Ethanol)	DB00898 (Ethanol)
Mycobacterium leprae.	O69524	Pantothenate synthetase (EC 6.3.2.1) (PS) (Pantoate--beta-alanineligase) (Pantoate-activating enzyme).	13092574.1.pdb	2a84A (LIGASE)	84	2.01	1n2hB (LIGASE)	96.3	93.1	100	100	PAJ				DB01073 (Fludarabine)
Mycobacterium leprae.	O69524	Pantothenate synthetase (EC 6.3.2.1) (PS) (Pantoate--beta-alanineligase) (Pantoate-activating enzyme).	13092574.1.pdb	2a84A (LIGASE)	84	2.01	1n2gB (LIGASE)	100	100	84.93	100	EOH	DB00898 (Ethanol)	DB00898 (Ethanol)	DB00898 (Ethanol)	DB00898 (Ethanol)
Mycobacterium tuberculosis.	O69635	Acetyl-coenzyme A synthetase (EC 6.2.1.1) (Acetate--CoA ligase) (Acyl-activating enzyme).	15610803.1.pdb	1pg4A (LIGASE)	48	1.63	1pg4B (LIGASE)	100	100	90.48	100	PRX		DB00194 (Vidarabine)		DB01073 (Fludarabine) DB00194 (Vidarabine)
Trypanosoma cruzi.	O76240	S-adenosylmethionine decarboxylase proenzyme (EC 4.1.1.50) (AdoMetDC)(SamDC) [Contains: S-adenosylmethionine decarboxylase alpha chain; S-adenosylmethionine decarboxylase beta chain].	Tc00.1047053504257.3.2.pdb	1jl0A (LYASE)	33	1.24	1i72B (LYASE)	100	80	76.92	100	MAO				DB00194 (Vidarabine)
Trypanosoma cruzi.	O76240	S-adenosylmethionine decarboxylase proenzyme (EC 4.1.1.50) (AdoMetDC)(SamDC) [Contains: S-adenosylmethionine decarboxylase alpha chain; S-adenosylmethionine decarboxylase beta chain].	Tc00.1047053504257.3.2.pdb	1jl0A (LYASE)	33	1.24	1i79B (LYASE)	100	83.33	78.57	100	MHZ				DB00194 (Vidarabine)
Plasmodium falciparum.	O77078	Beta-ketoacyl-acyl carrier protein synthase III precursor.	PFB0505c.2.pdb	1hnjA (TRANSFERASE)	35	1.41	1hzpA (TRANSFERASE)	76.19	95.45	75	100	DAO				DB00313 (Valproic Acid) DB00548 (Azelaic Acid)
Plasmodium falciparum	O96142	Aspartate aminotransferase, putative.	PFB0200c.2.pdb	1ars_ (TRANSFERASE(AMINOTRANSFERASE))	32	1.56	2ay2B (AMINOTRANSFERASE)	100	100	84.62	100	CXP				DB00548 (Azelaic Acid)
Trypanosoma brucei brucei.	P04789	Triosephosphate isomerase, glycosomal (EC 5.3.1.1) (TIM) (Triose-phosphate isomerase).	Tb11.02.3210.1.pdb	1kv5A (ISOMERASE)	99	2.24	1tpfA (ISOMERASE(INTRAMOLECULAR)	100	100	100	100	DMS	DB01093 (Dimethyl sulfoxide)	DB01093 (Dimethyl sulfoxide)	DB01093 (Dimethyl sulfoxide)	DB01093 (Dimethyl sulfoxide)
Leishmania major.	P07382	Bifunctional dihydrofolate reductase-thymidylate synthase (DHFR-TS)[Includes: Dihydrofolate reductase (EC 1.5.1.3); Thymidylate synthase(EC 2.1.1.45)].	LmjF06.0860.2.pdb	1f28A (TRANSFERASE)	58	1.31	1f4fA (TRANSFERASE)	100	100	100	100	SUL			DB00653 (Magnesium Sulfate)	DB00653 (Magnesium Sulfate)
Leishmania major.	P07382	Bifunctional dihydrofolate reductase-thymidylate synthase (DHFR-TS)[Includes: Dihydrofolate reductase (EC 1.5.1.3); Thymidylate synthase(EC 2.1.1.45)].	LmjF06.0860.2.pdb	1f28A (TRANSFERASE)	58	1.31	2tsrD (METHYLTRANSFERASE)	100	100	88.46	100	D16	DB00293 (Raltitrexed)	DB00293 (Raltitrexed)	DB00293 (Raltitrexed)	DB00293 (Raltitrexed)
Leishmania major.	P07382	Bifunctional dihydrofolate reductase-thymidylate synthase (DHFR-TS)[Includes: Dihydrofolate reductase (EC 1.5.1.3); Thymidylate synthase(EC 2.1.1.45)].	LmjF06.0860.2.pdb	1f28A (TRANSFERASE)	58	1.31	1jutB (TRANSFERASE)	80	100	85.71	100	LYD				DB00642 (Pemetrexed)
Leishmania major.	P07382	Bifunctional dihydrofolate reductase-thymidylate synthase (DHFR-TS)[Includes: Dihydrofolate reductase (EC 1.5.1.3); Thymidylate synthase(EC 2.1.1.45)].	LmjF06.0860.2.pdb	1f28A (TRANSFERASE)	58	1.31	2tdd_ (TRANSFERASE (METHYLTRANFERASE))	93.33	100	95.45	100	UFP		DB00322 (Floxuridine)		DB00322 (Floxuridine)
Leishmania major.	P07382	Bifunctional dihydrofolate reductase-thymidylate synthase (DHFR-TS)[Includes: Dihydrofolate reductase (EC 1.5.1.3); Thymidylate synthase(EC 2.1.1.45)].	LmjF06.0860.1.pdb	1qzfA (OXIDOREDUCTASE, TRANSFERASE)	46	1.39	1jtuA (TRANSFERASE)	78.95	100	78.95	100	LYB				DB00642 (Pemetrexed)
Leishmania major.	P07382	Bifunctional dihydrofolate reductase-thymidylate synthase (DHFR-TS)[Includes: Dihydrofolate reductase (EC 1.5.1.3); Thymidylate synthase(EC 2.1.1.45)].	LmjF06.0860.2.pdb	1f28A (TRANSFERASE)	58	1.31	1dduB (METHYLTRANSFERASE)	100	100	100	100	DDU			DB00322 (Floxuridine) DB00432 (Trifluridine)	DB00322 (Floxuridine) DB00432 (Trifluridine)
Leishmania major.	P07382	Bifunctional dihydrofolate reductase-thymidylate synthase (DHFR-TS)[Includes: Dihydrofolate reductase (EC 1.5.1.3); Thymidylate synthase(EC 2.1.1.45)].	LmjF06.0860.2.pdb	1f28A (TRANSFERASE)	58	1.31	1ju6D (TRANSFERASE)	94.12	100	89.47	100	LYA	DB00642 (Pemetrexed)	DB00642 (Pemetrexed)	DB00642 (Pemetrexed)	DB00642 (Pemetrexed)
Leishmania major.	P07382	Bifunctional dihydrofolate reductase-thymidylate synthase (DHFR-TS)[Includes: Dihydrofolate reductase (EC 1.5.1.3); Thymidylate synthase(EC 2.1.1.45)].	LmjF06.0860.1.pdb	1qzfA (OXIDOREDUCTASE, TRANSFERASE)	46	1.39	1f4fB (TRANSFERASE)	100	100	100	100	SUL			DB00653 (Magnesium Sulfate)	DB00653 (Magnesium Sulfate)
Leishmania major.	P07382	Bifunctional dihydrofolate reductase-thymidylate synthase (DHFR-TS)[Includes: Dihydrofolate reductase (EC 1.5.1.3); Thymidylate synthase(EC 2.1.1.45)].	LmjF06.0860.2.pdb	1f28A (TRANSFERASE)	58	1.31	1tduA (METHYLTRANSFERASE)	94.12	100	94.12	100	DUR			DB00322 (Floxuridine) DB00432 (Trifluridine)	DB00322 (Floxuridine) DB00432 (Trifluridine)
Leishmania major.	P07382	Bifunctional dihydrofolate reductase-thymidylate synthase (DHFR-TS)[Includes: Dihydrofolate reductase (EC 1.5.1.3); Thymidylate synthase(EC 2.1.1.45)].	LmjF06.0860.1.pdb	1qzfA (OXIDOREDUCTASE, TRANSFERASE)	46	1.39	1axwA (METHYLTRANSFERASE)	78.95	95	77.78	100	MTX	DB00563 (Methotrexate)	DB00563 (Methotrexate)	DB00563 (Methotrexate)	DB00563 (Methotrexate)
Mycobacterium tuberculosis.	P0A538	Purine nucleoside phosphorylase (EC 2.4.2.1) (Inosine phosphorylase)(PNP).	15610443.2.pdb	1g2oA (TRANSFERASE)	100	2.3	1ivuD (TRANSFERASE)	100	100	100	100	9PP		DB00300 (Tenofovir)		DB00300 (Tenofovir)
Mycobacterium tuberculosis.	P0A538	Purine nucleoside phosphorylase (EC 2.4.2.1) (Inosine phosphorylase)(PNP).	15610443.2.pdb	1g2oA (TRANSFERASE)	100	2.3	1a9s_ (PENTOSYLTRANSFERASE)	83.33	90	100	100	NOS			DB01280 (Nelarabine)	DB01280 (Nelarabine)
Mycobacterium tuberculosis.	P0A578	Dihydropteroate synthase 1 (EC 2.5.1.15) (DHPS 1) (Dihydropteroatepyrophosphorylase 1).	57117134.1.pdb	1aj0_ (SYNTHASE)	41	1.47	1a9s_ (PENTOSYLTRANSFERASE)	83.33	90	88.89	100	SAN	DB00259 (Sulfanilamide)	DB00259 (Sulfanilamide)	DB00259 (Sulfanilamide)	DB00259 (Sulfanilamide)
Mycobacterium leprae.	P0C0X1	Dihydropteroate synthase 1 (EC 2.5.1.15) (DHPS 1) (Dihydropteroatepyrophosphorylase 1).	13092568.1.pdb	1aj0_ (SYNTHASE)	40	1.44	1a9s_ (PENTOSYLTRANSFERASE)	83.33	90	100	100	SAN	DB00259 (Sulfanilamide)	DB00259 (Sulfanilamide)	DB00259 (Sulfanilamide)	DB00259 (Sulfanilamide)
Plasmodium falciparum	P13922	Bifunctional dihydrofolate reductase-thymidylate synthase (DHFR-TS)[Includes: Dihydrofolate reductase (EC 1.5.1.3); Thymidylate synthase(EC 2.1.1.45)].	PFD0830w.2.pdb	1j3kC (OXIDOREDUCTASE, TRANSFERASE)	100	1.82	1ju6D (TRANSFERASE)	100	88.24	100	100	LYA	DB00642 (Pemetrexed)	DB00642 (Pemetrexed)	DB00642 (Pemetrexed)	DB00642 (Pemetrexed)
Plasmodium falciparum	P13922	Bifunctional dihydrofolate reductase-thymidylate synthase (DHFR-TS)[Includes: Dihydrofolate reductase (EC 1.5.1.3); Thymidylate synthase(EC 2.1.1.45)].	PFD0830w.3.pdb	1f28A (TRANSFERASE)	59	1.26	1dduB (METHYLTRANSFERASE)	100	100	100	100	DDU			DB00322 (Floxuridine) DB00432 (Trifluridine)	DB00322 (Floxuridine) DB00432 (Trifluridine)
Plasmodium falciparum	P13922	Bifunctional dihydrofolate reductase-thymidylate synthase (DHFR-TS)[Includes: Dihydrofolate reductase (EC 1.5.1.3); Thymidylate synthase(EC 2.1.1.45)].	PFD0830w.2.pdb	1j3kC (OXIDOREDUCTASE, TRANSFERASE)	100	1.82	1rtsA (METHYLTRANSFERASE)	100	100	100	100	D16	DB00293 (Raltitrexed)	DB00293 (Raltitrexed)	DB00293 (Raltitrexed)	DB00293 (Raltitrexed)
Plasmodium falciparum	P13922	Bifunctional dihydrofolate reductase-thymidylate synthase (DHFR-TS)[Includes: Dihydrofolate reductase (EC 1.5.1.3); Thymidylate synthase(EC 2.1.1.45)].	PFD0830w.2.pdb	1j3kC (OXIDOREDUCTASE, TRANSFERASE)	100	1.82	1dduA (METHYLTRANSFERASE)	100	100	100	100	DDU			DB00322 (Floxuridine) DB00432 (Trifluridine)	DB00322 (Floxuridine) DB00432 (Trifluridine)
Plasmodium falciparum	P13922	Bifunctional dihydrofolate reductase-thymidylate synthase (DHFR-TS)[Includes: Dihydrofolate reductase (EC 1.5.1.3); Thymidylate synthase(EC 2.1.1.45)].	PFD0830w.2.pdb	1j3kC (OXIDOREDUCTASE, TRANSFERASE)	100	1.82	1jtuB (TRANSFERASE)	75	84.21	100	100	LYB				DB00642 (Pemetrexed)
Plasmodium falciparum	P13922	Bifunctional dihydrofolate reductase-thymidylate synthase (DHFR-TS)[Includes: Dihydrofolate reductase (EC 1.5.1.3); Thymidylate synthase(EC 2.1.1.45)].	PFD0830w.2.pdb	1j3kC (OXIDOREDUCTASE, TRANSFERASE)	100	1.82	2tdd_ (TRANSFERASE (METHYLTRANFERASE))	93.33	100	100	100	UFP		DB00322 (Floxuridine)		DB00322 (Floxuridine)
Plasmodium falciparum	P13922	Bifunctional dihydrofolate reductase-thymidylate synthase (DHFR-TS)[Includes: Dihydrofolate reductase (EC 1.5.1.3); Thymidylate synthase(EC 2.1.1.45)].	PFD0830w.2.pdb	1j3kC (OXIDOREDUCTASE, TRANSFERASE)	100	1.82	1f4fA (TRANSFERASE)	100	100	100	100	SUL			DB00653 (Magnesium Sulfate)	DB00653 (Magnesium Sulfate)
Plasmodium falciparum	P13922	Bifunctional dihydrofolate reductase-thymidylate synthase (DHFR-TS)[Includes: Dihydrofolate reductase (EC 1.5.1.3); Thymidylate synthase(EC 2.1.1.45)].	PFD0830w.2.pdb	1j3kC (OXIDOREDUCTASE, TRANSFERASE)	100	1.82	1jutB (TRANSFERASE)	92.31	86.67	100	100	LYD				DB00642 (Pemetrexed)
Plasmodium falciparum	P13922	Bifunctional dihydrofolate reductase-thymidylate synthase (DHFR-TS)[Includes: Dihydrofolate reductase (EC 1.5.1.3); Thymidylate synthase(EC 2.1.1.45)].	PFD0830w.2.pdb	1j3kC (OXIDOREDUCTASE, TRANSFERASE)	100	1.82	1tduA (METHYLTRANSFERASE)	94.12	100	100	100	DUR			DB00322 (Floxuridine) DB00432 (Trifluridine)	DB00322 (Floxuridine) DB00432 (Trifluridine)
Trypanosoma cruzi.	P18061	Calmodulin (CaM).	Tc00.1047053506389.7.2.pdb	2f3yA (METAL BINDING PROTEIN)	90	2.14	1dtlA (STRUCTURAL PROTEIN)	78.95	100	77.78	83.33	BEP	DB01244 (Bepridil)	DB01244 (Bepridil)	DB01244 (Bepridil)	DB01244 (Bepridil)
Mycobacterium leprae.	P36429	Aspartyl-tRNA synthetase (EC 6.1.1.12) (Aspartate--tRNA ligase)(AspRS) (Antigen T5).	13092719.1.pdb	1l0wA (LIGASE)	50	1.48	1g51B (LIGASE)	100	100	87.5	100	AMO				DB01073 (Fludarabine)
Mycobacterium leprae.	P45488	Adenosylmethionine-8-amino-7-oxononanoate aminotransferase(EC 2.6.1.62) (7,8-diamino-pelargonic acid aminotransferase) (DAPAaminotransferase).	13093168.2.pdb	1s0aA (TRANSFERASE)	48	1.68	1qj3B (AMINOTRANSFERASE)	86.67	100	85.71	100	KAP				DB00302 (Tranexamic Acid)
Mycobacterium leprae.	P46711	Triosephosphate isomerase (EC 5.3.1.1) (TIM) (Triose-phosphateisomerase).	13092770.1.pdb	2btmA (ISOMERASE)	45	1.68	1tpfB (ISOMERASE(INTRAMOLECULAR)	100	100	100	100	DMS	DB01093 (Dimethyl sulfoxide)	DB01093 (Dimethyl sulfoxide)	DB01093 (Dimethyl sulfoxide)	DB01093 (Dimethyl sulfoxide)
Mycobacterium leprae.	P46723	Delta-aminolevulinic acid dehydratase (EC 4.2.1.24) (Porphobilinogensynthase) (ALAD (ALADH).	13093994.1.pdb	1h7nA (DEHYDRATASE)	37	1.55	1h7oA (DEHYDRATASE)	100	100	84.62	100	DAV				DB00855 (Aminolevulinic acid)
Mycobacterium leprae.	P46723	Delta-aminolevulinic acid dehydratase (EC 4.2.1.24) (Porphobilinogensynthase) (ALAD (ALADH).	13093994.3.pdb	2c1hA (LYASE)	47	1.55	1b4eA (LYASE)	100	100	100	100	LEA			DB00510 (Divalproex sodium) DB00313 (Valproic Acid)	DB00510 (Divalproex sodium) DB00313 (Valproic Acid)
Mycobacterium leprae.	P46723	Delta-aminolevulinic acid dehydratase (EC 4.2.1.24) (Porphobilinogensynthase) (ALAD (ALADH).	13093994.3.pdb	2c1hA (LYASE)	47	1.55	1l6yA (LYASE)	80	100	76.19	100	4OX				DB00548 (Azelaic Acid)
Mycobacterium leprae.	P46723	Delta-aminolevulinic acid dehydratase (EC 4.2.1.24) (Porphobilinogensynthase) (ALAD (ALADH).	13093994.1.pdb	1h7nA (DEHYDRATASE)	37	1.55	1gjpA (DEHYDRATASE)	94.74	100	76.19	100	4OX				DB00548 (Azelaic Acid)
Mycobacterium leprae.	P46723	Delta-aminolevulinic acid dehydratase (EC 4.2.1.24) (Porphobilinogensynthase) (ALAD (ALADH).	13093994.1.pdb	1h7nA (DEHYDRATASE)	37	1.55	1eb3A (DEHYDRATASE)	100	100	83.33	100	DSB				DB00548 (Azelaic Acid)
Mycobacterium leprae.	P46723	Delta-aminolevulinic acid dehydratase (EC 4.2.1.24) (Porphobilinogensynthase) (ALAD (ALADH).	13093994.1.pdb	1h7nA (DEHYDRATASE)	37	1.55	1b4kB (LYASE)	92.86	100	92.31	100	LEA			DB00510 (Divalproex sodium) DB00313 (Valproic Acid)	DB00510 (Divalproex sodium) DB00313 (Valproic Acid)

Organism	UniProt Id	Target description	3D model id	PDB Template	Model sequence identity	ModPipe Quality Score	PDB Expansion id	Binding site expansion sequence identity	Binding site expansion structure identity	Binding site sequence identity	Binding site structure identity	PDB Ligand id	Drugbank (Exact)	Drugbank (supra-structure)	Drugbank (sub-structure)	Drugbank (similar)
Mycobacterium leprae.	P46723	Delta-aminolevulinic acid dehydratase (EC 4.2.1.24) (Porphobilinogensynthase) (ALAD) (ALADH).	13093994.1.pdb	1h7nA (DEHYDRATASE)	37	1.55	1h7rA (DEHYDRATASE)	100	100	91.67	100	SHU				DB00548 (Azelaic Acid)
Mycobacterium leprae.	P46723	Delta-aminolevulinic acid dehydratase (EC 4.2.1.24) (Porphobilinogensynthase) (ALAD) (ALADH).	13093994.2.pdb	1w5qA (SYNTHASE)	46	1.66	1l6sA (LYASE)	82.35	94.44	93.75	100	DSB				DB00548 (Azelaic Acid)
Mycobacterium leprae.	P46862	Purine nucleoside phosphorylase (EC 2.4.2.1) (Inosine phosphorylase)(PNP).	13092848.2.pdb	1g2oA (TRANSFERASE)	82	2.05	1a9s_ (PENTOSYLTRANSFERASE)	83.33	90	88.24	100	NOS			DB01280 (Nelarabine)	DB01280 (Nelarabine)
Mycobacterium leprae.	P46862	Purine nucleoside phosphorylase (EC 2.4.2.1) (Inosine phosphorylase)(PNP).	13092848.1.pdb	1qe5A (TRANSFERASE)	55	1.71	1vuD (TRANSFERASE)	100	100	84.21	100	9PP		DB00300 (Tenofovir)		DB00300 (Tenofovir)
Trypanosoma brucei brucei.	P50098	Inosine-5'-monophosphate dehydrogenase (EC 1.1.1.205) (IMPdehydrogenase) (IMPDH) (IMPD).	Tb10.61.0150.1.pdb	1jr1A (OXIDOREDUCTASE)	57	1.47	1jr1B (OXIDOREDUCTASE)	100	100	100	100	MOA	DB01024 (Mycophenolic acid)	DB01024 (Mycophenolic acid)	DB00688 (Mycophenolate mofetil) DB01024 (Mycophenolic acid)	DB00688 (Mycophenolate mofetil) DB01024 (Mycophenolic acid)
Plasmodium falciparum	P50250	Adenosylhomocysteinase (EC 3.3.1.1) (S-adenosyl-L-homocysteinehydrolase) (AdoHcyase) (PfSAHH).	PFE1050w.1.pdb	1li4A (HYDROLASE)	55	1.37	1ky5D (HYDROLASE)	100	100	82.61	95.65	ADY				DB00194 (Vidarabine)
Plasmodium falciparum	P50250	Adenosylhomocysteinase (EC 3.3.1.1) (S-adenosyl-L-homocysteinehydrolase) (AdoHcyase) (PfSAHH).	PFE1050w.2.pdb	1v8bA (HYDROLASE)	100	2.24	1d4fB (HYDROLASE)	91.3	100	100	100	ADN			DB00194 (Vidarabine)	DB00242 (Cladribine) DB00194 (Vidarabine)
Plasmodium falciparum	P50250	Adenosylhomocysteinase (EC 3.3.1.1) (S-adenosyl-L-homocysteinehydrolase) (AdoHcyase) (PfSAHH).	PFE1050w.1.pdb	1li4A (HYDROLASE)	55	1.37	1d4fD (HYDROLASE)	100	100	82.61	95.65	ADN			DB00194 (Vidarabine)	DB00242 (Cladribine) DB00194 (Vidarabine)
Plasmodium falciparum	P62203	Calmodulin (CaM).	PF14_0323.1.pdb	1rfjA (METAL BINDING PROTEIN)	87	2.04	1cll_ (CALCIUM-BINDING PROTEIN)	83.33	100	80	100	EOH	DB00898 (Ethanol)	DB00898 (Ethanol)	DB00898 (Ethanol)	DB00898 (Ethanol)
Mycobacterium tuberculosis.	P65232	Ribose-phosphate pyrophosphokinase (EC 2.7.6.1) (RPPK) (Phosphoribosylpyrophosphate synthetase) (P-Rib-PP synthetase) (PRPP synthetase).	15608157.1.pdb	1dkuA (TRANSFERASE)	46	1.55	1dkuA (TRANSFERASE)	100	100	75	100	ABM				DB01073 (Fludarabine)
Mycobacterium tuberculosis.	P65487	Probable methylmalonyl-CoA mutase large subunit (EC 5.4.99.2) (MCM).	15608631.1.pdb	1reqA (ISOMERASE)	71	1.86	4reqA (ISOMERASE)	100	100	100	100	5AD			DB00194 (Vidarabine)	DB00242 (Cladribine) DB00194 (Vidarabine)
Mycobacterium tuberculosis.	P67032	Glycyl-tRNA synthetase (EC 6.1.1.14) (Glycine--tRNA ligase) (GlyRS).	15609494.1.pdb	1atiA (PROTEIN BIOSYNTHESIS)	48	1.42	1ggmB (AMINOACYL-TRNA SYNTHASE)	100	100	85.19	100	GAP				DB01073 (Fludarabine)
Mycobacterium tuberculosis.	P69167	3-alpha-(or 20-beta)-hydroxysteroid dehydrogenase (EC 1.1.1.53).	15609139.1.pdb	1nffA (OXIDOREDUCTASE)	99	2.2	1nfqC (OXIDOREDUCTASE)	100	100	100	100	AE2				DB01586 (Ursodeoxycholic acid)
Leishmania major.	Q01782	Pteridine reductase 1 (EC 1.5.1.33) (H region methotrexate resistanceprotein).	LmjF23.0270.2.pdb	1e7wA (DIHYDROFOLATE REDUCTASE)	100	2.16	1e7wA (DIHYDROFOLATE REDUCTASE)	100	100	100	100	MTX	DB00563 (Methotrexate)	DB00563 (Methotrexate)	DB00563 (Methotrexate)	DB00563 (Methotrexate)
Plasmodium falciparum.	Q02155	Hexokinase (EC 2.7.1.1).	PFF1155w.2.pdb	1bdg_ (HEXOKINASE)	34	1.33	1e7wA (DIHYDROFOLATE REDUCTASE)	100	100	80	100	SUL			DB00653 (Magnesium Sulfate)	DB00653 (Magnesium Sulfate)
Trypanosoma cruzi.	Q09734	Macrophage infectivity potentiator precursor (Peptidyl-prolyl cis-trans isomerase) (EC 5.2.1.8) (PPlase) (Rotamase).	Tc00.1047053508897.1.5.pdb	1jvwA (ISOMERASE)	100	1.96	1d7hB (ISOMERASE)	87.5	100	100	100	DMS	DB01093 (Dimethyl sulfoxide)	DB01093 (Dimethyl sulfoxide)	DB01093 (Dimethyl sulfoxide)	DB01093 (Dimethyl sulfoxide)
Trypanosoma cruzi.	Q09734	Macrophage infectivity potentiator precursor (Peptidyl-prolyl cis-trans isomerase) (EC 5.2.1.8) (PPlase) (Rotamase).	Tc00.1047053508897.1.5.pdb	1jvwA (ISOMERASE)	100	1.96	1tcoC (COMPLEX (HYDROLASE/ISOMERASE))	80	100	100	100	FK5	DB00864 (Tacrolimus)	DB00864 (Tacrolimus)	DB00864 (Tacrolimus)	DB00864 (Tacrolimus) DB00337 (Pimecrolimus)
Trypanosoma cruzi.	Q09734	Macrophage infectivity potentiator precursor (Peptidyl-prolyl cis-trans isomerase) (EC 5.2.1.8) (PPlase) (Rotamase).	Tc00.1047053508897.1.5.pdb	1jvwA (ISOMERASE)	100	1.96	1fkd_ (CIS-TRANS ISOMERASE)	81.25	100	100	100	818				DB00864 (Tacrolimus) DB00337 (Pimecrolimus)
Mycobacterium tuberculosis.	Q10399	Probable branched-chain-amino-acid aminotransferase (EC 2.6.1.42)(BCAT).	15609347.1.pdb	2coiA (TRANSFERASE)	37	1.45	1i1mB (TRANSFERASE)	81.82	84.62	81.82	100	4MV				DB00510 (Divalproex sodium) DB00313 (Valproic Acid)
Mycobacterium tuberculosis.	Q10761	Fumarate reductase iron-sulfur subunit (EC 1.3.99.1).	15608691.1.pdb	1kf6B (OXIDOREDUCTASE)	50	1.63	1l0vN (OXIDOREDUCTASE)	100	100	80	100	MQ7				DB01022 (Phytonadione)
Leishmania major.	Q18L72	Aspartate aminotransferase (EC 2.6.1.1).	LmjF35.0820.2.pdb	2cstA (TRANSFERASE(AMINOTRANSFERASE))	43	1.64	2ay2B (AMINOTRANSFERASE)	78.57	100	76.92	100	CXP				DB00548 (Azelaic Acid)
Toxoplasma gondii.	Q1JSX8	Isoleucine trna synthetase, putative (EC 6.1.1.5).	25.m01749.1.pdb	1ile_ (AMINOACYL-TRNA SYNTHETASE)	39	1.24	2ay2B (AMINOTRANSFERASE)	78.57	100	75	100	MRC	DB00410 (Mupirocin)	DB00410 (Mupirocin)	DB00410 (Mupirocin)	DB00410 (Mupirocin)
Toxoplasma gondii RH.	Q1JTE1	Type I fatty acid synthase, putative.	57.m03124.1.pdb	1pg4A (LIGASE)	54	1.7	1pg4B (LIGASE)	100	100	80.95	100	PRX		DB00194 (Vidarabine)		DB01073 (Fludarabine) DB00194 (Vidarabine)
Toxoplasma gondii RH.	Q1JTK8	Nucleoside diphosphate kinase, putative (EC 2.7.4.6).	86.m00001.1.pdb	1s57A (TRANSFERASE)	57	1.77	1nhkR (PHOSPHOTRANSFERASE)	76.92	100	91.67	100	CMP				DB01073 (Fludarabine)
Toxoplasma gondii.	Q1KSE2	Triosephosphate isomerase (EC 5.3.1.1).	42.m00050.2.pdb	1r2rA (ISOMERASE)	52	1.71	1tpfA (ISOMERASE(INTRAMOLECULAR)	100	100	83.33	100	DMS	DB01093 (Dimethyl sulfoxide)	DB01093 (Dimethyl sulfoxide)	DB01093 (Dimethyl sulfoxide)	DB01093 (Dimethyl sulfoxide)
Toxoplasma gondii.	Q27006	Protein serine/threonine kinase.	49.m00046.2.pdb	2h6dA (SIGNALING PROTEIN,TRANSFERASE)	39	1.19	1fmoE (COMPLEX (PHOSPHOTRANSFERASE/INHIBITOR))	82.35	89.47	87.5	100	ADN			DB00194 (Vidarabine)	DB00242 (Cladribine) DB00194 (Vidarabine)
Cryptosporidium parvum.	Q27552	Bifunctional dihydrofolate reductase-thymidylate synthase.	cgd4_4460.2.pdb	2tsrA (METHYLTRANSFERASE)	56	1.37	1jutB (TRANSFERASE)	91.67	80	90.91	100	LYD				DB00642 (Pemetrexed)
Cryptosporidium parvum.	Q27552	Bifunctional dihydrofolate reductase-thymidylate synthase.	cgd4_4460.2.pdb	2tsrA (METHYLTRANSFERASE)	56	1.37	1tdb_ (TRANSFERASE (METHYLTRANFERASE))	92.31	100	90.48	100	UFP		DB00322 (Floxuridine)		DB00322 (Floxuridine)
Cryptosporidium parvum.	Q27552	Bifunctional dihydrofolate reductase-thymidylate synthase.	cgd4_4460.2.pdb	2tsrA (METHYLTRANSFERASE)	56	1.37	1ju6D (TRANSFERASE)	100	76.47	91.67	100	LYA	DB00642 (Pemetrexed)	DB00642 (Pemetrexed)	DB00642 (Pemetrexed)	DB00642 (Pemetrexed)
Cryptosporidium parvum.	Q27552	Bifunctional dihydrofolate reductase-thymidylate synthase.	cgd4_4460.2.pdb	2tsrA (METHYLTRANSFERASE)	56	1.37	1tduB (METHYLTRANSFERASE)	86.67	100	82.35	100	DUR			DB00322 (Floxuridine) DB00432 (Trifluridine)	DB00322 (Floxuridine) DB00432 (Trifluridine)
Cryptosporidium parvum.	Q27552	Bifunctional dihydrofolate reductase-thymidylate synthase.	cgd4_4460.2.pdb	2tsrA (METHYLTRANSFERASE)	56	1.37	1dduA (METHYLTRANSFERASE)	100	100	84.62	100	DDU			DB00322 (Floxuridine) DB00432 (Trifluridine)	DB00322 (Floxuridine) DB00432 (Trifluridine)
Cryptosporidium parvum.	Q27552	Bifunctional dihydrofolate reductase-thymidylate synthase.	cgd4_4460.2.pdb	2tsrA (METHYLTRANSFERASE)	56	1.37	1axwB (METHYLTRANSFERASE)	76.47	77.27	81.25	100	MTX	DB00563 (Methotrexate)	DB00563 (Methotrexate)	DB00563 (Methotrexate)	DB00563 (Methotrexate)
Cryptosporidium parvum.	Q27552	Bifunctional dihydrofolate reductase-thymidylate synthase.	cgd4_4460.2.pdb	2tsrA (METHYLTRANSFERASE)	56	1.37	1f4fB (TRANSFERASE)	100	100	87.5	100	SUL			DB00653 (Magnesium Sulfate)	DB00653 (Magnesium Sulfate)
Leishmania major.	Q27687	Putative uncharacterized protein pkac1 (EC 2.7.1.37) (Protein kinase Acatalytic subunit).	LmjF18.1080.2.pdb	1rdqE (TRANSFERASE/TRANSFERASE INHIBITOR)	49	1.66	1fmoE (COMPLEX (PHOSPHOTRANSFERASE/INHIBITOR))	100	100	94.44	100	ADN			DB00194 (Vidarabine)	DB00242 (Cladribine) DB00194 (Vidarabine)
Plasmodium falciparum	Q27743	L-lactate dehydrogenase (EC 1.1.1.27) (LDH-P).	PF13_0141.2.pdb	1t2dA (OXIDOREDUCTASE)	100	2.33	1cetA (OXIDOREDUCTASE)	83.33	100	100	100	CLQ	DB00608 (Chloroquine)	DB00608 (Chloroquine)	DB01611 (Hydroxychloroquine) DB00608 (Chloroquine)	DB01611 (Hydroxychloroquine) DB00608 (Chloroquine)
Trypanosoma cruzi.	Q27793	Bifunctional dihydrofolate reductase-thymidylate synthase (DHFR-TS)[Includes: Dihydrofolate reductase (EC 1.5.1.3); Thymidylate synthase(EC 2.1.1.45)].	Tc00.1047053509153.9.1.pdb	1qzfA (OXIDOREDUCTASE, TRANSFERASE)	45	1.42	2tdd_ (TRANSFERASE (METHYLTRANFERASE))	93.33	100	95.45	100	UFP		DB00322 (Floxuridine)		DB00322 (Floxuridine)
Trypanosoma cruzi.	Q27793	Bifunctional dihydrofolate reductase-thymidylate synthase (DHFR-TS)[Includes: Dihydrofolate reductase (EC 1.5.1.3); Thymidylate synthase(EC 2.1.1.45)].	Tc00.1047053509153.9.1.pdb	1qzfA (OXIDOREDUCTASE, TRANSFERASE)	45	1.42	1ju6D (TRANSFERASE)	94.12	100	84.21	100	LYA	DB00642 (Pemetrexed)	DB00642 (Pemetrexed)	DB00642 (Pemetrexed)	DB00642 (Pemetrexed)
Trypanosoma cruzi.	Q27793	Bifunctional dihydrofolate reductase-thymidylate synthase (DHFR-TS)[Includes: Dihydrofolate reductase (EC 1.5.1.3); Thymidylate synthase(EC 2.1.1.45)].	Tc00.1047053509153.9.1.pdb	1qzfA (OXIDOREDUCTASE, TRANSFERASE)	45	1.42	1tduA (METHYLTRANSFERASE)	94.12	100	88.24	100	DUR			DB00322 (Floxuridine) DB00432 (Trifluridine)	DB00322 (Floxuridine) DB00432 (Trifluridine)
Trypanosoma cruzi.	Q27793	Bifunctional dihydrofolate reductase-thymidylate synthase (DHFR-TS)[Includes: Dihydrofolate reductase (EC 1.5.1.3); Thymidylate synthase(EC 2.1.1.45)].	Tc00.1047053509153.9.1.pdb	1qzfA (OXIDOREDUCTASE, TRANSFERASE)	45	1.42	1f4fB (TRANSFERASE)	100	100	100	100	SUL			DB00653 (Magnesium Sulfate)	DB00653 (Magnesium Sulfate)
Trypanosoma cruzi.	Q27793	Bifunctional dihydrofolate reductase-thymidylate synthase (DHFR-TS)[Includes: Dihydrofolate reductase (EC 1.5.1.3); Thymidylate synthase(EC 2.1.1.45)].	Tc00.1047053509153.9.1.pdb	1qzfA (OXIDOREDUCTASE, TRANSFERASE)	45	1.42	1jutB (TRANSFERASE)	86.67	100	92.86	100	LYD				DB00642 (Pemetrexed)
Trypanosoma cruzi.	Q27793	Bifunctional dihydrofolate reductase-thymidylate synthase (DHFR-TS)[Includes: Dihydrofolate reductase (EC 1.5.1.3); Thymidylate synthase(EC 2.1.1.45)].	Tc00.1047053509153.9.1.pdb	1qzfA (OXIDOREDUCTASE, TRANSFERASE)	45	1.42	1dduB (METHYLTRANSFERASE)	100	100	92.31	100	DDU			DB00322 (Floxuridine) DB00432 (Trifluridine)	DB00322 (Floxuridine) DB00432 (Trifluridine)
Toxoplasma gondii.	Q2L8W5	2-methylcitrate synthase.	20.m03767.1.pdb	1a59_ (COLD-ACTIVITY)	32	1.27	1jutB (TRANSFERASE)	86.67	100	90.91	100	SUL			DB00653 (Magnesium Sulfate)	DB00653 (Magnesium Sulfate)
Toxoplasma gondii.	Q2Y2R0	Histone deacetylase 3.	50.m03318.2.pdb	1t64A (HYDROLASE)	36	1.06	1c3sA (LYASE)	83.33	80	90.91	100	SHH	DB02546 (Vorinostat)	DB02546 (Vorinostat)	DB02546 (Vorinostat)	DB02546 (Vorinostat)
Trypanosoma brucei.	Q382G4	Ornithine decarboxylase.	Tb11.01.5300.2.pdb	1f3tA (LYASE)	100	2.08	1njjB (LYASE)	100	100	100	100	GET				DB01172 (Kanamycin) DB00798 (Gentamicin) DB00684 (Tobramycin)
Trypanosoma brucei.	Q383X0	Adenosylhomocysteinase (EC 3.3.1.1).	Tb11.01.1350.1.pdb	1li4A (HYDROLASE)	74	1.94	1d4fD (HYDROLASE)	100	100	91.3	100	ADN			DB00194 (Vidarabine)	DB00242 (Cladribine) DB00194 (Vidarabine)
Trypanosoma brucei.	Q383X0	Adenosylhomocysteinase (EC 3.3.1.1).	Tb11.01.1350.1.pdb	1li4A (HYDROLASE)	74	1.94	1ky5D (HYDROLASE)	100	100	91.3	100	ADY				DB00194 (Vidarabine)
Trypanosoma brucei.	Q386J7	Protein kinase, putative (EC 2.7.1.-).	Tb11.02.0640.3.pdb	1z57A (TRANSFERASE)	33	1.13	1d4fD (HYDROLASE)	100	100	83.33	100	ADN			DB00194 (Vidarabine)	DB00242 (Cladribine) DB00194 (Vidarabine)
Trypanosoma brucei.	Q386K8	Phosphoribosylpyrophosphate synthetase, putative (EC 2.7.6.1).	Tb11.02.0530.2.pdb	1dkuA (TRANSFERASE)	44	1.32	1dkuA (TRANSFERASE)	100	100	75	100	ABM				DB01073 (Fludarabine)
Trypanosoma brucei.	Q387V4	Peptidyl-prolyl cis-trans isomerase (EC 5.2.1.8).	Tb10.61.0760.1.pdb	1fd9A (ISOMERASE)	35	1.18	1d7hB (ISOMERASE)	75	100	85.71	100	DMS	DB01093 (Dimethyl sulfoxide)	DB01093 (Dimethyl sulfoxide)	DB01093 (Dimethyl sulfoxide)	DB01093 (Dimethyl sulfoxide)
Trypanosoma brucei.	Q388U5	Protein kinase A catalytic subunit (EC 2.7.1.37).	Tb10.389.0490.1.pdb	1kobA (KINASE)	49	1.67	1d7hB (ISOMERASE)	75	100	88.89	100	ADN			DB00194 (Vidarabine)	DB00242 (Cladribine) DB00194 (Vidarabine)
Trypanosoma brucei.	Q38A32	Phosphoribosylpyrophosphate synthetase, putative (EC 2.7.6.1).	Tb10.6k15.0970.2.pdb	2c4kA (REGULATORY PROTEIN)	44	1.34	1d7hB (ISOMERASE)	75	100	75	100	ABM				DB01073 (Fludarabine)
Trypanosoma brucei.	Q38A55	Isoleucyl-tRNA synthetase, putative (EC 6.1.1.5).	Tb10.6k15.1220.2.pdb	1ile_ (AMINOACYL-TRNA SYNTHETASE)	41	1.3	1d7hB (ISOMERASE)	75	100	79.17	100	MRC	DB00410 (Mupirocin)	DB00410 (Mupirocin)	DB00410 (Mupirocin)	DB00410 (Mupirocin)
Trypanosoma brucei.	Q38BD9	Peptidyl-prolyl cis-trans isomerase (EC 5.2.1.8).	Tb10.70.2600.1.pdb	1c9hA (IMMUNE SYSTEM)	39	1.56	1d7hB (ISOMERASE)	87.5	100	85.71	100	DMS	DB01093 (Dimethyl sulfoxide)	DB01093 (Dimethyl sulfoxide)	DB01093 (Dimethyl sulfoxide)	DB01093 (Dimethyl sulfoxide)
Trypanosoma brucei.	Q38C41	Hexokinase (EC 2.7.1.1).	Tb10.70.5800.1.pdb	1bg3A (HEXOKINASE)	35	1.48	1ig8A (TRANSFERASE)	83.33	100	80	100	SUL			DB00653 (Magnesium Sulfate)	DB00653 (Magnesium Sulfate)
Trypanosoma brucei.	Q38C74	Histone deacetylase 1.	Tb10.70.6220.2.pdb	1t64A (HYDROLASE)	37	1.45	1c3sA (LYASE)	83.33	80	90.91	100	SHH	DB02546 (Vorinostat)	DB02546 (Vorinostat)	DB02546 (Vorinostat)	DB02546 (Vorinostat)
Trypanosoma brucei.	Q38CK1	Calcineurin B subunit, putative.	Tb10.70.7970.1.pdb	1v1gA (SIGNALLING PROTEIN)	39	1.5	1c3sA (LYASE)	83.33	80	80	100	FK5	DB00864 (Tacrolimus)	DB00864 (Tacrolimus)	DB00864 (Tacrolimus)	DB00864 (Tacrolimus) DB00337 (Pimecrolimus)
Trypanosoma brucei.	Q38D10	Iron/ascorbate oxidoreductase family protein, putative.	Tb09.211.4990.1.pdb	1gp6A (OXYGENASE)	28	1.37	1c3sA (LYASE)	83.33	80	100	100	NO	DB00435 (Nitric Oxide)	DB00435 (Nitric Oxide)	DB00435 (Nitric Oxide)	DB00435 (Nitric Oxide)
Trypanosoma brucei.	Q38D65	ADP-ribosylation factor, putative.	Tb09.211.4460.1.pdb	1mozA (PROTEIN BINDING)	79	2	1c3sA (LYASE)	83.33	80	90	100	EOH	DB00898 (Ethanol)	DB00898 (Ethanol)	DB00898 (Ethanol)	DB00898 (Ethanol)
Trypanosoma brucei.	Q38DR5	Protein kinase A catalytic subunit isoform 1.	Tb09.211.2410.1.pdb	2acxA (TRANSFERASE)	53	1.69	1c3sA (LYASE)	83.33	80	88.89	100	ADN			DB00194 (Vidarabine)	DB00242 (Cladribine) DB00194 (Vidarabine)
Trypanosoma brucei.	Q38DS1	Protein kinase A catalytic subunit isoform 2.	Tb09.211.2360.1.pdb	2f2uA (TRANSFERASE)	50	1.64	1c3sA (LYASE)	83.33	80	88.89	100	ADN			DB00194 (Vidarabine)	DB00242 (Cladribine) DB00194 (Vidarabine)
Trypanosoma cruzi.	Q4CQK0	Oxidoreductase, putative (EC 1.2.3.4) (EC 1.6.5.5).	Tc00.1047053510141.1.2.pdb	1odmA (OXIDOREDUCTASE)	33	1.37	1blz_ (B-LACTAM ANTIBIOTIC)	100	100	100	100	NO	DB00435 (Nitric Oxide)	DB00435 (Nitric Oxide)	DB00435 (Nitric Oxide)	DB00435 (Nitric Oxide)
Trypanosoma cruzi.	Q4CRT0	Inosine-adenosine-guanosine-nucleoside hydrolase, putative(EC 3.2.2.1).	Tc00.1047053510315.1.1.pdb	1hozA (HYDROLASE)	63	1.9	1kicB (HYDROLASE)	100	100	92.59	100	NOS			DB01280 (Nelarabine)	DB01280 (Nelarabine)
Trypanosoma cruzi.	Q4CUG6	Inosine-5'-monophosphate dehydrogenase, putative.	Tc00.1047053507211.4.2.pdb	1jr1A (OXIDOREDUCTASE)	51	1.23	1jr1B (OXIDOREDUCTASE)	100	100	100	100	MOA	DB01024 (Mycophenolic acid)	DB01024 (Mycophenolic acid)	DB00688 (Mycophenolate mofetil) DB01024 (Mycophenolic acid)	DB00688 (Mycophenolate mofetil) DB01024 (Mycophenolic acid)
Trypanosoma cruzi.	Q4CUG6	Inosine-5'-monophosphate dehydrogenase, putative.	Tc00.1047053507211.4.1.pdb	1b3oB (DEHYDROGENASE)	54	1.39	1jr1A (OXIDOREDUCTASE)	91.67	85.71	83.33	100	MOA	DB01024 (Mycophenolic acid)	DB01024 (Mycophenolic acid)	DB00688 (Mycophenolate mofetil) DB01024 (Mycophenolic acid)	DB00688 (Mycophenolate mofetil) DB01024 (Mycophenolic acid)
Trypanosoma cruzi.	Q4CVK9	Adenosine kinase, putative (EC 2.7.1.20).	Tc00.1047053509051.4.2.pdb	1bx4A (TRANSFERASE)	42	1.59	1bx4A (TRANSFERASE)	100	100	76.47	100	ADN			DB00194 (Vidarabine)	DB00242 (Cladribine) DB00194 (Vidarabine)
Trypanosoma cruzi.	Q4CY23	Peptidyl-prolyl cis-trans isomerase (EC 5.2.1.8).	Tc00.1047053506581.1.2.pdb	1rot_ (ROTAMASE (ISOMERASE))	41	1.47	1bx4A (TRANSFERASE)	100	100	85.71	100	DMS	DB01093 (Dimethyl sulfoxide)	DB01093 (Dimethyl sulfoxide)	DB01093 (Dimethyl sulfoxide)	DB01093 (Dimethyl sulfoxide)
Trypanosoma cruzi.	Q4D137	Calmodulin.	Tc00.1047053507483.5.1.pdb	1rfjA (METAL BINDING PROTEIN)	90	2.08	1cll_ (CALCIUM-BINDING PROTEIN)	83.33	100	100	100	EOH	DB00898 (Ethanol)	DB00898 (Ethanol)	DB00898 (Ethanol)	DB00898 (Ethanol)
Trypanosoma cruzi.	Q4D139	Calmodulin, putative.	Tc00.1047053506391.1.1.pdb	1qtxA (SIGNALING PROTEIN)	91	2.07	1dtlA (STRUCTURAL PROTEIN)	82.35	89.47	93.75	100	BEP	DB01244 (Bepridil)	DB01244 (Bepridil)	DB01244 (Bepridil)	DB01244 (Bepridil)
Trypanosoma cruzi.	Q4D139	Calmodulin, putative.	Tc00.1047053507483.5.3.pdb	1qx2A (SIGNALING PROTEIN)	52	1.19	1lin_ (CALCIUM-BINDING PROTEIN)	80	100	77.78	100	TFP	DB00831 (Trifluoperazine)	DB00831 (Trifluoperazine) DB00508 (Triflupromazine)	DB00831 (Trifluoperazine) DB00623 (Fluphenazine)	DB00831 (Trifluoperazine) DB00508 (Triflupromazine) DB00623 (Fluphenazine)
Trypanosoma cruzi.	Q4D139	Calmodulin, putative.	Tc00.1047053506391.1.1.pdb	1qtxA (SIGNALING PROTEIN)	91	2.07	1l7zA (METAL BINDING PROTEIN/PROTEIN BINDING)	100	100	100	100	MYR				DB00313 (Valproic Acid) DB00548 (Azelaic Acid)
Trypanosoma cruzi.	Q4D139	Calmodulin, putative.	Tc00.1047053506391.1.1.pdb	1qtxA (SIGNALING PROTEIN)	91	2.07	1lin_ (CALCIUM-BINDING PROTEIN)	100	100	93.94	100	TFP	DB00831 (Trifluoperazine)	DB00831 (Trifluoperazine) DB00508 (Triflupromazine)	DB00831 (Trifluoperazine) DB00623 (Fluphenazine)	DB00831 (Trifluoperazine) DB00508 (Triflupromazine) DB00623 (Fluphenazine)
Trypanosoma cruzi.	Q4D1Q4	Aspartate aminotransferase, mitochondrial, putative.	Tc00.1047053510945.7.2.pdb	7aatA (TRANSFERASE(AMINOTRANSFERASE))	46	1.65	2ay2B (AMINOTRANSFERASE)	100	100	76.92	100	CXP				DB00548 (Azelaic Acid)
Trypanosoma cruzi.	Q4D2F2	Isoleucyl-tRNA synthetase, putative (EC 6.1.1.5).	Tc00.1047053509797.4.2.pdb	1ile_ (AMINOACYL-TRNA SYNTHETASE)	39	1.11	2ay2B (AMINOTRANSFERASE)	100	100	79.17	100	MRC	DB00410 (Mupirocin)	DB00410 (Mupirocin)	DB00410 (Mupirocin)	DB00410 (Mupirocin)
Trypanosoma cruzi.	Q4D3B1	Serine/threonine protein phosphatase (EC 3.1.3.16).	Tc00.1047053508413.4.4.pdb	1auiA (HYDROLASE)	52	1.52	1tcoA (COMPLEX (HYDROLASE/ISOMERASE))	100	100	100	100	FK5	DB00864 (Tacrolimus)	DB00864 (Tacrolimus)	DB00864 (Tacrolimus)	DB00864 (Tacrolimus) DB00337 (Pimecrolimus)
Trypanosoma cruzi.	Q4D455	Adenosylhomocysteinase (EC 3.3.1.1).	Tc00.1047053511229.5.1.pdb	1li4A (HYDROLASE)	74	1.82	1d4fD (HYDROLASE)	100	100	91.3	100	ADN			DB00194 (Vidarabine)	DB00242 (Cladribine) DB00194 (Vidarabine)

Organism	UniProt Id	Target description	3D model id	PDB Template	Model sequence identity	ModPipe Quality Score	PDB Expansion id	Binding site expansion sequence identity	Binding site expansion structure identity	Binding site sequence identity	Binding site structure identity	PDB Ligand id	Drugbank (Exact)	Drugbank (supra-structure)	Drugbank (sub-structure)	Drugbank (similar)
Trypanosoma cruzi.	Q4D455	Adenosylhomocysteinase (EC 3.3.1.1).	Tc00.1047053511229.5.1.pdb	1li4A (HYDROLASE)	74	1.82	1ky5D (HYDROLASE)	100	100	91.3	100	ADY				DB00194 (Vidarabine)
Trypanosoma cruzi.	Q4D4C9	3-hydroxy-3-methylglutaryl-CoA reductase, putative.	Tc00.1047053506831.4.1.pdb	1dqaA (OXIDOREDUCTASE)	52	1.62	1hwkD (OXIDOREDUCTASE)	100	100	80	100	117	DB01076 (Atorvastatin)	DB01076 (Atorvastatin)	DB01076 (Atorvastatin)	DB01076 (Atorvastatin)
Trypanosoma cruzi.	Q4D4Z9	Protein kinase A catalytic subunit, putative (EC 2.7.1.37).	Tc00.1047053511269.5.2.pdb	1rdqE (TRANSFERASE/TRANSFERASE INHIBITOR)	51	1.68	1fmoE (COMPLEX (PHOSPHOTRANSFERASE/INHIBITOR))	100	100	94.44	100	ADN			DB00194 (Vidarabine)	DB00242 (Cladribine) DB00194 (Vidarabine)
Trypanosoma cruzi.	Q4D5W5	Peptidyl-prolyl cis-trans isomerase (EC 5.2.1.8).	Tc00.1047053508169.6.1.pdb	1bkf_ (ISOMERASE)	51	1.68	1fmoE (COMPLEX (PHOSPHOTRANSFERASE/INHIBITOR))	100	100	85.71	100	DMS	DB01093 (Dimethyl sulfoxide)	DB01093 (Dimethyl sulfoxide)	DB01093 (Dimethyl sulfoxide)	DB01093 (Dimethyl sulfoxide)
Trypanosoma cruzi.	Q4D762	Histone deacetylase, putative.	Tc00.1047053504159.8.2.pdb	1t64A (HYDROLASE)	32	1.05	1c3sA (LYASE)	83.33	80	81.82	100	SHH	DB02546 (Vorinostat)	DB02546 (Vorinostat)	DB02546 (Vorinostat)	DB02546 (Vorinostat)
Trypanosoma cruzi.	Q4D7X9	Serine/threonine protein phosphatase (EC 3.1.3.16).	Tc00.1047053510755.1.4.pdb	1auiA (HYDROLASE)	52	1.58	1tcoA (COMPLEX (HYDROLASE/ISOMERASE))	100	100	100	100	FK5	DB00864 (Tacrolimus)	DB00864 (Tacrolimus)	DB00864 (Tacrolimus)	DB00864 (Tacrolimus) DB00337 (Pimecrolimus)
Trypanosoma cruzi.	Q4D7Y8	ADP-ribosylation factor 1, putative.	Tc00.1047053508415.5.3.pdb	1r8sA (PROTEIN TRANSPORT/EXCHANGE FACTOR)	80	2	1mr3F (SIGNALING PROTEIN)	75	100	80	100	EOH	DB00898 (Ethanol)	DB00898 (Ethanol)	DB00898 (Ethanol)	DB00898 (Ethanol)
Trypanosoma cruzi.	Q4D9I0	Phosphoribosylpyrophosphate synthetase, putative (EC 2.7.6.1).	Tc00.1047053508717.3.2.pdb	1dkuA (TRANSFERASE)	43	1.29	1dkuA (TRANSFERASE)	100	100	75	100	ABM				DB01073 (Fludarabine)
Trypanosoma cruzi.	Q4DC56	Mannosyl-oligosaccharide 1,2-alpha-mannosidase IB, putative.	Tc00.1047053508317.8.1.pdb	1nxcA (HYDROLASE)	30	1.01	1kreB (HYDROLASE)	100	100	100	100	DMJ			DB00491 (Miglitol) DB00419 (Miglustat)	DB00491 (Miglitol) DB00419 (Miglustat)
Trypanosoma cruzi.	Q4DCT8	Mannosyl-oligosaccharide 1,2-alpha-mannosidase IB, putative.	Tc00.1047053508317.1.4.pdb	1kktA (HYDROLASE)	30	1.07	1kreB (HYDROLASE)	100	100	100	100	DMJ			DB00491 (Miglitol) DB00419 (Miglustat)	DB00491 (Miglitol) DB00419 (Miglustat)
Trypanosoma cruzi.	Q4DCU1	Phosphoribosylpyrophosphate synthetase, putative (EC 2.7.6.1).	Tc00.1047053506839.8.3.pdb	1dkuA (TRANSFERASE)	45	1.42	1dkuA (TRANSFERASE)	100	100	75	100	ABM				DB01073 (Fludarabine)
Trypanosoma cruzi.	Q4DFL5	Peptidyl-prolyl cis-trans isomerase (EC 5.2.1.8).	Tc00.1047053508323.8.2.pdb	1yat_ (BINDING PROTEIN)	50	1.64	1dkuA (TRANSFERASE)	100	100	80	100	818				DB00864 (Tacrolimus) DB00337 (Pimecrolimus)
Trypanosoma cruzi.	Q4DFL5	Peptidyl-prolyl cis-trans isomerase (EC 5.2.1.8).	Tc00.1047053508323.8.2.pdb	1yat_ (BINDING PROTEIN)	50	1.64	1dkuA (TRANSFERASE)	100	100	75	100	FK5	DB00864 (Tacrolimus)	DB00864 (Tacrolimus)	DB00864 (Tacrolimus)	DB00864 (Tacrolimus) DB00337 (Pimecrolimus)
Trypanosoma cruzi.	Q4DLI6	Histone deacetylase 1, putative.	Tc00.1047053508637.1.4.pdb	1c3pA (LYASE)	28	1.34	1c3sA (LYASE)	100	100	78.57	100	SHH	DB02546 (Vorinostat)	DB02546 (Vorinostat)	DB02546 (Vorinostat)	DB02546 (Vorinostat)
Trypanosoma cruzi.	Q4DM82	Inosine-5'-monophosphate dehydrogenase, putative.	Tc00.1047053511301.1.6.pdb	1b3oB (DEHYDROGENASE)	55	1.41	1jr1A (OXIDOREDUCTASE)	91.67	85.71	83.33	100	MOA	DB01024 (Mycophenolic acid)	DB01024 (Mycophenolic acid)	DB00688 (Mycophenolate mofetil) DB01024 (Mycophenolic acid)	DB00688 (Mycophenolate mofetil) DB01024 (Mycophenolic acid)
Trypanosoma cruzi.	Q4DN66	Calmodulin, putative.	Tc00.1047053511233.8.1.pdb	1qtxA (SIGNALING PROTEIN)	50	1.65	1dtlA (STRUCTURAL PROTEIN)	82.35	89.47	75	100	BEP	DB01244 (Bepridil)	DB01244 (Bepridil)	DB01244 (Bepridil)	DB01244 (Bepridil)
Trypanosoma cruzi.	Q4DNN1	Histone deacetylase, putative.	Tc00.1047053506821.1.6.pdb	1t64A (HYDROLASE)	31	1.04	1c3sA (LYASE)	83.33	80	81.82	100	SHH	DB02546 (Vorinostat)	DB02546 (Vorinostat)	DB02546 (Vorinostat)	DB02546 (Vorinostat)
Trypanosoma cruzi.	Q4DPG9	Serine/threonine protein kinase, putative.	Tc00.1047053510861.1.2.pdb	2h6dA (SIGNALING PROTEIN,TRANSFERASE)	45	1.54	1fmoE (COMPLEX (PHOSPHOTRANSFERASE/INHIBITOR))	82.35	89.47	75	100	ADN			DB00194 (Vidarabine)	DB00242 (Cladribine) DB00194 (Vidarabine)
Trypanosoma cruzi.	Q4DQ27	Hexokinase, putative (EC 2.7.1.1).	Tc00.1047053510121.2.2.pdb	1ig8A (TRANSFERASE)	36	1.48	1ig8A (TRANSFERASE)	100	100	80	100	SUL			DB00653 (Magnesium Sulfate)	DB00653 (Magnesium Sulfate)
Trypanosoma cruzi.	Q4DQR5	3-hydroxy-3-methylglutaryl-CoA reductase, putative.	Tc00.1047053509167.2.1.pdb	1dqaA (OXIDOREDUCTASE)	52	1.59	1hwkD (OXIDOREDUCTASE)	100	100	80	100	117	DB01076 (Atorvastatin)	DB01076 (Atorvastatin)	DB01076 (Atorvastatin)	DB01076 (Atorvastatin)
Trypanosoma cruzi.	Q4DQU7	Histone deacetylase 1, putative.	Tc00.1047053511911.1.4.pdb	1t64A (HYDROLASE)	36	1.53	1c3sA (LYASE)	83.33	80	90.91	100	SHH	DB02546 (Vorinostat)	DB02546 (Vorinostat)	DB02546 (Vorinostat)	DB02546 (Vorinostat)
Trypanosoma cruzi.	Q4DQY7	Acetyl-CoA synthetase, putative (EC 6.2.1.1).	Tc00.1047053509331.3.1.pdb	1pg4A (LIGASE)	52	1.69	1pg4B (LIGASE)	100	100	95.24	100	PRX		DB00194 (Vidarabine)		DB01073 (Fludarabine) DB00194 (Vidarabine)
Trypanosoma cruzi.	Q4DTW1	DNA topoisomerase IB, large subunit, putative (EC 5.99.1.2).	Tc00.1047053508693.2.1.pdb	2b9sA (ISOMERASE/DNA)	77	1.43	1k4tA (ISOMERASE/DNA)	100	75	100	100	TTC	DB01030 (Topotecan)	DB01030 (Topotecan)	DB01030 (Topotecan)	DB00762 (Irinotecan) DB01030 (Topotecan)
Trypanosoma cruzi.	Q4DTW1	DNA topoisomerase IB, large subunit, putative (EC 5.99.1.2).	Tc00.1047053508693.2.1.pdb	2b9sA (ISOMERASE/DNA)	77	1.43	1k4tA (ISOMERASE/DNA)	100	75	100	100	TTG				DB01030 (Topotecan)
Trypanosoma cruzi.	Q4DV43	Triosephosphate isomerase (EC 5.3.1.1).	Tc00.1047053508647.2.3.pdb	1n55A (ISOMERASE)	68	2.02	1tpfB (ISOMERASE(INTRAMOLECULAR)	100	100	91.67	100	DMS	DB01093 (Dimethyl sulfoxide)	DB01093 (Dimethyl sulfoxide)	DB01093 (Dimethyl sulfoxide)	DB01093 (Dimethyl sulfoxide)
Trypanosoma cruzi.	Q4DWT0	Oxidoreductase, putative (EC 1.2.3.4) (EC 1.6.5.5).	Tc00.1047053508409.1.3.pdb	1odmA (OXIDOREDUCTASE)	33	1.36	1blz_ (B-LACTAM ANTIBIOTIC)	100	100	100	100	NO	DB00435 (Nitric Oxide)	DB00435 (Nitric Oxide)	DB00435 (Nitric Oxide)	DB00435 (Nitric Oxide)
Trypanosoma cruzi.	Q4DXW9	Phosphoribosylpyrophosphate synthetase, putative (EC 2.7.6.1).	Tc00.1047053510431.2.4.pdb	1dkuA (TRANSFERASE)	44	1.49	1dkuA (TRANSFERASE)	100	100	75	100	ABM				DB01073 (Fludarabine)
Trypanosoma cruzi.	Q4DXY0	Protein kinase, putative.	Tc00.1047053510431.1.6.pdb	2eu9A (TRANSFERASE)	34	1.14	1q97B (TRANSFERASE)	76.92	92.86	75	100	ADN			DB00194 (Vidarabine)	DB00242 (Cladribine) DB00194 (Vidarabine)
Trypanosoma cruzi.	Q4DYN2	Acetyl-CoA synthetase, putative (EC 6.2.1.1).	Tc00.1047053504427.1.4.pdb	1pg4A (LIGASE)	51	1.61	1pg4B (LIGASE)	100	100	95.24	100	PRX		DB00194 (Vidarabine)		DB01073 (Fludarabine) DB00194 (Vidarabine)
Trypanosoma cruzi.	Q4E0S4	Adenosine kinase, putative (EC 2.7.1.20).	Tc00.1047053509965.3.3.pdb	1bx4A (TRANSFERASE)	42	1.66	1bx4A (TRANSFERASE)	100	100	76.47	100	ADN			DB00194 (Vidarabine)	DB00242 (Cladribine) DB00194 (Vidarabine)
Trypanosoma cruzi.	Q4E256	Nucleoside diphosphate kinase (EC 2.7.4.6).	Tc00.1047053508707.2.1.pdb	1npk_ (PHOSPHOTRANSFERASE(PO4 AS ACCEPTOR))	59	1.78	1bx4A (TRANSFERASE)	100	100	83.33	100	CMP				DB01073 (Fludarabine)
Trypanosoma cruzi.	Q4E2M9	Glucose-6-phosphate isomerase, glycosomal, putative (EC 5.3.1.9).	Tc00.1047053510889.2.1.pdb	1t10A (ISOMERASE)	74	1.95	1iatA (ISOMERASE)	100	100	100	100	SUL			DB00653 (Magnesium Sulfate)	DB00653 (Magnesium Sulfate)
Trypanosoma cruzi.	Q4E3J2	Serine/threonine protein kinase, putative.	Tc00.1047053510329.2.4.pdb	2h6dA (SIGNALING PROTEIN,TRANSFERASE)	54	1.1	1fmoE (COMPLEX (PHOSPHOTRANSFERASE/INHIBITOR))	82.35	89.47	81.25	100	ADN			DB00194 (Vidarabine)	DB00242 (Cladribine) DB00194 (Vidarabine)
Trypanosoma cruzi.	Q4E4A1	Serine/threonine protein phosphatase 2B catalytic subunit A2, putative(EC 3.1.3.16).	Tc00.1047053510187.5.2.pdb	1auiA (HYDROLASE)	48	1.58	1tcoA (COMPLEX (HYDROLASE/ISOMERASE))	100	100	100	100	FK5	DB00864 (Tacrolimus)	DB00864 (Tacrolimus)	DB00864 (Tacrolimus)	DB00864 (Tacrolimus) DB00337 (Pimecrolimus)
Trypanosoma cruzi.	Q4E4S9	Protein kinase A catalytic subunit isoform 1, putative.	Tc00.1047053508461.3.7.pdb	1rdqE (TRANSFERASE/TRANSFERASE INHIBITOR)	51	1.56	1fmoE (COMPLEX (PHOSPHOTRANSFERASE/INHIBITOR))	100	100	88.89	100	ADN			DB00194 (Vidarabine)	DB00242 (Cladribine) DB00194 (Vidarabine)
Trypanosoma cruzi.	Q4E627	Inosine-adenosine-guanosine-nucleoside hydrolase, putative(EC 3.2.2.1).	Tc00.1047053508153.6.2.pdb	1hozA (HYDROLASE)	63	1.91	1kicB (HYDROLASE)	100	100	92.59	100	NOS			DB01280 (Nelarabine)	DB01280 (Nelarabine)
Toxoplasma gondii.	Q4FAB4	ATP-binding cassette sub-family B member 5.	74.m00467.1.pdb	1g8fA (TRANSFERASE)	32	1.15	1jeeB (TRANSFERASE)	100	100	76.19	100	ADX				DB01073 (Fludarabine)
Toxoplasma gondii.	Q4FAB4	ATP-binding cassette sub-family B member 5.	74.m00467.2.pdb	1m8pA (TRANSFERASE)	41	1.5	1i2dC (TRANSFERASE)	100	100	76.92	100	ADX				DB01073 (Fludarabine)
Leishmania major strain Friedlin.	Q4FW68	Protein kinase A catalytic subunit isoform 1.	LmjF35.4010.2.pdb	1rdqE (TRANSFERASE/TRANSFERASE INHIBITOR)	48	1.55	1fmoE (COMPLEX (PHOSPHOTRANSFERASE/INHIBITOR))	100	100	88.89	100	ADN			DB00194 (Vidarabine)	DB00242 (Cladribine) DB00194 (Vidarabine)
Leishmania major strain Friedlin.	Q4FWZ9	NADH-dependent fumarate reductase, putative.	LmjF35.1190.1.pdb	1y0pA (OXIDOREDUCTASE)	38	1.43	1d4cD (OXIDOREDUCTASE)	100	100	81.82	100	SUL			DB00653 (Magnesium Sulfate)	DB00653 (Magnesium Sulfate)
Leishmania major strain Friedlin.	Q4FYU2	Methylmalonyl-coenzyme a mutase, putative (EC 5.4.99.2).	LmjF27.0300.1.pdb	1reqA (ISOMERASE)	55	1.67	4reqA (ISOMERASE)	100	100	100	100	5AD			DB00194 (Vidarabine)	DB00242 (Cladribine) DB00194 (Vidarabine)
Trypanosoma brucei.	Q4GYJ4	Glucose-6-phosphate isomerase (EC 5.3.1.9) (GPI) (Phosphoglucoseisomerase) (PGI) (Phosphohexose isomerase) (PHI).	Tb927.1.3830.1.pdb	1t10A (ISOMERASE)	71	1.93	1iatA (ISOMERASE)	100	100	100	100	SUL			DB00653 (Magnesium Sulfate)	DB00653 (Magnesium Sulfate)
Plasmodium vivax.	Q4PRK9	L-lactate dehydrogenase (EC 1.1.1.27) (Lactate dehydrogenase).	Pv116630.1.pdb	2a92A (OXIDOREDUCTASE)	100	2.3	1cetA (OXIDOREDUCTASE)	81.82	91.67	90	90	CLQ	DB00608 (Chloroquine)	DB00608 (Chloroquine)	DB01611 (Hydroxychloroquine) DB00608 (Chloroquine)	DB01611 (Hydroxychloroquine) DB00608 (Chloroquine)
Leishmania major.	Q4Q0M2	Phosphoribosylpyrophosphate synthetase, putative (EC 2.7.6.1).	LmjF36.5390.2.pdb	1dkuA (TRANSFERASE)	41	1.37	1dkuA (TRANSFERASE)	100	100	75	100	ABM				DB01073 (Fludarabine)
Leishmania major.	Q4Q124	Adenosylhomocysteinase (EC 3.3.1.1).	LmjF36.3910.1.pdb	1v8bA (HYDROLASE)	54	1.47	1d4fB (HYDROLASE)	91.3	100	82.61	100	ADN			DB00194 (Vidarabine)	DB00242 (Cladribine) DB00194 (Vidarabine)
Leishmania major.	Q4Q124	Adenosylhomocysteinase (EC 3.3.1.1).	LmjF36.3910.2.pdb	1li4A (HYDROLASE)	73	1.97	1ky5D (HYDROLASE)	100	100	91.3	100	ADY				DB00194 (Vidarabine)
Leishmania major.	Q4Q124	Adenosylhomocysteinase (EC 3.3.1.1).	LmjF36.3910.2.pdb	1li4A (HYDROLASE)	73	1.97	1d4fD (HYDROLASE)	100	100	91.3	100	ADN			DB00194 (Vidarabine)	DB00242 (Cladribine) DB00194 (Vidarabine)
Leishmania major.	Q4Q148	Calmodulin, putative.	LmjF36.3675.1.pdb	1rfjA (METAL BINDING PROTEIN)	48	1.58	1cll_ (CALCIUM-BINDING PROTEIN)	83.33	100	80	100	EOH	DB00898 (Ethanol)	DB00898 (Ethanol)	DB00898 (Ethanol)	DB00898 (Ethanol)
Leishmania major.	Q4Q1Y6	Protein kinase, putative (Snf1-related protein kinases, putative).	LmjF36.0900.2.pdb	2h6dA (SIGNALING PROTEIN,TRANSFERASE)	54	1.02	1fmoE (COMPLEX (PHOSPHOTRANSFERASE/INHIBITOR))	82.35	89.47	75	100	ADN			DB00194 (Vidarabine)	DB00242 (Cladribine) DB00194 (Vidarabine)
Leishmania major.	Q4Q255	Peptidyl-prolyl cis-trans isomerase (EC 5.2.1.8).	LmjF36.0230.1.pdb	1bkf_ (ISOMERASE)	50	1.72	1d7hB (ISOMERASE)	75	100	85.71	100	DMS	DB01093 (Dimethyl sulfoxide)	DB01093 (Dimethyl sulfoxide)	DB01093 (Dimethyl sulfoxide)	DB01093 (Dimethyl sulfoxide)
Leishmania major.	Q4Q2K0	DNA topoisomerase IB, large subunit (EC 5.99.1.2).	LmjF34.3440.2.pdb	1a31A (COMPLEX (ISOMERASE/DNA))	48	1.25	1k4tA (ISOMERASE/DNA)	100	87.5	100	100	TTC	DB01030 (Topotecan)	DB01030 (Topotecan)	DB01030 (Topotecan)	DB00762 (Irinotecan) DB01030 (Topotecan)
Leishmania major.	Q4Q2K0	DNA topoisomerase IB, large subunit (EC 5.99.1.2).	LmjF34.3440.2.pdb	1a31A (COMPLEX (ISOMERASE/DNA))	48	1.25	1k4tA (ISOMERASE/DNA)	100	75	100	100	TTG				DB01030 (Topotecan)
Leishmania major.	Q4Q3Z4	Phosphoribosylpyrophosphate synthetase, putative (Phosphoribosyltransferase, putative) (EC 2.7.6.1).	LmjF33.1930.2.pdb	1dkuA (TRANSFERASE)	44	1.5	1dkuA (TRANSFERASE)	100	100	75	100	ABM				DB01073 (Fludarabine)
Leishmania major.	Q4Q5Y0	Adp-ribosylation factor, putative.	LmjF31.2790.3.pdb	1r8sA (PROTEIN TRANSPORT/EXCHANGE FACTOR)	77	1.99	1mr3F (SIGNALING PROTEIN)	75	100	80	100	EOH	DB00898 (Ethanol)	DB00898 (Ethanol)	DB00898 (Ethanol)	DB00898 (Ethanol)
Leishmania major.	Q4Q6X9	S-adenosylmethionine decarboxylase proenzyme (EC 4.1.1.50).	LmjF30.3110.1.pdb	1jl0A (LYASE)	30	1.29	1i72B (LYASE)	100	80	76.92	100	MAO				DB00194 (Vidarabine)
Leishmania major.	Q4Q6X9	S-adenosylmethionine decarboxylase proenzyme (EC 4.1.1.50).	LmjF30.3110.1.pdb	1jl0A (LYASE)	30	1.29	1i79B (LYASE)	100	83.33	78.57	100	MHZ				DB00194 (Vidarabine)
Leishmania major.	Q4Q7L5	Adenosine kinase, putative (EC 2.7.1.20).	LmjF30.0880.2.pdb	1bx4A (TRANSFERASE)	41	1.65	1bx4A (TRANSFERASE)	100	100	82.35	100	ADN			DB00194 (Vidarabine)	DB00242 (Cladribine) DB00194 (Vidarabine)
Leishmania major.	Q4Q8T8	Serine/threonine protein phosphatase, putative (EC 3.1.3.16).	LmjF26.2530.1.pdb	1auiA (HYDROLASE)	53	1.46	1tcoA (COMPLEX (HYDROLASE/ISOMERASE))	100	100	100	100	FK5	DB00864 (Tacrolimus)	DB00864 (Tacrolimus)	DB00864 (Tacrolimus)	DB00864 (Tacrolimus) DB00337 (Pimecrolimus)
Leishmania major.	Q4QAC7	3-oxoacyl-(Acyl-carrier protein) reductase, putative.	LmjF24.2030.2.pdb	1e6wA (DEHYDROGENASE)	49	1.48	1e6wC (DEHYDROGENASE)	100	100	75	100	EST	DB00783 (Estradiol)	DB00783 (Estradiol)	DB04573 (Estriol) DB00783 (Estradiol)	DB04573 (Estriol) DB00977 (Ethinyl Estradiol) DB00783 (Estradiol)
Leishmania major.	Q4QAJ4	Histone deacetylase, putative.	LmjF24.1370.2.pdb	1t64A (HYDROLASE)	36	1.14	1c3sA (LYASE)	83.33	80	90.91	100	SHH	DB02546 (Vorinostat)	DB02546 (Vorinostat)	DB02546 (Vorinostat)	DB02546 (Vorinostat)
Leishmania major.	Q4QAP8	Triosephosphate isomerase (EC 5.3.1.1).	LmjF24.0850.3.pdb	1kv5A (ISOMERASE)	69	2.02	1tpfA (ISOMERASE(INTRAMOLECULAR)	100	100	91.67	100	DMS	DB01093 (Dimethyl sulfoxide)	DB01093 (Dimethyl sulfoxide)	DB01093 (Dimethyl sulfoxide)	DB01093 (Dimethyl sulfoxide)
Leishmania major.	Q4QBC0	Acetyl-CoA synthetase, putative (EC 6.2.1.1).	LmjF23.0540.2.pdb	1pg4A (LIGASE)	54	1.66	1pg4B (LIGASE)	100	100	90.48	100	PRX		DB00194 (Vidarabine)		DB01073 (Fludarabine) DB00194 (Vidarabine)
Leishmania major.	Q4QBK4	Peptidyl-prolyl cis-trans isomerase (EC 5.2.1.8).	LmjF22.1430.1.pdb	1r9hA (ISOMERASE)	42	1.55	1pbk_ (ISOMERASE)	80	100	78.57	100	RAP			DB01590 (Everolimus)	DB01590 (Everolimus)
Leishmania major.	Q4QBK4	Peptidyl-prolyl cis-trans isomerase (EC 5.2.1.8).	LmjF22.1430.3.pdb	1yat_ (BINDING PROTEIN)	56	1.76	1d7hB (ISOMERASE)	87.5	100	87.5	100	FK5	DB00864 (Tacrolimus)	DB00864 (Tacrolimus)	DB00864 (Tacrolimus)	DB00864 (Tacrolimus) DB00337 (Pimecrolimus)
Leishmania major.	Q4QBK4	Peptidyl-prolyl cis-trans isomerase (EC 5.2.1.8).	LmjF22.1430.3.pdb	1yat_ (BINDING PROTEIN)	56	1.76	1d7hB (ISOMERASE)	87.5	100	86.67	100	818				DB00864 (Tacrolimus) DB00337 (Pimecrolimus)
Leishmania major.	Q4QBK4	Peptidyl-prolyl cis-trans isomerase (EC 5.2.1.8).	LmjF22.1430.3.pdb	1yat_ (BINDING PROTEIN)	56	1.76	1d7hB (ISOMERASE)	87.5	100	85.71	100	DMS	DB01093 (Dimethyl sulfoxide)	DB01093 (Dimethyl sulfoxide)	DB01093 (Dimethyl sulfoxide)	DB01093 (Dimethyl sulfoxide)
Leishmania major.	Q4QC21	Calcineurin B subunit, putative.	LmjF21.1630.3.pdb	1auiB (HYDROLASE)	46	1.57	1tcoB (COMPLEX (HYDROLASE/ISOMERASE))	100	100	80	100	FK5	DB00864 (Tacrolimus)	DB00864 (Tacrolimus)	DB00864 (Tacrolimus)	DB00864 (Tacrolimus) DB00337 (Pimecrolimus)
Leishmania major.	Q4QCE7	Histone deacetylase, putative.	LmjF21.0680.1.pdb	1t64A (HYDROLASE)	36	1.47	1c3sA (LYASE)	83.33	80	90.91	100	SHH	DB02546 (Vorinostat)	DB02546 (Vorinostat)	DB02546 (Vorinostat)	DB02546 (Vorinostat)
Leishmania major.	Q4QD53	Inosine-5'-monophosphate dehydrogenase (EC 1.1.1.205).	LmjF19.1560.1.pdb	1jr1A (OXIDOREDUCTASE)	58	1.51	1jr1B (OXIDOREDUCTASE)	100	100	100	100	MOA	DB01024 (Mycophenolic acid)	DB01024 (Mycophenolic acid)	DB00688 (Mycophenolate mofetil) DB01024 (Mycophenolic acid)	DB00688 (Mycophenolate mofetil) DB01024 (Mycophenolic acid)
Leishmania major.	Q4QED0	Putative uncharacterized protein.	LmjF17.0780.3.pdb	1hbkA (FATTY ACID METABOLISM)	36	1.41	1hbkA (FATTY ACID METABOLISM)	100	100	75	100	MYR				DB00313 (Valproic Acid) DB00548 (Azelaic Acid)
Leishmania major.	Q4QEH4	Peptidase T, putative (Peptidase t, putative, metallo-peptidase,clanmh, family m20b) (EC 3.4.11.14).	LmjF17.0140.2.pdb	1vixA (HYDROLASE)	40	1.57	1fnoA (HYDROLASE)	100	100	85.71	100	ZN2		DB01593 (Zinc)		DB01593 (Zinc)
Leishmania major.	Q4QFG6	Serine/threonine-protein kinase, putative (Protein kinase, putative).	LmjF15.0180.2.pdb	2eu9A (TRANSFERASE)	35	1.08	1q97B (TRANSFERASE)	76.92	92.86	83.33	100	ADN			DB00194 (Vidarabine)	DB00242 (Cladribine) DB00194 (Vidarabine)
Leishmania major.	Q4QFL8	Enolase (EC 4.2.1.11).	LmjF14.1160.2.pdb	2akzA (LYASE)	61	1.79	2oneA (LYASE)	100	100	100	100	LI	DB01356 (Lithium)	DB01356 (Lithium)	DB01356 (Lithium)	DB01356 (Lithium)
Leishmania major.	Q4QGN9	Glucose-6-phosphate isomerase (EC 5.3.1.9).	LmjF12.0530.2.pdb	1t10A (ISOMERASE)	95	2.18	1iatA (ISOMERASE)	100	100	100	100	SUL			DB00653 (Magnesium Sulfate)	DB00653 (Magnesium Sulfate)
Leishmania major.	Q4QHC5	FKBP-type peptidyl-prolyl cis-trans isomerase, putative (EC 5.2.1.8).	LmjF10.0890.2.pdb	1fd9A (ISOMERASE)	45	1.36	1yat_ (BINDING PROTEIN)	75	100	80	100	FK5	DB00864 (Tacrolimus)	DB00864 (Tacrolimus)	DB00864 (Tacrolimus)	DB00864 (Tacrolimus) DB00337 (Pimecrolimus)
Leishmania major.	Q4QHC5	FKBP-type peptidyl-prolyl cis-trans isomerase, putative (EC 5.2.1.8).	LmjF10.0890.2.pdb	1fd9A (ISOMERASE)	45	1.36	1d7hB (ISOMERASE)	75	100	100	100	DMS	DB01093 (Dimethyl sulfoxide)	DB01093 (Dimethyl sulfoxide)	DB01093 (Dimethyl sulfoxide)	DB01093 (Dimethyl sulfoxide)
Leishmania major.	Q4QHG4	Glycerol-3-phosphate dehydrogenase [NAD+], glycosomal (EC 1.1.1.8).	LmjF10.0510.1.pdb	1evyA (OXIDOREDUCTASE)	89	2.11	1m66A (OXIDOREDUCTASE)	95	100	89.47	100	PLM				DB00313 (Valproic Acid) DB00548 (Azelaic Acid)

Organism	UniProt Id	Target description	3D model id	PDB Template	Model sequence identity	ModPipe Quality Score	PDB Expansion id	Binding site expansion sequence identity	Binding site expansion structure identity	Binding site sequence identity	Binding site structure identity	PDB Ligand id	Drugbank (Exact)	Drugbank (supra-structure)	Drugbank (sub-structure)	Drugbank (similar)
Leishmania major.	Q4QHT2	Calmodulin, putative.	LmjF09.0910CU.1.pdb	1qtxA (SIGNALING PROTEIN)	91	2.07	1lin_ (CALCIUM-BINDING PROTEIN)	100	100	93.94	100	TFP	DB00831 (Trifluoperazine)	DB00831 (Trifluoperazine) DB00508 (Triflupromazine)	DB00831 (Trifluoperazine) DB00623 (Fluphenazine)	DB00831 (Trifluoperazine) DB00508 (Triflupromazine) DB00623 (Fluphenazine)
Leishmania major.	Q4QHT2	Calmodulin, putative.	LmjF09.0910CU.1.pdb	1qtxA (SIGNALING PROTEIN)	91	2.07	1dtlA (STRUCTURAL PROTEIN)	82.35	89.47	93.75	100	BEP	DB01244 (Bepridil)	DB01244 (Bepridil)	DB01244 (Bepridil)	DB01244 (Bepridil)
Leishmania major.	Q4QHT2	Calmodulin, putative.	LmjF09.0910CU.1.pdb	1qtxA (SIGNALING PROTEIN)	91	2.07	1l7zA (METAL BINDING PROTEIN/PROTEIN BINDING)	100	100	100	100	MYR				DB00313 (Valproic Acid) DB00548 (Azelaic Acid)
Leishmania major.	Q4QI56	Phosphoribosylpyrophosphate synthetase (EC 2.7.6.1).	LmjF08.1130.2.pdb	1dkuA (TRANSFERASE)	44	1.47	1dkuA (TRANSFERASE)	100	100	75	100	ABM				DB01073 (Fludarabine)
Leishmania major.	Q4QIJ7	Serine/threonine kinase, putative (Protein kinase, putative).	LmjF07.0900.2.pdb	2h6dA (SIGNALING PROTEIN,TRANSFERASE)	44	1.51	1fmoE (COMPLEX (PHOSPHOTRANSFERASE/INHIBITOR))	82.35	89.47	75	100	ADN			DB00194 (Vidarabine)	DB00242 (Cladribine) DB00194 (Vidarabine)
Leishmania major.	Q4QIK9	Flavoprotein subunit-like protein.	LmjF07.0800.2.pdb	1y0pA (OXIDOREDUCTASE)	37	1.1	1d4cD (OXIDOREDUCTASE)	100	100	90.91	100	SUL			DB00653 (Magnesium Sulfate)	DB00653 (Magnesium Sulfate)
Leishmania major.	Q4QIM0	Centrin, putative (Caltractin, putative).	LmjF07.0710.3.pdb	1qx2A (SIGNALING PROTEIN)	51	1.12	1lin_ (CALCIUM-BINDING PROTEIN)	80	100	77.78	100	TFP	DB00831 (Trifluoperazine)	DB00831 (Trifluoperazine) DB00508 (Triflupromazine)	DB00831 (Trifluoperazine) DB00623 (Fluphenazine)	DB00831 (Trifluoperazine) DB00508 (Triflupromazine) DB00623 (Fluphenazine)
Mycobacterium leprae.	Q50192	Leucyl-tRNA synthetase (EC 6.1.1.4) (Leucine--tRNA ligase) (LeuRS).	13092440.1.pdb	1h3nA (AMINOACYL-TRNA SYNTHETASE)	42	1.22	1obcA (SYNTHETASE)	100	100	86.67	100	2AD				DB00194 (Vidarabine)
Mycobacterium tuberculosis.	Q50649	Aspartyl-tRNA synthetase (EC 6.1.1.12) (Aspartate--tRNA ligase)(AspRS).	15609709.2.pdb	1c0aA (LIGASE/RNA)	46	1.47	1il2B (LIGASE/RNA)	100	100	90.62	100	AMO				DB01073 (Fludarabine)
Trypanosoma brucei.	Q57TY7	Iron/ascorbate oxidoreductase family protein, putative (EC 1.2.3.4).	Tb927.7.7500.2.pdb	1w9yA (OXYGENASE)	27	1.38	1blz_ (B-LACTAM ANTIBIOTIC)	83.33	100	100	100	NO	DB00435 (Nitric Oxide)	DB00435 (Nitric Oxide)	DB00435 (Nitric Oxide)	DB00435 (Nitric Oxide)
Trypanosoma brucei.	Q57VP6	Centrin, putative.	Tb927.8.1080.3.pdb	1qx2A (SIGNALING PROTEIN)	48	1.07	1lin_ (CALCIUM-BINDING PROTEIN)	80	100	77.78	100	TFP	DB00831 (Trifluoperazine)	DB00831 (Trifluoperazine) DB00508 (Triflupromazine)	DB00831 (Trifluoperazine) DB00623 (Fluphenazine)	DB00831 (Trifluoperazine) DB00508 (Triflupromazine) DB00623 (Fluphenazine)
Trypanosoma brucei.	Q57W80	Mannosyl-oligosaccharide 1,2-alpha-mannosidase IB, putative.	Tb927.8.2930.2.pdb	1x9dA (HYDROLASE)	31	1.05	1kreB (HYDROLASE)	100	100	100	100	DMJ			DB00491 (Miglitol) DB00419 (Miglustat)	DB00491 (Miglitol) DB00419 (Miglustat)
Trypanosoma brucei.	Q57W81	Mannosyl-oligosaccharide 1,2-alpha-mannosidase IB, putative.	Tb927.8.2920.2.pdb	1hcuA (GLYCOSYLATION)	30	1.06	1kreB (HYDROLASE)	100	100	100	100	DMJ			DB00491 (Miglitol) DB00419 (Miglustat)	DB00491 (Miglitol) DB00419 (Miglustat)
Trypanosoma brucei.	Q57W82	Mannosyl-oligosaccharide 1,2-alpha-mannosidase IB, putative.	Tb927.8.2910.3.pdb	1x9dA (HYDROLASE)	29	1.08	1kreB (HYDROLASE)	100	100	100	100	DMJ			DB00491 (Miglitol) DB00419 (Miglustat)	DB00491 (Miglitol) DB00419 (Miglustat)
Trypanosoma brucei.	Q57WF6	Centrin, putative.	Tb927.7.3410.3.pdb	1qx2A (SIGNALING PROTEIN)	50	1.16	1lin_ (CALCIUM-BINDING PROTEIN)	80	100	77.78	100	TFP	DB00831 (Trifluoperazine)	DB00831 (Trifluoperazine) DB00508 (Triflupromazine)	DB00831 (Trifluoperazine) DB00623 (Fluphenazine)	DB00831 (Trifluoperazine) DB00508 (Triflupromazine) DB00623 (Fluphenazine)
Trypanosoma brucei.	Q57WF7	Peptidyl-prolyl cis-trans isomerase, putative (EC 5.2.1.8).	Tb927.7.3420.1.pdb	1jvwA (ISOMERASE)	53	1.73	1fkd_ (CIS-TRANS ISOMERASE)	81.25	100	80	100	818				DB00864 (Tacrolimus) DB00337 (Pimecrolimus)
Trypanosoma brucei.	Q57WF7	Peptidyl-prolyl cis-trans isomerase, putative (EC 5.2.1.8).	Tb927.7.3420.3.pdb	1yat_ (BINDING PROTEIN)	49	1.71	1fkd_ (CIS-TRANS ISOMERASE)	81.25	100	85.71	100	DMS	DB01093 (Dimethyl sulfoxide)	DB01093 (Dimethyl sulfoxide)	DB01093 (Dimethyl sulfoxide)	DB01093 (Dimethyl sulfoxide)
Trypanosoma brucei.	Q57WF7	Peptidyl-prolyl cis-trans isomerase, putative (EC 5.2.1.8).	Tb927.7.3420.1.pdb	1jvwA (ISOMERASE)	53	1.73	1tcoC (COMPLEX (HYDROLASE/ISOMERASE))	80	100	75	100	FK5	DB00864 (Tacrolimus)	DB00864 (Tacrolimus)	DB00864 (Tacrolimus)	DB00864 (Tacrolimus) DB00337 (Pimecrolimus)
Trypanosoma brucei.	Q57XD7	Acetyl-CoA synthetase, putative (EC 6.2.1.1).	Tb927.8.2520.2.pdb	1pg4A (LIGASE)	53	1.67	1pg4B (LIGASE)	100	100	90.48	100	PRX		DB00194 (Vidarabine)		DB01073 (Fludarabine) DB00194 (Vidarabine)
Trypanosoma brucei.	Q57XH4	Iron/ascorbate oxidoreductase family protein, putative (EC 1.2.3.4).	Tb927.5.300.1.pdb	1odmA (OXIDOREDUCTASE)	29	1.37	1blz_ (B-LACTAM ANTIBIOTIC)	100	100	100	100	NO	DB00435 (Nitric Oxide)	DB00435 (Nitric Oxide)	DB00435 (Nitric Oxide)	DB00435 (Nitric Oxide)
Trypanosoma brucei.	Q57ZL6	Inosine-adenosine-guanosine-nucleoside hydrolase (EC 3.2.2.1).	Tb927.3.2960.1.pdb	1hozA (HYDROLASE)	68	1.96	1kicB (HYDROLASE)	100	100	88.89	100	NOS			DB01280 (Nelarabine)	DB01280 (Nelarabine)
Trypanosoma brucei.	Q582G3	Dihydrofolate reductase-thymidylate synthase (EC 1.5.1.3).	Tb927.7.5480.2.pdb	1f28A (TRANSFERASE)	60	1.37	1ju6D (TRANSFERASE)	94.12	100	89.47	100	LYA	DB00642 (Pemetrexed)	DB00642 (Pemetrexed)	DB00642 (Pemetrexed)	DB00642 (Pemetrexed)
Trypanosoma brucei.	Q582G3	Dihydrofolate reductase-thymidylate synthase (EC 1.5.1.3).	Tb927.7.5480.2.pdb	1f28A (TRANSFERASE)	60	1.37	1dduB (METHYLTRANSFERASE)	100	100	100	100	DDU			DB00322 (Floxuridine) DB00432 (Trifluridine)	DB00322 (Floxuridine) DB00432 (Trifluridine)
Trypanosoma brucei.	Q582G3	Dihydrofolate reductase-thymidylate synthase (EC 1.5.1.3).	Tb927.7.5480.2.pdb	1f28A (TRANSFERASE)	60	1.37	2tsrD (METHYLTRANSFERASE)	100	100	84.62	100	D16	DB00293 (Raltitrexed)	DB00293 (Raltitrexed)	DB00293 (Raltitrexed)	DB00293 (Raltitrexed)
Trypanosoma brucei.	Q582G3	Dihydrofolate reductase-thymidylate synthase (EC 1.5.1.3).	Tb927.7.5480.2.pdb	1f28A (TRANSFERASE)	60	1.37	1f4fA (TRANSFERASE)	100	100	100	100	SUL			DB00653 (Magnesium Sulfate)	DB00653 (Magnesium Sulfate)
Trypanosoma brucei.	Q582G3	Dihydrofolate reductase-thymidylate synthase (EC 1.5.1.3).	Tb927.7.5480.2.pdb	1f28A (TRANSFERASE)	60	1.37	1tduA (METHYLTRANSFERASE)	94.12	100	94.12	100	DUR			DB00322 (Floxuridine) DB00432 (Trifluridine)	DB00322 (Floxuridine) DB00432 (Trifluridine)
Trypanosoma brucei.	Q582G3	Dihydrofolate reductase-thymidylate synthase (EC 1.5.1.3).	Tb927.7.5480.2.pdb	1f28A (TRANSFERASE)	60	1.37	1jutB (TRANSFERASE)	80	100	85.71	100	LYD				DB00642 (Pemetrexed)
Trypanosoma brucei.	Q582G3	Dihydrofolate reductase-thymidylate synthase (EC 1.5.1.3).	Tb927.7.5480.2.pdb	1f28A (TRANSFERASE)	60	1.37	2tdd_ (TRANSFERASE (METHYLTRANFERASE))	93.33	100	95.45	100	UFP		DB00322 (Floxuridine)		DB00322 (Floxuridine)
Trypanosoma brucei.	Q582G3	Dihydrofolate reductase-thymidylate synthase (EC 1.5.1.3).	Tb927.7.5480.1.pdb	1qzfA (OXIDOREDUCTASE, TRANSFERASE)	43	1.28	1f4fB (TRANSFERASE)	100	100	100	100	SUL			DB00653 (Magnesium Sulfate)	DB00653 (Magnesium Sulfate)
Trypanosoma brucei.	Q584S0	Adenosine kinase, putative (EC 2.7.1.20).	Tb927.6.2360.2.pdb	1bx4A (TRANSFERASE)	41	1.61	1bx4A (TRANSFERASE)	100	100	76.47	100	ADN			DB00194 (Vidarabine)	DB00242 (Cladribine) DB00194 (Vidarabine)
Trypanosoma brucei.	Q586M6	Iron/ascorbate oxidoreductase family protein, putative.	Tb927.2.6310.2.pdb	1gp6A (OXYGENASE)	29	1.39	1bx4A (TRANSFERASE)	100	100	100	100	NO	DB00435 (Nitric Oxide)	DB00435 (Nitric Oxide)	DB00435 (Nitric Oxide)	DB00435 (Nitric Oxide)
Trypanosoma brucei.	Q586M8	Iron/ascorbate oxidoreductase family protein, putative.	Tb927.2.6270.2.pdb	1gp6A (OXYGENASE)	29	1.4	1bx4A (TRANSFERASE)	100	100	100	100	NO	DB00435 (Nitric Oxide)	DB00435 (Nitric Oxide)	DB00435 (Nitric Oxide)	DB00435 (Nitric Oxide)
Trypanosoma brucei.	Q586N0	Iron/ascorbate oxidoreductase family protein, putative.	Tb927.2.6230.2.pdb	1gp6A (OXYGENASE)	30	1.42	1bx4A (TRANSFERASE)	100	100	100	100	NO	DB00435 (Nitric Oxide)	DB00435 (Nitric Oxide)	DB00435 (Nitric Oxide)	DB00435 (Nitric Oxide)
Trypanosoma brucei.	Q586N2	Iron/ascorbate oxidoreductase family protein, putative.	Tb927.2.6210.2.pdb	1gp6A (OXYGENASE)	30	1.4	1bx4A (TRANSFERASE)	100	100	100	100	NO	DB00435 (Nitric Oxide)	DB00435 (Nitric Oxide)	DB00435 (Nitric Oxide)	DB00435 (Nitric Oxide)
Trypanosoma brucei.	Q586N4	Iron/ascorbate oxidoreductase family protein, putative.	Tb927.2.6180.1.pdb	1odmA (OXIDOREDUCTASE)	30	1.41	1blz_ (B-LACTAM ANTIBIOTIC)	100	100	100	100	NO	DB00435 (Nitric Oxide)	DB00435 (Nitric Oxide)	DB00435 (Nitric Oxide)	DB00435 (Nitric Oxide)
Trypanosoma brucei.	Q587C0	3-hydroxy-3-methylglutaryl-CoA reductase, putative (EC 1.1.1.34).	Tb927.6.4540.1.pdb	1dqaA (OXIDOREDUCTASE)	50	1.61	1hwkD (OXIDOREDUCTASE)	100	100	80	100	117	DB01076 (Atorvastatin)	DB01076 (Atorvastatin)	DB01076 (Atorvastatin)	DB01076 (Atorvastatin)
Toxoplasma gondii.	Q58G65	CAMP-dependent protein kinase catalytic subunit isoform 2.	42.m00079.2.pdb	1rdqE (TRANSFERASE/TRANSFERASE INHIBITOR)	48	1.66	1fmoE (COMPLEX (PHOSPHOTRANSFERASE/INHIBITOR))	100	100	100	100	ADN			DB00194 (Vidarabine)	DB00242 (Cladribine) DB00194 (Vidarabine)
Mycobacterium leprae.	Q59533	DNA gyrase subunit B (EC 5.99.1.3).	13092417.1.pdb	1ei1A (ISOMERASE)	48	1.16	1aj6_ (TOPOISOMERASE)	92.86	100	80	100	NOV				DB01051 (Novobiocin)
Cryptosporidium hominis.	Q5CEK2	Serine/threonine kinase-1.	Chro.50371.2.pdb	2eu9A (TRANSFERASE)	41	1.08	1q97B (TRANSFERASE)	76.92	92.86	83.33	100	ADN			DB00194 (Vidarabine)	DB00242 (Cladribine) DB00194 (Vidarabine)
Cryptosporidium hominis.	Q5CGA3	Chain A, crystal structure of Dhfr.	Chro.40506.2.pdb	2tsrA (METHYLTRANSFERASE)	56	1.38	1axwB (METHYLTRANSFERASE)	76.47	77.27	81.25	100	MTX	DB00563 (Methotrexate)	DB00563 (Methotrexate)	DB00563 (Methotrexate)	DB00563 (Methotrexate)
Cryptosporidium hominis.	Q5CGA3	Chain A, crystal structure of Dhfr.	Chro.40506.1.pdb	1qzfA (OXIDOREDUCTASE, TRANSFERASE)	100	2.16	1jutB (TRANSFERASE)	86.67	100	100	100	LYD				DB00642 (Pemetrexed)
Cryptosporidium hominis.	Q5CGA3	Chain A, crystal structure of Dhfr.	Chro.40506.1.pdb	1qzfA (OXIDOREDUCTASE, TRANSFERASE)	100	2.16	1dduB (METHYLTRANSFERASE)	100	100	100	100	DDU			DB00322 (Floxuridine) DB00432 (Trifluridine)	DB00322 (Floxuridine) DB00432 (Trifluridine)
Cryptosporidium hominis.	Q5CGA3	Chain A, crystal structure of Dhfr.	Chro.40506.1.pdb	1qzfA (OXIDOREDUCTASE, TRANSFERASE)	100	2.16	1tduA (METHYLTRANSFERASE)	94.12	100	100	100	DUR			DB00322 (Floxuridine) DB00432 (Trifluridine)	DB00322 (Floxuridine) DB00432 (Trifluridine)
Cryptosporidium hominis.	Q5CGA3	Chain A, crystal structure of Dhfr.	Chro.40506.1.pdb	1qzfA (OXIDOREDUCTASE, TRANSFERASE)	100	2.16	1axwA (METHYLTRANSFERASE)	78.95	95	100	100	MTX	DB00563 (Methotrexate)	DB00563 (Methotrexate)	DB00563 (Methotrexate)	DB00563 (Methotrexate)
Cryptosporidium hominis.	Q5CGA3	Chain A, crystal structure of Dhfr.	Chro.40506.1.pdb	1qzfA (OXIDOREDUCTASE, TRANSFERASE)	100	2.16	1jtuA (TRANSFERASE)	78.95	100	100	100	LYB				DB00642 (Pemetrexed)
Cryptosporidium hominis.	Q5CGA3	Chain A, crystal structure of Dhfr.	Chro.40506.2.pdb	2tsrA (METHYLTRANSFERASE)	56	1.38	1dduA (METHYLTRANSFERASE)	100	100	84.62	100	DDU			DB00322 (Floxuridine) DB00432 (Trifluridine)	DB00322 (Floxuridine) DB00432 (Trifluridine)
Cryptosporidium hominis.	Q5CGA3	Chain A, crystal structure of Dhfr.	Chro.40506.1.pdb	1qzfA (OXIDOREDUCTASE, TRANSFERASE)	100	2.16	2tdd_ (TRANSFERASE (METHYLTRANFERASE))	93.33	100	100	100	UFP		DB00322 (Floxuridine)		DB00322 (Floxuridine)
Cryptosporidium hominis.	Q5CGA3	Chain A, crystal structure of Dhfr.	Chro.40506.1.pdb	1qzfA (OXIDOREDUCTASE, TRANSFERASE)	100	2.16	1ju6D (TRANSFERASE)	94.12	100	100	100	LYA	DB00642 (Pemetrexed)	DB00642 (Pemetrexed)	DB00642 (Pemetrexed)	DB00642 (Pemetrexed)
Cryptosporidium hominis.	Q5CGA3	Chain A, crystal structure of Dhfr.	Chro.40506.1.pdb	1qzfA (OXIDOREDUCTASE, TRANSFERASE)	100	2.16	1f4fB (TRANSFERASE)	100	100	100	100	SUL			DB00653 (Magnesium Sulfate)	DB00653 (Magnesium Sulfate)
Cryptosporidium hominis.	Q5CGA3	Chain A, crystal structure of Dhfr.	Chro.40506.1.pdb	1qzfA (OXIDOREDUCTASE, TRANSFERASE)	100	2.16	1hvyB (TRANSFERASE)	95	100	100	100	D16	DB00293 (Raltitrexed)	DB00293 (Raltitrexed)	DB00293 (Raltitrexed)	DB00293 (Raltitrexed)
Cryptosporidium hominis.	Q5CGA7	Centrin.	Chro.50390.3.pdb	1oqpA (PROTEIN BINDING)	65	1.34	1lin_ (CALCIUM-BINDING PROTEIN)	80	100	88.89	100	TFP	DB00831 (Trifluoperazine)	DB00831 (Trifluoperazine) DB00508 (Triflupromazine)	DB00831 (Trifluoperazine) DB00623 (Fluphenazine)	DB00831 (Trifluoperazine) DB00508 (Triflupromazine) DB00623 (Fluphenazine)
Cryptosporidium hominis.	Q5CGD3	Acetyl-CoenzymeA synthetase (Acetate--coa ligase) (Acyl-activatingenzyme).	Chro.10418.1.pdb	1pg4A (LIGASE)	48	1.56	1pg4B (LIGASE)	100	100	85.71	100	PRX		DB00194 (Vidarabine)		DB01073 (Fludarabine) DB00194 (Vidarabine)
Cryptosporidium hominis.	Q5CH50	Protein phosphatase 2b.	Chro.60481.1.pdb	1auiA (HYDROLASE)	53	1.17	1tcoA (COMPLEX (HYDROLASE/ISOMERASE))	100	100	100	100	FK5	DB00864 (Tacrolimus)	DB00864 (Tacrolimus)	DB00864 (Tacrolimus)	DB00864 (Tacrolimus) DB00337 (Pimecrolimus)
Cryptosporidium hominis.	Q5CH58	Protein phosphatase 2B regulatory subunit.	Chro.10159.2.pdb	1auiB (HYDROLASE)	45	1.51	1tcoB (COMPLEX (HYDROLASE/ISOMERASE))	100	100	80	80	FK5	DB00864 (Tacrolimus)	DB00864 (Tacrolimus)	DB00864 (Tacrolimus)	DB00864 (Tacrolimus) DB00337 (Pimecrolimus)
Cryptosporidium hominis.	Q5CHH5	Protein kinase (EC 2.7.1.37), cAMP-dependent, catalytic chain.	Chro.30344.2.pdb	1rdqE (TRANSFERASE/TRANSFERASE INHIBITOR)	55	1.6	1fmoE (COMPLEX (PHOSPHOTRANSFERASE/INHIBITOR))	100	100	94.44	100	ADN			DB00194 (Vidarabine)	DB00242 (Cladribine) DB00194 (Vidarabine)
Cryptosporidium hominis.	Q5CI16	Adenosylhomocysteinase (EC 3.3.1.1).	Chro.30017.1.pdb	1v8bA (HYDROLASE)	53	1.52	1ky5D (HYDROLASE)	87.5	100	91.3	100	ADY				DB00194 (Vidarabine)
Cryptosporidium hominis.	Q5CI16	Adenosylhomocysteinase (EC 3.3.1.1).	Chro.30017.1.pdb	1v8bA (HYDROLASE)	53	1.52	1d4fB (HYDROLASE)	91.3	100	91.3	100	ADN			DB00194 (Vidarabine)	DB00242 (Cladribine) DB00194 (Vidarabine)
Cryptosporidium hominis.	Q5CIZ1	Putative uncharacterized protein.	Chro.20360.2.pdb	1mr3F (SIGNALING PROTEIN)	73	2.02	1mr3F (SIGNALING PROTEIN)	100	100	76.92	100	EOH	DB00898 (Ethanol)	DB00898 (Ethanol)	DB00898 (Ethanol)	DB00898 (Ethanol)
Cryptosporidium hominis.	Q5CJ45	ADP ribosylation factor 1.	Chro.30115.2.pdb	1mr3F (SIGNALING PROTEIN)	77	2.08	1mr3F (SIGNALING PROTEIN)	100	100	84.62	100	EOH	DB00898 (Ethanol)	DB00898 (Ethanol)	DB00898 (Ethanol)	DB00898 (Ethanol)
Cryptosporidium hominis.	Q5CK89	Glucose-6-phosphate isomerase (EC 5.3.1.9).	Chro.20336.1.pdb	2cxnA (ISOMERASE)	40	1.54	1iatA (ISOMERASE)	100	100	87.5	100	SUL			DB00653 (Magnesium Sulfate)	DB00653 (Magnesium Sulfate)
Cryptosporidium hominis.	Q5CL03	Centrin.	Chro.30159.3.pdb	1oqpA (PROTEIN BINDING)	68	1.35	1lin_ (CALCIUM-BINDING PROTEIN)	80	100	100	100	TFP	DB00831 (Trifluoperazine)	DB00831 (Trifluoperazine) DB00508 (Triflupromazine)	DB00831 (Trifluoperazine) DB00623 (Fluphenazine)	DB00831 (Trifluoperazine) DB00508 (Triflupromazine) DB00623 (Fluphenazine)
Cryptosporidium hominis.	Q5CLG3	Nucleoside diphosphate kinase (EC 2.7.4.6).	Chro.50237.3.pdb	1wkjA (TRANSFERASE)	46	1.38	1nhkR (PHOSPHOTRANSFERASE)	92.31	100	75	100	CMP				DB01073 (Fludarabine)
Cryptosporidium hominis.	Q5CLI0	Histone deacetylase.	Chro.60019.1.pdb	1t64A (HYDROLASE)	40	1.5	1c3sA (LYASE)	83.33	80	90.91	100	SHH	DB02546 (Vorinostat)	DB02546 (Vorinostat)	DB02546 (Vorinostat)	DB02546 (Vorinostat)
Cryptosporidium hominis.	Q5CLR8	Calmodulin.	Chro.20092.2.pdb	1rfjA (METAL BINDING PROTEIN)	87	2.07	1cll_ (CALCIUM-BINDING PROTEIN)	83.33	100	100	100	EOH	DB00898 (Ethanol)	DB00898 (Ethanol)	DB00898 (Ethanol)	DB00898 (Ethanol)
Cryptosporidium hominis.	Q5CLR8	Calmodulin.	Chro.20092.3.pdb	2amiA (CELL CYCLE)	58	1.38	1lin_ (CALCIUM-BINDING PROTEIN)	90	100	77.78	100	TFP	DB00831 (Trifluoperazine)	DB00831 (Trifluoperazine) DB00508 (Triflupromazine)	DB00831 (Trifluoperazine) DB00623 (Fluphenazine)	DB00831 (Trifluoperazine) DB00508 (Triflupromazine) DB00623 (Fluphenazine)
Cryptosporidium hominis.	Q5CM00	Nucleoside diphosphate kinase.	Chro.40219.1.pdb	1npk_ (PHOSPHOTRANSFERASE(PO4 AS ACCEPTOR))	51	1.69	1nhkR (PHOSPHOTRANSFERASE)	100	100	91.67	100	CMP				DB01073 (Fludarabine)
Cryptosporidium hominis.	Q5CMF9	Triosephosphate isomerase (EC 5.3.1.1).	Chro.10337.1.pdb	1kv5A (ISOMERASE)	45	1.59	1tpfA (ISOMERASE(INTRAMOLECULAR)	100	100	83.33	100	DMS	DB01093 (Dimethyl sulfoxide)	DB01093 (Dimethyl sulfoxide)	DB01093 (Dimethyl sulfoxide)	DB01093 (Dimethyl sulfoxide)
Cryptosporidium hominis.	Q5CMT9	Histone deacetylase.	Chro.60173.1.pdb	1t64A (HYDROLASE)	37	1.48	1c3sA (LYASE)	83.33	80	90.91	100	SHH	DB02546 (Vorinostat)	DB02546 (Vorinostat)	DB02546 (Vorinostat)	DB02546 (Vorinostat)
Cryptosporidium hominis.	Q5CN58	DNA topoisomerase I.	Chro.70373.3.pdb	2b9sA (ISOMERASE/DNA)	45	1.15	1k4tA (ISOMERASE/DNA)	100	75	80	100	TTG				DB01030 (Topotecan)
Cryptosporidium hominis.	Q5CN58	DNA topoisomerase I.	Chro.70373.3.pdb	2b9sA (ISOMERASE/DNA)	45	1.15	1k4tA (ISOMERASE/DNA)	100	75	100	100	TTC	DB01030 (Topotecan)	DB01030 (Topotecan)	DB01030 (Topotecan)	DB00762 (Irinotecan) DB01030 (Topotecan)
Cryptosporidium hominis.	Q5CNC3	Phosphorylase (EC 2.4.1.1).	Chro.60284.1.pdb	1ygpA (GLYCOSYLTRANSFERASE)	49	1.53	2ecpB (ACARBOSE)	100	100	100	100	ACR	DB00284 (Acarbose)	DB00284 (Acarbose)	DB00284 (Acarbose)	DB00284 (Acarbose)
Cryptosporidium parvum Iowa II.	Q5CPH1	Adenosylhomocysteinase (EC 3.3.1.1) (Fragment).	cgd3_80.1.pdb	1v8bA (HYDROLASE)	53	1.5	1ky5D (HYDROLASE)	87.5	100	91.3	100	ADY				DB00194 (Vidarabine)
Cryptosporidium parvum Iowa II.	Q5CPH1	Adenosylhomocysteinase (EC 3.3.1.1) (Fragment).	cgd3_80.1.pdb	1v8bA (HYDROLASE)	53	1.5	1d4fB (HYDROLASE)	91.3	100	91.3	100	ADN			DB00194 (Vidarabine)	DB00242 (Cladribine) DB00194 (Vidarabine)
Cryptosporidium parvum Iowa II.	Q5CPK1	RPD3/HD1 histone deacetylase (Fragment).	cgd6_80.2.pdb	1t64A (HYDROLASE)	40	1.47	1c3sA (LYASE)	83.33	80	90.91	100	SHH	DB02546 (Vorinostat)	DB02546 (Vorinostat)	DB02546 (Vorinostat)	DB02546 (Vorinostat)
Cryptosporidium parvum Iowa II.	Q5CPK7	Inosine-5-monophosphate dehydrogenase (EC 1.1.1.205) (Fragment).	cgd6_20.3.pdb	2cu0A (OXIDOREDUCTASE)	54	1.53	1me7A (OXIDOREDUCTASE)	78.57	100	85.19	100	RVP		DB00811 (Ribavirin)		DB00811 (Ribavirin)
Cryptosporidium parvum Iowa II.	Q5CPQ4	Dihydrofolate reductase-thymidylate synthase (Fragment).	cgd4_4460.1.pdb	1qzfA (OXIDOREDUCTASE, TRANSFERASE)	98	2.11	1jutB (TRANSFERASE)	86.67	100	100	100	LYD				DB00642 (Pemetrexed)
Cryptosporidium parvum Iowa II.	Q5CPQ4	Dihydrofolate reductase-thymidylate synthase (Fragment).	cgd4_4460.1.pdb	1qzfA (OXIDOREDUCTASE, TRANSFERASE)	98	2.11	1dduB (METHYLTRANSFERASE)	100	100	100	100	DDU			DB00322 (Floxuridine) DB00432 (Trifluridine)	DB00322 (Floxuridine) DB00432 (Trifluridine)
Cryptosporidium parvum Iowa II.	Q5CPQ4	Dihydrofolate reductase-thymidylate synthase (Fragment).	cgd4_4460.1.pdb	1qzfA (OXIDOREDUCTASE, TRANSFERASE)	98	2.11	1tduA (METHYLTRANSFERASE)	94.12	100	100	100	DUR			DB00322 (Floxuridine) DB00432 (Trifluridine)	DB00322 (Floxuridine) DB00432 (Trifluridine)
Cryptosporidium parvum Iowa II.	Q5CPQ4	Dihydrofolate reductase-thymidylate synthase (Fragment).	cgd4_4460.1.pdb	1qzfA (OXIDOREDUCTASE, TRANSFERASE)	98	2.11	1f4fB (TRANSFERASE)	100	100	100	100	SUL			DB00653 (Magnesium Sulfate)	DB00653 (Magnesium Sulfate)
Cryptosporidium parvum Iowa II.	Q5CPQ4	Dihydrofolate reductase-thymidylate synthase (Fragment).	cgd4_4460.1.pdb	1qzfA (OXIDOREDUCTASE, TRANSFERASE)	98	2.11	1ju6D (TRANSFERASE)	94.12	100	100	100	LYA	DB00642 (Pemetrexed)	DB00642 (Pemetrexed)	DB00642 (Pemetrexed)	DB00642 (Pemetrexed)
Cryptosporidium parvum Iowa II.	Q5CPQ4	Dihydrofolate reductase-thymidylate synthase (Fragment).	cgd4_4460.1.pdb	1qzfA (OXIDOREDUCTASE, TRANSFERASE)	98	2.11	2tdd_ (TRANSFERASE (METHYLTRANFERASE))	93.33	100	100	100	UFP		DB00322 (Floxuridine)		DB00322 (Floxuridine)
Cryptosporidium parvum Iowa II.	Q5CPQ4	Dihydrofolate reductase-thymidylate synthase (Fragment).	cgd4_4460.1.pdb	1qzfA (OXIDOREDUCTASE, TRANSFERASE)	98	2.11	1jtuA (TRANSFERASE)	78.95	100	100	100	LYB				DB00642 (Pemetrexed)

Organism	UniProt Id	Target description	3D model id	PDB Template	Model sequence identity	ModPipe Quality Score	PDB Expansion id	Binding site expansion sequence identity	Binding site expansion structure identity	Binding site sequence identity	Binding site structure identity	PDB Ligand id	Drugbank (Exact)	Drugbank (supra-structure)	Drugbank (sub-structure)	Drugbank (similar)
Cryptosporidium parvum Iowa II.	Q5CPQ4	Dihydrofolate reductase-thymidylate synthase (Fragment).	cgd4_4460.1.pdb	1qzfA (OXIDOREDUCTASE, TRANSFERASE)	98	2.11	1hvyB (TRANSFERASE)	95	100	100	100	D16	DB00293 (Raltitrexed)	DB00293 (Raltitrexed)	DB00293 (Raltitrexed)	DB00293 (Raltitrexed)
Cryptosporidium parvum Iowa II.	Q5CPQ4	Dihydrofolate reductase-thymidylate synthase (Fragment).	cgd4_4460.1.pdb	1qzfA (OXIDOREDUCTASE, TRANSFERASE)	98	2.11	1axwA (METHYLTRANSFERASE)	78.95	95	100	100	MTX	DB00563 (Methotrexate)	DB00563 (Methotrexate)	DB00563 (Methotrexate)	DB00563 (Methotrexate)
Cryptosporidium parvum Iowa II.	Q5CR64	Putative nucleoside-diphosphate kinase (Fragment).	cgd4_1940.2.pdb	1wkjA (TRANSFERASE)	55	1.56	1nhkR (PHOSPHOTRANSFERASE)	92.31	100	83.33	100	CMP				DB01073 (Fludarabine)
Cryptosporidium parvum Iowa II.	Q5CRP8	Enolase (EC 4.2.1.11) (Fragment).	cgd5_1960.2.pdb	2akzA (LYASE)	62	1.76	2oneA (LYASE)	100	100	100	100	LI	DB01356 (Lithium)	DB01356 (Lithium)	DB01356 (Lithium)	DB01356 (Lithium)
Cryptosporidium parvum Iowa II.	Q5CRU2	Nucleoside diphosphate kinase (EC 2.7.4.6) (Fragment).	cgd5_1470.2.pdb	1wkjA (TRANSFERASE)	48	1.35	1nhkR (PHOSPHOTRANSFERASE)	92.31	100	75	100	CMP				DB01073 (Fludarabine)
Cryptosporidium parvum Iowa II.	Q5CS65	Centrin like protein with 4x EF hands.	cgd5_60.3.pdb	1oqpA (PROTEIN BINDING)	65	1.34	1lin_ (CALCIUM-BINDING PROTEIN)	80	100	88.89	100	TFP	DB00831 (Trifluoperazine)	DB00831 (Trifluoperazine) DB00508 (Triflupromazine)	DB00831 (Trifluoperazine) DB00623 (Fluphenazine)	DB00831 (Trifluoperazine) DB00508 (Triflupromazine) DB00623 (Fluphenazine)
Cryptosporidium parvum Iowa II.	Q5CS85	Acetyl-coenzyme A synthetase.	cgd1_3710.1.pdb	1pg4A (LIGASE)	48	1.57	1pg4B (LIGASE)	100	100	85.71	100	PRX		DB00194 (Vidarabine)		DB01073 (Fludarabine) DB00194 (Vidarabine)
Cryptosporidium parvum Iowa II.	Q5CSE7	Triosephosphate isomerase (EC 5.3.1.1).	cgd1_3040.1.pdb	1r2rA (ISOMERASE)	45	1.71	1tpfA (ISOMERASE(INTRAMOLECULAR)	100	100	75	100	DMS	DB01093 (Dimethyl sulfoxide)	DB01093 (Dimethyl sulfoxide)	DB01093 (Dimethyl sulfoxide)	DB01093 (Dimethyl sulfoxide)
Cryptosporidium parvum Iowa II.	Q5CTF8	Glucose-6-phosphate isomerase, cytosolic (GPI) (Phosphoglucoseisomerase) (PGI) (Phosphohexose isomerase) (PHI) (EC 5.3.1.9).	cgd2_3200.1.pdb	1t10A (ISOMERASE)	39	1.41	1iatA (ISOMERASE)	100	100	87.5	100	SUL			DB00653 (Magnesium Sulfate)	DB00653 (Magnesium Sulfate)
Cryptosporidium parvum Iowa II.	Q5CUZ6	ARF1/2 like small GTpase (Fragment).	cgd3_860.2.pdb	1mr3F (SIGNALING PROTEIN)	78	2.06	1mr3F (SIGNALING PROTEIN)	100	100	84.62	100	EOH	DB00898 (Ethanol)	DB00898 (Ethanol)	DB00898 (Ethanol)	DB00898 (Ethanol)
Cryptosporidium parvum Iowa II.	Q5CWQ1	Serine/threonine protein phosphatase (EC 3.1.3.16).	cgd6_4200.1.pdb	1auiA (HYDROLASE)	55	1.55	1tcoA (COMPLEX (HYDROLASE/ISOMERASE))	100	100	100	100	FK5	DB00864 (Tacrolimus)	DB00864 (Tacrolimus)	DB00864 (Tacrolimus)	DB00864 (Tacrolimus) DB00337 (Pimecrolimus)
Cryptosporidium parvum Iowa II.	Q5CX54	Phosphorylase (EC 2.4.1.1).	cgd6_2450.1.pdb	1ygpA (GLYCOSYLTRANSFERASE)	49	1.62	2ecpB (ACARBOSE)	100	100	100	100	ACR	DB00284 (Acarbose)	DB00284 (Acarbose)	DB00284 (Acarbose)	DB00284 (Acarbose)
Cryptosporidium parvum Iowa II.	Q5CY81	Eukaryotic DNA topoisomerase I (Fragment).	cgd7_3350.1.pdb	2b9sA (ISOMERASE/DNA)	47	1.16	1k4tA (ISOMERASE/DNA)	100	75	100	100	TTC	DB01030 (Topotecan)	DB01030 (Topotecan)	DB01030 (Topotecan)	DB00762 (Irinotecan) DB01030 (Topotecan)
Cryptosporidium parvum Iowa II.	Q5CY81	Eukaryotic DNA topoisomerase I (Fragment).	cgd7_3350.1.pdb	2b9sA (ISOMERASE/DNA)	47	1.16	1k4tA (ISOMERASE/DNA)	100	75	100	100	TTG				DB01030 (Topotecan)
Cryptosporidium parvum Iowa II.	Q5CYX1	Small nuclear ribonucleoprotein (Fragment).	cgd7_690.2.pdb	1jbmG (STRUCTURAL GENOMICS)	39	1.24	1lojC (RNA BINDING PROTEIN, TRANSCRIPTION)	100	100	85.71	100	URI				DB00322 (Floxuridine)
Trypanosoma cruzi.	Q5V9N6	Protein phosphatase 2B regulatory subunit (Calcineurin B subunit)(EC 3.1.3.16) (Calcineurin B subunit, putative).	Tc00.1047053510519.6.2.pdb	1auiB (HYDROLASE)	41	1.5	1tcoB (COMPLEX (HYDROLASE/ISOMERASE))	100	100	80	100	FK5	DB00864 (Tacrolimus)	DB00864 (Tacrolimus)	DB00864 (Tacrolimus)	DB00864 (Tacrolimus) DB00337 (Pimecrolimus)
Leishmania major.	Q6WRI4	Aromatic amino acid hydroxylase-like (Putative phenylalanine-4-hydroxylase, putative) (EC 1.14.16.1).	LmjF28.1280.2.pdb	1j8uA (OXIDOREDUCTASE)	51	1.48	3pah_ (MONOOXGYGENASE)	100	100	90	100	ALE	DB00668 (Epinephrine)	DB00388 (Phenylephrine) DB00668 (Epinephrine) DB00368 (Norepinephrine)	DB00668 (Epinephrine) DB01064 (Isoproterenol)	DB00388 (Phenylephrine) DB00668 (Epinephrine) DB00368 (Norepinephrine) DB01064 (Isoproterenol)
Leishmania major.	Q6WRI4	Aromatic amino acid hydroxylase-like (Putative phenylalanine-4-hydroxylase, putative) (EC 1.14.16.1).	LmjF28.1280.2.pdb	1j8uA (OXIDOREDUCTASE)	51	1.48	4pah_ (MONOOXGYGENASE)	100	100	100	100	NAR			DB01094 (Hesperetin)	DB01094 (Hesperetin)
Leishmania major.	Q6WRI4	Aromatic amino acid hydroxylase-like (Putative phenylalanine-4-hydroxylase, putative) (EC 1.14.16.1).	LmjF28.1280.2.pdb	1j8uA (OXIDOREDUCTASE)	51	1.48	5pah_ (MONOOXYGENASE)	100	100	100	100	LDP	DB00988 (Dopamine)	DB00988 (Dopamine)	DB00988 (Dopamine)	DB00988 (Dopamine)
Leishmania major.	Q6WRI4	Aromatic amino acid hydroxylase-like (Putative phenylalanine-4-hydroxylase, putative) (EC 1.14.16.1).	LmjF28.1280.2.pdb	1j8uA (OXIDOREDUCTASE)	51	1.48	6pah_ (MONOOXYGENASE)	100	100	100	100	DAH	DB01235 (Levodopa)	DB01235 (Levodopa)	DB01235 (Levodopa)	DB00765 (Metyrosine) DB01235 (Levodopa) DB00968 (Methyldopa)
Plasmodium falciparum	Q7K6A1	Histone deacetylase.	PFI1260c.2.pdb	1t64A (HYDROLASE)	42	1.45	1c3sA (LYASE)	83.33	80	90.91	100	SHH	DB02546 (Vorinostat)	DB02546 (Vorinostat)	DB02546 (Vorinostat)	DB02546 (Vorinostat)
Plasmodium falciparum	Q7KQM0	Triosephosphate isomerase (EC 5.3.1.1) (TIM) (Triose-phosphateisomerase).	PF14_0378.2.pdb	1o5xA (ISOMERASE)	100	2.28	1tpfB (ISOMERASE(INTRAMOLECULAR)	85.71	100	100	100	DMS	DB01093 (Dimethyl sulfoxide)	DB01093 (Dimethyl sulfoxide)	DB01093 (Dimethyl sulfoxide)	DB01093 (Dimethyl sulfoxide)
Trypanosoma cruzi.	Q7YUF0	Adenosylhomocysteinase (EC 3.3.1.1).	Tc00.1047053511589.2.2.pdb	1li4A (HYDROLASE)	75	1.96	1ky5D (HYDROLASE)	100	100	91.3	100	ADY				DB00194 (Vidarabine)
Trypanosoma cruzi.	Q7YUF0	Adenosylhomocysteinase (EC 3.3.1.1).	Tc00.1047053511589.2.2.pdb	1li4A (HYDROLASE)	75	1.96	1d4fD (HYDROLASE)	100	100	91.3	100	ADN			DB00194 (Vidarabine)	DB00242 (Cladribine) DB00194 (Vidarabine)
Plasmodium falciparum	Q8I2U5	Inosine-5'-monophosphate dehydrogenase (EC 1.1.1.205).	PFI1020c.1.pdb	1jr1A (OXIDOREDUCTASE)	42	1.13	1jr1B (OXIDOREDUCTASE)	100	100	100	100	MOA	DB01024 (Mycophenolic acid)	DB01024 (Mycophenolic acid)	DB00688 (Mycophenolate mofetil) DB01024 (Mycophenolic acid)	DB00688 (Mycophenolate mofetil) DB01024 (Mycophenolic acid)
Plasmodium falciparum	Q8I4S1	Thymidylate kinase, putative.	PFL2465c.2.pdb	3tmkA (KINASE)	41	1.49	2tmkB (PHOSPHOTRANSFERASE)	100	100	82.61	100	ATM		DB00495 (Zidovudine)		DB00495 (Zidovudine)
Plasmodium falciparum	Q8IAM8	Serine/threonine protein phosphatase (EC 3.1.3.16).	PF08_0129.1.pdb	1auiA (HYDROLASE)	54	1.42	1tcoA (COMPLEX (HYDROLASE/ISOMERASE))	100	100	100	100	FK5	DB00864 (Tacrolimus)	DB00864 (Tacrolimus)	DB00864 (Tacrolimus)	DB00864 (Tacrolimus) DB00337 (Pimecrolimus)
Plasmodium falciparum	Q8IAT5	Putative uncharacterized protein PF08_0099.	PF08_0099.1.pdb	1hbkA (FATTY ACID METABOLISM)	65	1.86	1hbkA (FATTY ACID METABOLISM)	100	100	75	100	MYR				DB00313 (Valproic Acid) DB00548 (Azelaic Acid)
Plasmodium falciparum	Q8IE67	Phosphoribosylpyrophosphate synthetase (EC 2.7.6.1).	PF13_0143.1.pdb	1dkuA (TRANSFERASE)	50	1.36	1dkuA (TRANSFERASE)	100	100	75	100	ABM				DB01073 (Fludarabine)
Plasmodium falciparum	Q8IJC7	Centrin, putative.	PF10_0271.2.pdb	1oqpA (PROTEIN BINDING)	61	1.19	1lin_ (CALCIUM-BINDING PROTEIN)	80	100	88.89	100	TFP	DB00831 (Trifluoperazine)	DB00831 (Trifluoperazine) DB00508 (Triflupromazine)	DB00831 (Trifluoperazine) DB00623 (Fluphenazine)	DB00831 (Trifluoperazine) DB00508 (Triflupromazine) DB00623 (Fluphenazine)
Plasmodium falciparum	Q8IJN7	Enolase (EC 4.2.1.11) (2-phosphoglycerate dehydratase) (2-phospho-D-glycerate hydro-lyase).	PF10_0155.3.pdb	1pdz_ (LYASE (CARBON-OXYGEN))	67	1.89	1lin_ (CALCIUM-BINDING PROTEIN)	80	100	100	100	LI	DB01356 (Lithium)	DB01356 (Lithium)	DB01356 (Lithium)	DB01356 (Lithium)
Plasmodium falciparum	Q8IJN7	Enolase (EC 4.2.1.11) (2-phosphoglycerate dehydratase) (2-phospho-D-glycerate hydro-lyase).	PF10_0155.2.pdb	2akzA (LYASE)	70	1.87	2oneA (LYASE)	100	100	100	100	LI	DB01356 (Lithium)	DB01356 (Lithium)	DB01356 (Lithium)	DB01356 (Lithium)
Plasmodium falciparum	Q8IK24	Acyl CoA binding protein, putative.	PF10_0016.1.pdb	1hbkA (FATTY ACID METABOLISM)	64	1.91	1hbkA (FATTY ACID METABOLISM)	100	100	75	100	MYR				DB00313 (Valproic Acid) DB00548 (Azelaic Acid)
Plasmodium falciparum	Q8IK25	Acyl CoA binding protein, putative.	PF10_0015.1.pdb	1hbkA (FATTY ACID METABOLISM)	75	1.95	1hbkA (FATTY ACID METABOLISM)	100	100	87.5	100	MYR				DB00313 (Valproic Acid) DB00548 (Azelaic Acid)
Plasmodium falciparum	Q8IK57	Acyl CoA binding protein.	PF14_0749.1.pdb	1hbkA (FATTY ACID METABOLISM)	100	2.24	1hbkA (FATTY ACID METABOLISM)	100	100	100	100	MYR				DB00313 (Valproic Acid) DB00548 (Azelaic Acid)
Plasmodium falciparum	Q8IKT2	6-phosphogluconate dehydrogenase, decarboxylating (EC 1.1.1.44).	PF14_0520.1.pdb	2pgd_ (OXIDOREDUCTASE (CHOH(D)-NADP+(A)))	45	1.66	1hbkA (FATTY ACID METABOLISM)	100	100	80	100	2AM				DB01073 (Fludarabine)
Plasmodium falciparum	Q8IKV9	Protein phosphatase 2b regulatory subunit, putative.	PF14_0492.2.pdb	1auiB (HYDROLASE)	51	1.62	1tcoB (COMPLEX (HYDROLASE/ISOMERASE))	100	100	100	100	FK5	DB00864 (Tacrolimus)	DB00864 (Tacrolimus)	DB00864 (Tacrolimus)	DB00864 (Tacrolimus) DB00337 (Pimecrolimus)
Plasmodium falciparum	Q8IL68	Delta-aminolevulinic acid dehydratase (EC 4.2.1.24).	PF14_0381.2.pdb	1w5qA (SYNTHASE)	35	1.28	1h7rA (DEHYDRATASE)	78.57	100	92.31	100	SHU				DB00548 (Azelaic Acid)
Plasmodium falciparum	Q8IL68	Delta-aminolevulinic acid dehydratase (EC 4.2.1.24).	PF14_0381.2.pdb	1w5qA (SYNTHASE)	35	1.28	1l6sA (LYASE)	82.35	94.44	81.25	100	DSB				DB00548 (Azelaic Acid)
Plasmodium falciparum	Q8IL68	Delta-aminolevulinic acid dehydratase (EC 4.2.1.24).	PF14_0381.2.pdb	1w5qA (SYNTHASE)	35	1.28	1b4kB (LYASE)	92.86	100	92.86	100	LEA			DB00510 (Divalproex sodium) DB00313 (Valproic Acid)	DB00510 (Divalproex sodium) DB00313 (Valproic Acid)
Plasmodium falciparum	Q8ILA4	Glucose-6-phosphate isomerase (EC 5.3.1.9).	PF14_0341.2.pdb	1t10A (ISOMERASE)	40	1.5	1iatA (ISOMERASE)	100	100	87.5	100	SUL			DB00653 (Magnesium Sulfate)	DB00653 (Magnesium Sulfate)
Plasmodium falciparum	Q8IM16	Plasmepsin, putative.	PF14_0075.1.pdb	1miqA (HYDROLASE)	76	1.88	3pep_ (HYDROLASE (ACID PROTEINASE))	83.33	100	80	100	EOH	DB00898 (Ethanol)	DB00898 (Ethanol)	DB00898 (Ethanol)	DB00898 (Ethanol)
Toxoplasma gondii.	Q8MQT6	Protein phosphatase 2B regulatory subunit.	31.m00003.2.pdb	1auiB (HYDROLASE)	53	1.65	1tcoB (COMPLEX (HYDROLASE/ISOMERASE))	100	100	100	100	FK5	DB00864 (Tacrolimus)	DB00864 (Tacrolimus)	DB00864 (Tacrolimus)	DB00864 (Tacrolimus) DB00337 (Pimecrolimus)
Plasmodium falciparum.	Q8MTV7	Delta-aminolevulinic acid dehydratase (EC 4.2.1.24).	PF14_0381.3.pdb	2c1hA (LYASE)	37	1.19	1b4eA (LYASE)	100	100	92.31	100	LEA			DB00510 (Divalproex sodium) DB00313 (Valproic Acid)	DB00510 (Divalproex sodium) DB00313 (Valproic Acid)
Plasmodium falciparum.	Q8MTV7	Delta-aminolevulinic acid dehydratase (EC 4.2.1.24).	PF14_0381.1.pdb	1h7nA (DEHYDRATASE)	27	1.1	1b4kB (LYASE)	92.86	100	92.31	100	LEA			DB00510 (Divalproex sodium) DB00313 (Valproic Acid)	DB00510 (Divalproex sodium) DB00313 (Valproic Acid)
Plasmodium falciparum.	Q8MTV7	Delta-aminolevulinic acid dehydratase (EC 4.2.1.24).	PF14_0381.3.pdb	2c1hA (LYASE)	37	1.19	1h7rA (DEHYDRATASE)	92.86	100	92.31	100	SHU				DB00548 (Azelaic Acid)
Plasmodium falciparum.	Q8MTV7	Delta-aminolevulinic acid dehydratase (EC 4.2.1.24).	PF14_0381.3.pdb	2c1hA (LYASE)	37	1.19	1l6sB (LYASE)	94.12	100	88.89	100	DSB				DB00548 (Azelaic Acid)
Plasmodium falciparum.	Q8MTV7	Delta-aminolevulinic acid dehydratase (EC 4.2.1.24).	PF14_0381.1.pdb	1h7nA (DEHYDRATASE)	27	1.1	1eb3A (DEHYDRATASE)	100	100	83.33	100	DSB				DB00548 (Azelaic Acid)
Plasmodium falciparum.	Q8MTV7	Delta-aminolevulinic acid dehydratase (EC 4.2.1.24).	PF14_0381.3.pdb	2c1hA (LYASE)	37	1.19	1h7oA (DEHYDRATASE)	93.33	100	85.71	100	DAV				DB00855 (Aminolevulinic acid)
Plasmodium falciparum.	Q8MTV7	Delta-aminolevulinic acid dehydratase (EC 4.2.1.24).	PF14_0381.3.pdb	2c1hA (LYASE)	37	1.19	1l6yA (LYASE)	80	100	85.71	100	4OX				DB00548 (Azelaic Acid)
Trypanosoma cruzi.	Q8T5T8	Dihydrofolate reductase-thymidylate synthase.	Tc00.1047053509153.9.2.pdb	1f28A (TRANSFERASE)	59	1.4	1f4fA (TRANSFERASE)	100	100	100	100	SUL			DB00653 (Magnesium Sulfate)	DB00653 (Magnesium Sulfate)
Trypanosoma cruzi.	Q8T5T8	Dihydrofolate reductase-thymidylate synthase.	Tc00.1047053509153.9.2.pdb	1f28A (TRANSFERASE)	59	1.4	1dduB (METHYLTRANSFERASE)	100	100	100	100	DDU			DB00322 (Floxuridine) DB00432 (Trifluridine)	DB00322 (Floxuridine) DB00432 (Trifluridine)
Trypanosoma cruzi.	Q8T5T8	Dihydrofolate reductase-thymidylate synthase.	Tc00.1047053509153.9.2.pdb	1f28A (TRANSFERASE)	59	1.4	2tsrD (METHYLTRANSFERASE)	100	100	80.77	100	D16	DB00293 (Raltitrexed)	DB00293 (Raltitrexed)	DB00293 (Raltitrexed)	DB00293 (Raltitrexed)
Trypanosoma cruzi.	Q8T5T8	Dihydrofolate reductase-thymidylate synthase.	Tc00.1047053509153.9.2.pdb	1f28A (TRANSFERASE)	59	1.4	2tdd_ (TRANSFERASE (METHYLTRANFERASE))	93.33	100	95.45	100	UFP		DB00322 (Floxuridine)		DB00322 (Floxuridine)
Trypanosoma cruzi.	Q8T5T8	Dihydrofolate reductase-thymidylate synthase.	Tc00.1047053509153.9.2.pdb	1f28A (TRANSFERASE)	59	1.4	1tduA (METHYLTRANSFERASE)	94.12	100	94.12	100	DUR			DB00322 (Floxuridine) DB00432 (Trifluridine)	DB00322 (Floxuridine) DB00432 (Trifluridine)
Trypanosoma cruzi.	Q8T5T8	Dihydrofolate reductase-thymidylate synthase.	Tc00.1047053509153.9.2.pdb	1f28A (TRANSFERASE)	59	1.4	1ju6D (TRANSFERASE)	94.12	100	89.47	100	LYA	DB00642 (Pemetrexed)	DB00642 (Pemetrexed)	DB00642 (Pemetrexed)	DB00642 (Pemetrexed)
Trypanosoma cruzi.	Q8T5T8	Dihydrofolate reductase-thymidylate synthase.	Tc00.1047053509153.9.2.pdb	1f28A (TRANSFERASE)	59	1.4	1jutB (TRANSFERASE)	80	100	85.71	100	LYD				DB00642 (Pemetrexed)
Trypanosoma brucei brucei.	Q8WSK3	Protein kinase A catalytic subunit.	Tb10.389.0490.3.pdb	1rdqE (TRANSFERASE/TRANSFERASE INHIBITOR)	51	1.66	1fmoE (COMPLEX (PHOSPHOTRANSFERASE/INHIBITOR))	100	100	88.89	100	ADN			DB00194 (Vidarabine)	DB00242 (Cladribine) DB00194 (Vidarabine)
Plasmodium falciparum.	Q94650	ADP-ribosylation factor 1.	PF10_0203.2.pdb	1mr3F (SIGNALING PROTEIN)	73	2.05	1mr3F (SIGNALING PROTEIN)	100	100	84.62	100	EOH	DB00898 (Ethanol)	DB00898 (Ethanol)	DB00898 (Ethanol)	DB00898 (Ethanol)
Trypanosoma brucei.	Q964F1	Aspartate aminotransferase (EC 2.6.1.1).	Tb10.70.3710.2.pdb	2cstA (TRANSFERASE(AMINOTRANSFERASE))	46	1.7	2ay2B (AMINOTRANSFERASE)	78.57	100	84.62	100	CXP				DB00548 (Azelaic Acid)
Mycobacterium leprae.	Q9CB18	Glucose-1-phosphate thymidylyltransferase (EC 2.7.7.24) (dTDP-glucosepyrophosphorylase) (dTDP-glucose synthase).	13094048.1.pdb	1iimA (TRANSFERASE)	60	1.81	1h5rC (TRANSFERASE)	100	100	77.14	100	THM			DB00432 (Trifluridine)	DB00322 (Floxuridine) DB00432 (Trifluridine)
Mycobacterium leprae.	Q9CB40	Mycolic acid synthase.	13094024.1.pdb	1tpyA (TRANSFERASE)	72	2.01	1kpgA (TRANSFERASE)	78.26	100	77.27	100	16A				DB01245 (Decamethonium)
Mycobacterium leprae.	Q9CBM7	Methylmalonyl-CoA mutase, [alpha] subunit.	13093511.1.pdb	1reqA (ISOMERASE)	70	1.83	4reqA (ISOMERASE)	100	100	100	100	5AD			DB00194 (Vidarabine)	DB00242 (Cladribine) DB00194 (Vidarabine)
Mycobacterium leprae.	Q9CBW0	Thymidylate synthase (EC 2.1.1.45) (TS) (TSase).	13093353.1.pdb	1tsy_ (TRANSFERASE (METHYLTRANSFERASE))	59	1.68	4reqA (ISOMERASE)	100	100	77.27	86.36	MTX	DB00563 (Methotrexate)	DB00563 (Methotrexate)	DB00563 (Methotrexate)	DB00563 (Methotrexate)
Mycobacterium leprae.	Q9CBW0	Thymidylate synthase (EC 2.1.1.45) (TS) (TSase).	13093353.1.pdb	1tsy_ (TRANSFERASE (METHYLTRANSFERASE))	59	1.68	4reqA (ISOMERASE)	100	100	84.62	84.62	LYD				DB00642 (Pemetrexed)
Mycobacterium leprae.	Q9CBW0	Thymidylate synthase (EC 2.1.1.45) (TS) (TSase).	13093353.2.pdb	2g8mA (TRANSFERASE)	66	1.94	1jtuA (TRANSFERASE)	94.74	100	84.21	100	LYB				DB00642 (Pemetrexed)
Mycobacterium leprae.	Q9CBW0	Thymidylate synthase (EC 2.1.1.45) (TS) (TSase).	13093353.2.pdb	2g8mA (TRANSFERASE)	66	1.94	1axwB (METHYLTRANSFERASE)	95.45	100	82.61	100	MTX	DB00563 (Methotrexate)	DB00563 (Methotrexate)	DB00563 (Methotrexate)	DB00563 (Methotrexate)
Mycobacterium leprae.	Q9CBW0	Thymidylate synthase (EC 2.1.1.45) (TS) (TSase).	13093353.2.pdb	2g8mA (TRANSFERASE)	66	1.94	1dduB (METHYLTRANSFERASE)	100	100	76.92	100	DDU			DB00322 (Floxuridine) DB00432 (Trifluridine)	DB00322 (Floxuridine) DB00432 (Trifluridine)
Mycobacterium leprae.	Q9CBW0	Thymidylate synthase (EC 2.1.1.45) (TS) (TSase).	13093353.1.pdb	1tsy_ (TRANSFERASE (METHYLTRANSFERASE))	59	1.68	4reqA (ISOMERASE)	100	100	95.24	95.24	UFP		DB00322 (Floxuridine)		DB00322 (Floxuridine)
Mycobacterium leprae.	Q9CBW0	Thymidylate synthase (EC 2.1.1.45) (TS) (TSase).	13093353.1.pdb	1tsy_ (TRANSFERASE (METHYLTRANSFERASE))	59	1.68	4reqA (ISOMERASE)	100	100	81.82	90.91	D16	DB00293 (Raltitrexed)	DB00293 (Raltitrexed)	DB00293 (Raltitrexed)	DB00293 (Raltitrexed)
Mycobacterium leprae.	Q9CBW0	Thymidylate synthase (EC 2.1.1.45) (TS) (TSase).	13093353.2.pdb	2g8mA (TRANSFERASE)	66	1.94	1tlsB (METHYLTRANSFERASE)	94.74	100	80.95	100	UFP		DB00322 (Floxuridine)		DB00322 (Floxuridine)
Mycobacterium leprae.	Q9CBW0	Thymidylate synthase (EC 2.1.1.45) (TS) (TSase).	13093353.2.pdb	2g8mA (TRANSFERASE)	66	1.94	1jutB (TRANSFERASE)	93.33	100	92.86	100	LYD				DB00642 (Pemetrexed)
Mycobacterium leprae.	Q9CBW0	Thymidylate synthase (EC 2.1.1.45) (TS) (TSase).	13093353.1.pdb	1tsy_ (TRANSFERASE (METHYLTRANSFERASE))	59	1.68	4reqA (ISOMERASE)	100	100	100	100	DDU			DB00322 (Floxuridine) DB00432 (Trifluridine)	DB00322 (Floxuridine) DB00432 (Trifluridine)
Mycobacterium leprae.	Q9CBW0	Thymidylate synthase (EC 2.1.1.45) (TS) (TSase).	13093353.2.pdb	2g8mA (TRANSFERASE)	66	1.94	1tduA (METHYLTRANSFERASE)	94.12	100	82.35	100	DUR			DB00322 (Floxuridine) DB00432 (Trifluridine)	DB00322 (Floxuridine) DB00432 (Trifluridine)
Mycobacterium leprae.	Q9CBW0	Thymidylate synthase (EC 2.1.1.45) (TS) (TSase).	13093353.1.pdb	1tsy_ (TRANSFERASE (METHYLTRANSFERASE))	59	1.68	4reqA (ISOMERASE)	100	100	93.75	93.75	DUR			DB00322 (Floxuridine) DB00432 (Trifluridine)	DB00322 (Floxuridine) DB00432 (Trifluridine)
Mycobacterium leprae.	Q9CBW0	Thymidylate synthase (EC 2.1.1.45) (TS) (TSase).	13093353.2.pdb	2g8mA (TRANSFERASE)	66	1.94	2kceA (METHYLTRANSFERASE)	95.45	100	87.5	100	D16	DB00293 (Raltitrexed)	DB00293 (Raltitrexed)	DB00293 (Raltitrexed)	DB00293 (Raltitrexed)
Mycobacterium leprae.	Q9CBW1	Dihydrofolate reductase (EC 1.5.1.3).	13093352.2.pdb	1df7A (OXIDOREDUCTASE)	72	1.92	1df7A (OXIDOREDUCTASE)	100	100	76.92	100	MTX	DB00563 (Methotrexate)	DB00563 (Methotrexate)	DB00563 (Methotrexate)	DB00563 (Methotrexate)
Mycobacterium leprae.	Q9CBW1	Dihydrofolate reductase (EC 1.5.1.3).	13093352.2.pdb	1df7A (OXIDOREDUCTASE)	72	1.92	1dg5A (OXIDOREDUCTASE)	100	100	93.33	100	TOP	DB00440 (Trimethoprim)	DB00440 (Trimethoprim)	DB00440 (Trimethoprim)	DB00440 (Trimethoprim)

Organism	UniProt Id	Target description	3D model id	PDB Template	Model sequence identity	ModPipe Quality Score	PDB Expansion id	Binding site expansion sequence identity	Binding site expansion structure identity	Binding site sequence identity	Binding site structure identity	PDB Ligand id	Drugbank (Exact)	Drugbank (supra-structure)	Drugbank (sub-structure)	Drugbank (similar)
Mycobacterium leprae.	Q9CBZ0	Nucleoside diphosphate kinase (EC 2.7.4.6) (NDK) (NDP kinase)(Nucleoside-2-P kinase).	13093323.1.pdb	1wkjA (TRANSFERASE)	58	1.78	1nhkR (PHOSPHOTRANSFERASE)	92.31	100	75	100	CMP				DB01073 (Fludarabine)
Mycobacterium leprae.	Q9CCG4	Putative glycyl-tRNA synthase.	13092913.1.pdb	1atiA (PROTEIN BIOSYNTHESIS)	45	1.39	1ggmB (AMINOACYL-TRNA SYNTHASE)	100	100	85.19	100	GAP				DB01073 (Fludarabine)
Mycobacterium leprae.	Q9CCJ3	Putative thymidylate kinase.	13092882.1.pdb	1gtvA (TRANSFERASE)	77	2.04	1g3uA (TRANSFERASE)	90	100	100	100	SUL			DB00653 (Magnesium Sulfate)	DB00653 (Magnesium Sulfate)
Mycobacterium leprae.	Q9CCJ4	Adenosylhomocysteinase (EC 3.3.1.1) (S-adenosyl-L-homocysteinehydrolase) (AdoHcyase).	13092881.2.pdb	1v8bA (HYDROLASE)	56	1.58	1d4fB (HYDROLASE)	91.3	100	86.96	100	ADN			DB00194 (Vidarabine)	DB00242 (Cladribine) DB00194 (Vidarabine)
Mycobacterium leprae.	Q9CCJ4	Adenosylhomocysteinase (EC 3.3.1.1) (S-adenosyl-L-homocysteinehydrolase) (AdoHcyase).	13092881.1.pdb	1li4A (HYDROLASE)	62	1.66	1ky5D (HYDROLASE)	100	100	91.3	100	ADY				DB00194 (Vidarabine)
Mycobacterium leprae.	Q9CCJ4	Adenosylhomocysteinase (EC 3.3.1.1) (S-adenosyl-L-homocysteinehydrolase) (AdoHcyase).	13092881.1.pdb	1li4A (HYDROLASE)	62	1.66	1d4fD (HYDROLASE)	100	100	91.3	100	ADN			DB00194 (Vidarabine)	DB00242 (Cladribine) DB00194 (Vidarabine)
Mycobacterium leprae.	Q9CCP4	Putative GTP cyclohydrolase II/3,4-dihydroxy-2-butanone-4-phosphatesynthase (EC 3.5.4.25).	13092758.1.pdb	1g57A (ISOMERASE)	47	1.16	1k4oA (ISOMERASE)	80	100	88.89	100	SUL			DB00653 (Magnesium Sulfate)	DB00653 (Magnesium Sulfate)
Mycobacterium leprae.	Q9CCS3	3-dehydroquinate dehydratase (EC 4.2.1.10) (3-dehydroquinase) (Type IIDHQase).	13092729.1.pdb	1uqrA (LYASE)	46	1.69	1j2yA (LYASE)	78.57	93.33	84.62	100	DQA				DB00326 (Calcium Gluceptate)
Mycobacterium leprae.	Q9CD24	Aminoglycoside 2'-N-acetyltransferase.	13093802.1.pdb	1m4iA (TRANSFERASE)	79	2.03	1m4iA (TRANSFERASE)	100	100	76.47	100	KAN	DB01172 (Kanamycin)	DB01172 (Kanamycin)	DB01172 (Kanamycin)	DB01172 (Kanamycin) DB00684 (Tobramycin)
Mycobacterium leprae.	Q9CD45	Ribose-phosphate pyrophosphokinase (EC 2.7.6.1) (RPPK) (Phosphoribosylpyrophosphate synthetase) (P-Rib-PP synthetase) (PRPP synthetase).	13092591.1.pdb	1dkuA (TRANSFERASE)	46	1.54	1dkuA (TRANSFERASE)	100	100	75	100	ABM				DB01073 (Fludarabine)
Mycobacterium leprae.	Q9CD75	Glucose-6-phosphate isomerase (EC 5.3.1.9) (GPI) (Phosphoglucoseisomerase) (PGI) (Phosphohexose isomerase) (PHI).	13092520.1.pdb	1iatA (ISOMERASE)	49	1.62	1iatA (ISOMERASE)	100	100	80	100	SUL			DB00653 (Magnesium Sulfate)	DB00653 (Magnesium Sulfate)
Leishmania major.	Q9GN16	Putative uncharacterized protein PKAC2a (EC 2.7.1.37).	LmjF35.3960.2.pdb	1rdqE (TRANSFERASE/TRANSFERASE INHIBITOR)	48	1.67	1fmoE (COMPLEX (PHOSPHOTRANSFERASE/INHIBITOR))	100	100	88.89	100	ADN			DB00194 (Vidarabine)	DB00242 (Cladribine) DB00194 (Vidarabine)
Cryptosporidium parvum.	Q9GU59	Putative histone deacetylase.	cgd6_1380.1.pdb	1t64A (HYDROLASE)	37	1.47	1c3sA (LYASE)	83.33	80	90.91	100	SHH	DB02546 (Vorinostat)	DB02546 (Vorinostat)	DB02546 (Vorinostat)	DB02546 (Vorinostat)
Leishmania major.	Q9U0W1	Acetyl-CoA synthetase (Acetyl-CoA synthetase, putative) (EC 6.2.1.1).	LmjF23.0710.2.pdb	1pg4A (LIGASE)	54	1.7	1pg4B (LIGASE)	100	100	90.48	100	PRX		DB00194 (Vidarabine)		DB01073 (Fludarabine) DB00194 (Vidarabine)
Leishmania major.	Q9U1E1	Nucleoside diphosphate kinase B (EC 2.7.4.6).	LmjF32.2950.1.pdb	1npk_ (PHOSPHOTRANSFERASE(PO4 AS ACCEPTOR))	59	1.78	1pg4B (LIGASE)	100	100	83.33	100	CMP				DB01073 (Fludarabine)
Leishmania major.	Q9Y0F3	3-hydroxy-3-methylglutaryl coenzyme A reductase (3-hydroxy-3-methylglutaryl-CoA reductase, putative).	LmjF30.3190.1.pdb	1dqaA (OXIDOREDUCTASE)	52	1.64	1hw8D (OXIDOREDUCTASE)	100	100	78.26	100	114			DB00175 (Pravastatin)	DB00641 (Simvastatin) DB00175 (Pravastatin) DB00227 (Lovastatin)
Leishmania major.	Q9Y0F3	3-hydroxy-3-methylglutaryl coenzyme A reductase (3-hydroxy-3-methylglutaryl-CoA reductase, putative).	LmjF30.3190.1.pdb	1dqaA (OXIDOREDUCTASE)	52	1.64	1hwkD (OXIDOREDUCTASE)	100	100	84	100	117	DB01076 (Atorvastatin)	DB01076 (Atorvastatin)	DB01076 (Atorvastatin)	DB01076 (Atorvastatin)
Leishmania major.	Q9Y0F3	3-hydroxy-3-methylglutaryl coenzyme A reductase (3-hydroxy-3-methylglutaryl-CoA reductase, putative).	LmjF30.3190.1.pdb	1dqaA (OXIDOREDUCTASE)	52	1.64	1hw9D (OXIDOREDUCTASE)	100	100	78.26	100	SIM				DB00641 (Simvastatin) DB00175 (Pravastatin) DB00227 (Lovastatin)
